# Computer-Aided Retrosynthesis for Greener and Optimal Total Synthesis of a Helicase-Primase Inhibitor Active Pharmaceutical Ingredient

**DOI:** 10.1021/jacsau.4c00624

**Published:** 2024-10-02

**Authors:** Rodolfo I. Teixeira, Michael Andresini, Renzo Luisi, Brahim Benyahia

**Affiliations:** † Department of Chemical Engineering, Loughborough University, Loughborough LE11 3TU, U.K.; ‡ Department of Pharmacy−Drug Sciences, University of Bari “A. Moro”, Bari 70125, Italy

**Keywords:** computer-aided retrosynthesis, computer-assisted synthesis planning, drug design, green-by-design, green chemistry, pharmaceuticals, total synthesis, helicase-primase inhibitor

## Abstract

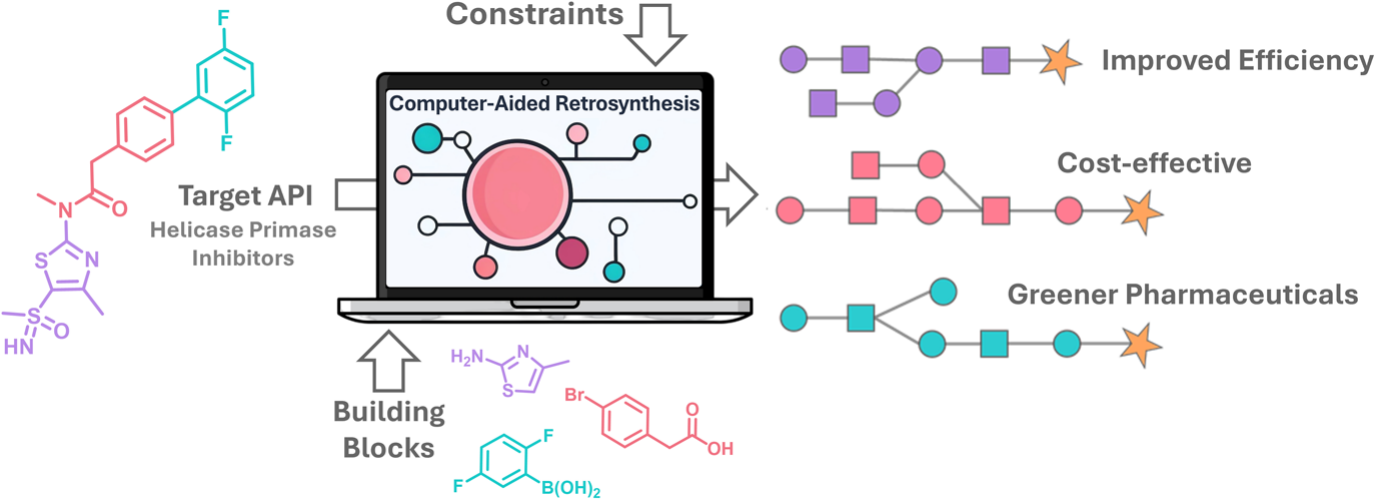

This study leverages and upgrades the capabilities of computer-aided retrosynthesis (CAR) in the systematic development of greener and more efficient total synthetic routes for the active pharmaceutical ingredient (API) IM-204, a helicase-primase inhibitor that demonstrated enhanced efficacy against Herpes simplex virus (HSV) infections. Using various CAR tools, several total synthetic routes were uncovered, evaluated, and experimentally validated, with the goal to maximize selectivity and yield and minimize the environmental impact. The CAR tools revealed several synthetic options under different constraints, which can overperform the patented synthetic route used as a reference. The selected CAR-based route demonstrated a significant improvement of the total yield from 8% (patented route) to 26%, along with a moderate improvement in the overall green performance. It was also shown that a human-in-the-loop approach can be synergistically combined with CAR to drive further improvements and deliver greener synthetic alternatives. This strategy further enhanced the green metrics by substituting solvents and merging two steps into one. These changes led to a significant improvement in the overall yield of IM-204 synthesis from 8 to 35%. Additionally, the green performance score, based on the GreenMotion metrics, was improved from 0 to 18, and the total cost of the building blocks was reduced by 550-fold. This work demonstrates the potential of CAR in drug development, highlighting its capacity to streamline synthesis processes, reduce environmental footprint, and lower production costs, thereby advancing the field toward more efficient and sustainable practices.

## Introduction

The integration of AI-based and machine-learning techniques is revolutionizing and accelerating chemistry-related research and development,^1^ particularly in advancing methodologies such as molecular design, synthesis planning, and retrosynthesis.^2−101^ The latter, a pivotal technique in organic chemistry, involves devising strategies to construct target molecules from readily available or easily synthesized precursors. This method has been fundamental for chemists to create pathways for the synthesis of active pharmaceutical ingredients (APIs), natural products, and other complex molecules. Historically, determining the most efficient retrosynthetic routes to these desired molecules has posed significant challenges for synthetic chemists since it requires specialized knowledge of what bonds can and cannot be made and a wide knowledge of synthetic methodologies.

Recent developments have seen the emergence of computational tools designed to tackle retrosynthetic analysis problems, including Synthia (formerly Chematica),^6^ Reaxys Retrosynthesis,^7^ ASKCOS,^8^ CAS ChemPlanner^9^ AiZynthFinder,^10^ IBM RXN,^11,12^ and others.^3,13^ Notably, Synthia has demonstrated considerable success by employing a hybrid approach that integrates expertly coded rules with machine learning algorithms.^14−20^ In a seminal study, Grzybowski and colleagues illustrated Synthia’s capability to design synthetic routes for drug synthesis targeting medically relevant targets, such as dronedarone and engelheptanoxide.^18^ Subsequently, Grzybowski et al. further showcased that Synthia could generate plausible pathways for complex natural products, rivaling those conceived by experienced synthetic chemists. This led to the successful laboratory synthesis of three computer-designed natural products.^17^ Further, Cernak and co-workers have shown the use of computer-aided retrosynthesis to identify a key step and perform the total synthesis of complex alkaloids.^16^ This research team has also applied retrosynthetic design to the development of coronavirus drugs at the research stage, utilizing Synthia to discover synthetic pathways for candidate drug molecules, leading to successful experimental validation of Umifenovir and Bromhexine.^15^ Furthermore, Grzybowski and co-workers recently showed that retrosynthesis tools can be used to help convert wastes into medically relevant targets.^21^ The latter examples underscore the significant impact of retrosynthetic tools on advancing drug research and development.

The development of a new drug is a lengthy and costly process that commonly requires between 8 and 12 years and costs up to 2.8 billion dollars.^22^ The research and development in pharmaceutical manufacturing can vastly benefit from the emerging computer-aided technologies, allowing a more effective process and plant-wide design and integration^23,24^ and reduced environmental footprints. Computer-aided retrosynthesis (CAR) has the power to identify more efficient and more sustainable synthetic pathways for the targeted API. Furthermore, CAR can allow early recognition of incompatibilities and nonviable or risky synthetic steps in the development process.

Moreover, pharmaceutical manufacturing exhibits the largest environmental factor (E-factor) of all sectors, while it is considered one of the most solvent-intensive industries. Most of the environmental emission hot spots are associated with the production and purification of the active pharmaceutical ingredient (API) (i.e., upstream processing). The identification of effective and greener synthetic routes for API is a critical step in the development of safe and efficacious drugs. It is important to reduce the required reaction steps, maximize yield, and most importantly identify greener pathways and minimize the production of side products and impurities. Achieving these goals is challenging and often requires expensive and extensive experiments. CAR tools can help address these challenges by reducing reliance on the traditional intensive experimentation and consequently reducing development and production costs.^25,26^

Self-driving laboratories (SDLs) have recently emerged as one of the most significant technological developments in the chemical sciences, holding the potential to revolutionize the research process by quickly scrutinizing and optimizing conditions while assuring high reproducibility.^27−32^ The combination of CAR technology to inform automated synthesis can effectively contribute to SDLs, accelerating discovery and minimizing experimentation. In this space, IBM has been working on integrating its IBM RXn platform with robotic systems to deliver automated synthesis, combining CAR with advanced automation. In addition, several AI tools have been developed to complement CAR capabilities, such as advanced drug prediction algorithms,^33,34^ yield prediction models,^35,36^ self-optimization methods,^37,38^ and route selection systems.^39^ These tools can consolidate and enhance the efficiency and precision of CAR, enabling more efficient exploration of chemical space and ultimately contributing to SDLs.

Despite the potential advantages of CAR in the development of more effective total synthetic routes for APIs, its wide adoption remains very limited. Furthermore, its combination with green chemistry and sustainability paradigms has not been investigated to date. This study aims to deploy computer-aided retrosynthesis (CAR) beyond state of the art to guarantee greener-by-design and more effective total synthesis of a key pharmaceutical, helicase-primase inhibitor API (IM-204). The primary goal is to develop more systematic and effective methods to identify effective, greener, and experimentally viable synthetic routes, optimizing operational conditions to achieve higher selectivity and yield.

## Case Study

**Figure 1:**
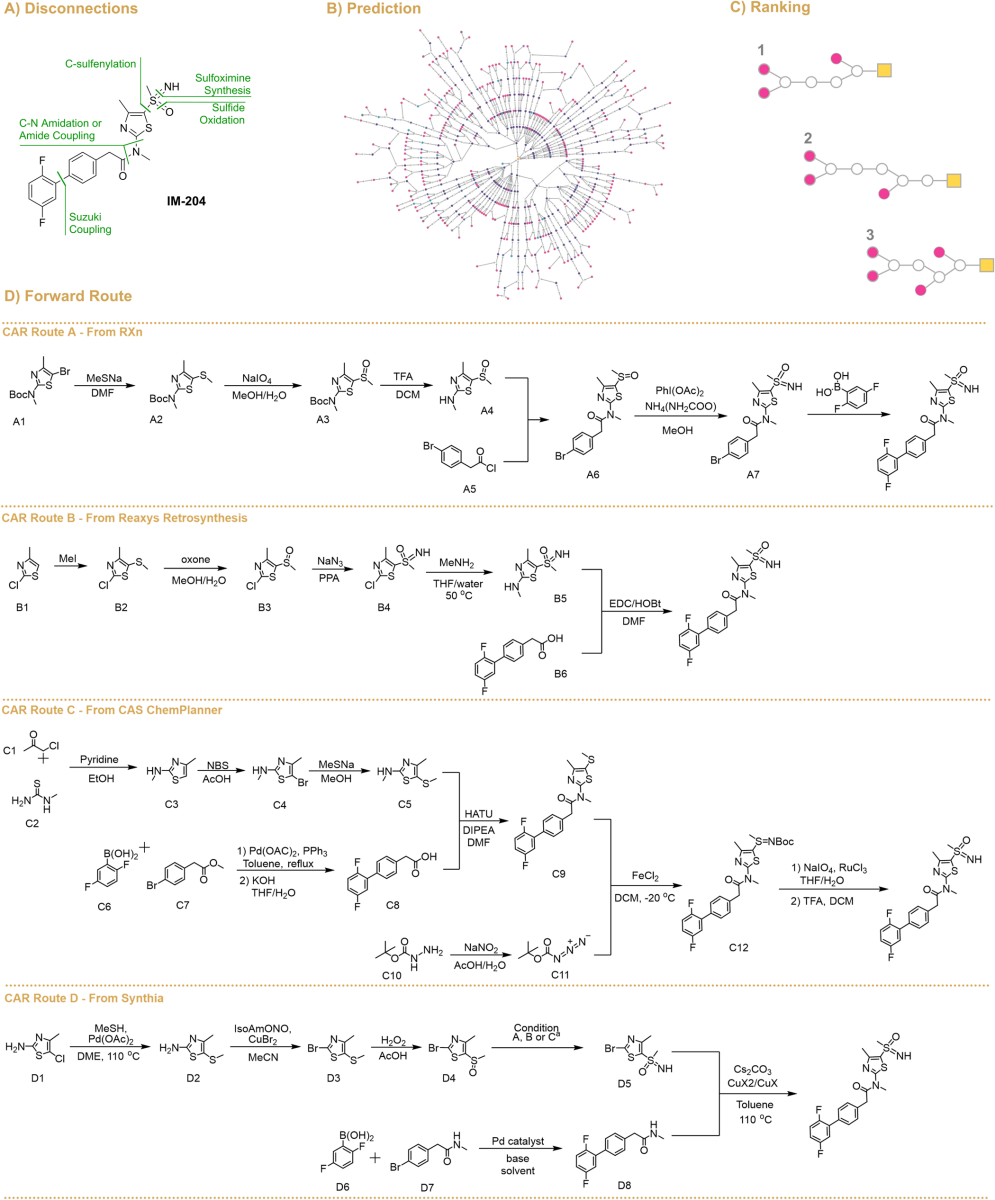
(A) General retrosynthetic analysis illustrating the main disconnection used by the different CAR tools. (B) Polar graph example of 50 best CAR-generated routes obtained from CAR route D. (C) General representation of reaction ranking. (D) Best scoring routes proposed by different CAR tools. ^a^ conditions for the conversion of **D4** into **D5**: (A) NaN_3_, Eaton’s reagent, 50°C; (B) NH_3_ source, PhI(OAc)_2_, MeOH; (C) FeSO_4_, 1,10-phen, NbzONH_2_·TfOH, MeCN.

The focus of this research is on a novel helicase-primase inhibitor, named IM-204 (Figure 1A), which is the racemic mixture of IM-250 that has shown promising results against both acute and chronic neural Herpes simplex virus (HSV) infections. Although the IM-250 is the most effective enantiomer, the racemic mixture has also shown promising activity.^40^ HSV is a significant global health concern. According to the World Health Organization (WHO), in 2016, around 491.5 million people were living with HSV-2 infection, which represents 13.2% of the global population aged 15 to 49. These statistics underscore the WHO’s call for effective treatments and preventative strategies for HSV. IM-250 distinctively targets the helicase-primase complex crucial for HSV DNA replication.^41^ Unlike existing medications that primarily address active herpes outbreaks, IM-250 presents a potential solution for latent HSV, which is responsible for recurrent symptoms in approximately 30% of infected individuals. Its effectiveness has recently been demonstrated based on in vivo latency/reactivation animal models, and it is currently undergoing phase-I clinical trials.^42^ Developing an effective, safe, and viable synthetic pathway for IM-204 is critical to enable its mass production and commercial availability. More importantly, considering the anticipated large demand for effective treatments for HSV, the development of a greener-by-design total synthetic route will be critical, allowing more environmentally friendly pharmaceutical manufacturing. To achieve these objectives, CAR and green chemistry are synergistically combined to streamline the development of this process, expediting the development phase and leading to reduced manufacturing costs.

## Computer-Aided Retrosynthesis

A generic framework for retrosynthesis planning consists of a reaction rule library, a chemical database with commercially available starting materials, and strategies that select bond disconnection rules. Changing these databases and rules typically leads to considerable changes in the outputs. Therefore, multiple retrosynthetic tools were tested using the target API, IM-204, to evaluate which tools would provide a reliable and safe synthetic pathway while guaranteeing optimal and reliable CAR results. Consequently, the state-of-the-art IBM’s Rxn, CAS ChemPlanner Retrosynthesis (formerly ARChem), Merck Synthia (formerly Chematica), and Elsevier’s Reaxys Retrosynthesis were tested. Each of the proposed CAR tools was used to identify the synthetic routes for IM-204 based on the settings and options available in the software for comparison. The CAR tools can be set to deliver a desired maximum number of synthetic routes (e.g., 10, 50, 100, etc.), which are evaluated and ranked based on a built-in overall key performance score.

Although the built-in scoring functions may differ between CAR tools (purely AI, hybrid knowledge-based/AI combinations, or heuristic), they all tend to rank the reaction pathways based on the highest probability of success within the search parameters based on similar cases on the training database. The differences on the output of the CAR can be mainly attributed to the different retrosynthesis model rules^43,44^ and in the data set available to build the forward reaction. For example, if a specific reaction (e.g., Buchwald–Hartwig amination) has a high occurrence for many substrates, it will lead to a high score. However, if the target has a functional group that is not tolerated by the suggested CAR reaction or needs protection, the score will suffer a penalty. The CAR also has an embedded similarity search for each proposed step to find the most similar reaction in the database to suggest plausible reaction conditions for the desired reaction.

After the different CAR tools were implemented, the top-ranking synthetic routes were extracted and analyzed based on a human-in-the-loop approach. Overall, all the CAR tools proposed similar retrosynthetic disconnections (molecule fragmentation) for IM-204, as represented in Figure 1A, to generate different synthetic pathways (Figure 1B). The main differences were in the forward reactions proposed for those disconnections. It is worth noting that the main disconnection centers are around the sulfoximine group and the amide bond. The routes are then ranked (Figure 1C), and the best-scoring routes from each CAR tool (Figure 1D) were then analyzed to determine which would be experimentally validated.

IBM Rxn proposed a 6-step synthetic route (CAR Route A, Figure 1D), starting from Boc-protected 5-bromo-*N*,4-dimethyl-2-aminothiazole (**A1**, Figure 1D), which was not commercially available in the UK. The compound undergoes C-sulfenylation to give **A2**, followed by sulfide oxidation to produce **A3** using sodium periodate. Interestingly, other CAR tools suggested that protection would not be required (see, for example, reactions from **C4** to **C5** and **D1** to **D2**). After deprotection, sulfoxide **A4** would then undergo amide coupling from acyl chloride **A5** to generate **A6**. Notably, Suzuki coupling was suggested after sulfoximine formation, and conditions were not given by the CAR for this step. By applying constraints on the maximum price of the starting materials, the CAR predicted a 7-step route that expands the route to synthesize Boc-protected *N*,4-dimethylthiazole from available starting materials. Besides the additional step, the nonprotected compound is commercially available in the UK at high costs (£250/g), making it unviable for this investigation.

Reaxys Retrosynthesis identified a 5-step route for the synthesis of the desired API (CAR Route B, Figure 1D). CAR route B consists of the synthesis of the aryl sulfide from 2-chloro-4-methylthiazole (**B1**) and methyl iodide only; the sulfur source required for the incorporation of a sulfide group was provided on the CAR output. This reaction possibly proceeds through a lithiation reaction, although details on conditions were not provided by the CAR tool. In addition, the top similarity examples for this step are for a sulfonyl chloride product instead of a sulfide synthesis (see Figure S2). In the next step, sulfide **B2** is oxidized to sulfoxide (**B3**) and then converted into sulfoximine (**B4**). Here, again, the similarity examples that were suggested do not show the correct functional group transformation (Figure S3), suggesting the preparation of a sulfone instead of a sulfimine. This suggests that improvements in the similarity score are needed. Sulfoximine **B4** is then proposed to undergo Buchwald amination, followed by amide coupling to deliver IM-204.

**Figure 2:**
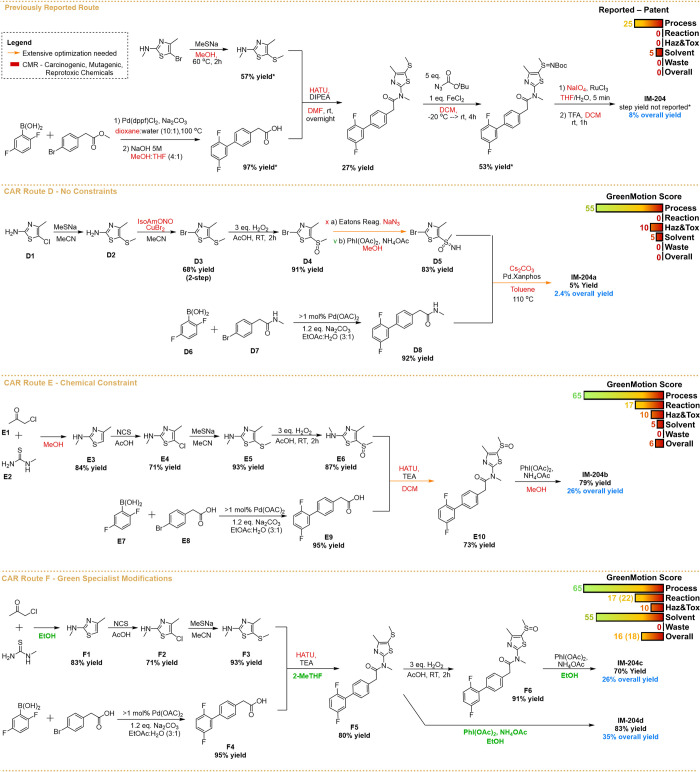
Scheme comparing the patented pathway to the synthesis of IM-204 with CAR routes experimentally validated. CAR route **D** shows experimental conditions and yields obtained when no constraints were applied on CAR software. CAR route E shows experimental conditions and yields obtained when the CAR search was constrained by making the synthesis of the heterocycle compulsory. CAR route **F** shows small human-in-the loop modifications (on green) focused mainly on the solvent. The inset on the CAR routes shows the GreenMotion scores obtained for each route. For the GreenMotion score in the previous reported route, not reported yields were considered quantitative yields (100%), and in the scores for CAR route F, the values for the path with the direct conversion of **F5** into IM-204 are shown in parentheses when different.

CAS ChemPlanner Retrosynthesis only provides a retrosynthesis analysis with a synthetic depth of 4 steps, and the proposed route for the target API resulted in a feasible route. By combining steps on the CAR results classified as “experimental” and “predicted”, a 10-step route was suggested for IM-204 (CAR Route C, Figure 1). Briefly, *N*,4-dimethyl-5-(methylthio)-2-aminothiazole (**C5**) is coupled with the respective carboxylic acid (**C8**) to form **C9**, which then reacts with 1,1-dimethylethyl carbonazidate (**C11**) to give the protected Boc-sulfimine (**C12**). Compound **C12** is then oxidized, and the imine is deprotected to produce the final sulfoximine API. The proposed route is highly similar to the route published for this compound (see Figure 2),^45,46^ and a trend on the CAR was noticed toward presenting only results that were previously published, even after changing the “evidence” scoring weight to zero. Also, only the top-scoring route is given, and CAS ChemPlanner does not show alternative routes (lower-scoring routes). Although the use of reactions previously reported allows for robust results when the aim is solely to prepare a target compound, it can be a drawback when seeking alternative synthetic routes for a target molecule.

Synthia offered a reasonable solution with a 6-step synthetic pathway (CAR Route D, Figure 1). The proposed strategy combines a Sandmeyer reaction with Buchwald amidation. In this proposed route, 5-chloro-4-methyl-2-aminothiazole (**D1**) undergoes Pd-catalyzed arylation to yield 4-methyl-5-methylsulfanyl-2-aminothiazole (**D2**). **D2** is then subjected to a classic Sandmeyer reaction to produce 4-methyl-5-methylsulfanyl-2-bromothiazole (**D3**), which is oxidized to sulfoxide **D4** using hydrogen peroxide as the oxidant and further converted into sulfoximine **D5**. Notably, Synthia suggested three different conditions for this transformation. Sulfoximine **D5** is then coupled with the amide via Buchwald–Hartwig amination to deliver the final API.

Synthia was selected for the subsequent investigations pertaining to the API synthesis due to its ability to deliver more comprehensive reaction pathways, accompanied by detailed reaction conditions and recipes, which include all necessary materials/reactants, solvents, catalysts, etc., from readily available sources. This choice is also dictated by the primary goal of this work, which is the identification of effective and experimentally viable synthetic routes that can be applied directly to the synthesis of API without major modifications or adjustments. More details of the experimental validation of the synthetic routes are discussed below.

## Experimental Validation

The target API, IM-204, underwent systematic retrosynthesis analysis using Synthia software, which employed a scoring function that prioritizes chemoselectivity, regioselectivity, and stereoselectivity while minimizing the use of protecting groups. The CAR offered a 6-step route when no constraints were applied (CAR Route D, Figure 2). This route was experimentally validated in batch, and modifications were performed as necessary to guarantee safety and fit with the experimental facilities. The validation process was benchmarked against the previously reported patents of IM-204.^45,46^

In the first step, Pd-catalyzed C-sulfenylation, the use of methyl mercaptan (MeSH) was deemed impractical and hazardous due to its high flammability and toxicity. This issue could be avoided using sodium methanethiolate (MeSNa). However, as thiolate is a strong nucleophile, it was assumed that a palladium catalyst might not be necessary. Interestingly, upon review of the CAR similarity results, the use of MeSNa is shown on the top examples for this transformation. Consequently, compound **D1** was subjected to *C*-sulfenylation using MeSNa instead of MeSH/Pd, addressing safety concerns. The reaction completion and the formation of sulfide **D2** were confirmed by GC-MS within 2 h of the reaction. The product formation was further confirmed by NMR, and the crude mixture was used to go straight into the next step.

The next CAR-proposed step was a Sandmeyer reaction, an important organic transformation that converts an arylamine to an aryl halide using a Cu(I) halide through a diazonium salt intermediate. Following the recommended conditions,^47^ the crude **D2** mixture from step 1 yielded the brominated compound **D3** with a moderate isolated yield (68%, 2-step).

For the oxidation of sulfide to sulfoxide, it was suggested to use hydrogen peroxide (H_2_O_2_) as the oxidant.^48,49^ When performing the reaction using H_2_O_2_, the desired sulfoxide **D4** was obtained with an excellent isolated yield (91%) after 2 h without any detectable byproducts based on GC-MS. A simple liquid–liquid extraction was also able to provide a pure product. Interestingly, the top results from the CAR similarity suggested using *m*-chloroperbenzoic acid (mCPBA) as the oxidant. However, the oxidation with mCPBA resulted in only a moderate isolated yield (57%).

Hydrogen peroxide is notably more sustainable for organic synthesis than mCPBA and NaIO_4_, as it decomposes into water and oxygen, which helps to avoid generating any toxic waste or byproducts. Nonetheless, its use still poses safety concerns.^50^ The use of mCPBA, on the other hand, is less favorable, as it generates a stoichiometric amount of waste, increasing the PMI of the synthetic process. Moreover, there are safety concerns related to its use in scale-up, given that the pure, dry solid is shock-sensitive and potentially explosive in the condensed phase.^51^

Therefore, we also investigated the use of photooxidation as an alternative to the sulfide oxidation step based on the recent literature.^52^ Photooxidation is a safer and greener alternative for sulfide oxidations as it delivers 100% atom economy, uses air as an oxidant, and demonstrates chemo- and regiocontrol. By performing photooxidation experiments, it was possible to achieve complete conversion of sulfide **D3** to the desired sulfoxide **D4** with an excellent isolated yield (93%) after 4 h of irradiation using riboflavin tetraacetate, a vitamin-based compound, as the photocatalyst. Again, no byproducts were detected either by GC-MS or NMR. Although it has also been previously reported a protocol of self-catalyzed photooxidation for some sulfides, the irradiation of **D3** in the absence of the catalysts did not show any conversion after 24 h of irradiation.

Next, sulfoxide **D4** was fed to the NH-sulfoximine synthesis step. As mentioned earlier, the CAR tool proposed three different conditions for this transformation (see Figure 1, **D4** to **D5**). The first attempt used Eaton’s reagent and sodium azide (NaN_3_) as one of the conditions suggested by Synthia.^53^ However, no product was recovered, and a complex mixture was observed in the NMR spectra. By shifting to the second set of conditions proposed by CAR for the sulfoximine synthesis, using (diacetoxyiodo)benzene (PhI(OAc)_2_) and ammonium carbamate as an ammonia source,^54,55^ it was possible to produce **D5** with a good yield (83%). Notably, this alternative is also safer and uses readily available inexpensive sources.

Compound **D5** was then subjected to Buchwald–Hartwig amidation with **D8** to obtain the final API.^56^ The final reaction was initially attempted without any ligand for the copper catalysts, as Synthia suggested that no ligands can be used. As a result, no product formation was observed, even at high temperatures (reflux) and after long reaction times (72 h). We then performed the reaction in the presence of ligand *N,N*′*-*dimethylethylenediamine (DMEDA) or trans-*N,N*′*-*dimethyl-1,2-cyclohexanediamine (DMCDA) and replaced the copper with Pd/Xantphos, commonly used for Buchwald–Hartwig amidation reactions. However, even under these conditions, the highest yield observed was only 13% (NMR yield) and the total isolated material was 5%. It is worth noting that the reaction was highly sensitive to the quality of the solvents and the purity of the starting materials.

To evaluate the environmental footprint of each of the proposed synthetic pathways, green metrics analysis based on the GreenMotion method was conducted.^57^ This method provides a simple and quantitative gate-to-gate analysis based on the 12 principles of green chemistry and is particularly useful in comparing chemical synthesis. The tool evaluates the environmental impacts of a synthetic process on a scale from 0 to 100. The higher the rating, the safer and less impactful the process. The results (Figure 2, insets) show that CAR route D outperforms the previously reported patent for the synthesis of IM-204, particularly in the “Process” and “Hazard & Toxicity” categories. Those are particularly relevant to process development since the “Process” category takes into account the overall time and energy demand, while the “Hazard & Toxicity” category is related to the safety of the chemicals used.

Although we obtained better results in terms of green metrics, the overall yield of the reaction was slightly lower than that of the reported route.^45,46^ Additionally, for the unconstrained CAR-proposed route D, thiazole **D1** used as a building block was prohibitively expensive (Sigma £720/5g), posing barriers to large-scale applications. Thus, we investigated the effect of a price constraint (SM < $10/g) on the scoring and outputs of the CAR tool.

Surprisingly, the changes involved more than simply extending the route to build the starting material. The combination of the Sandmeyer reaction followed by the *N*-arylation of amides was replaced with amine alkylation followed by classic amide coupling (Figure S1). One interesting observation is that this route is not similar to any of the 10-best scoring routes when no constraints were used.

One notable difference was the change in the NH-sulfoximine step to after the amide bond formation step. This is likely due to the limited examples in the literature of sulfoximine synthesis on substrates containing free amine groups, which typically yield poor results compared to those with protected amines.^58^ Another difference in the newly proposed route is the addition of an *N*-methylamino group through *N*-methylation using toxic formaldehyde and sodium cyanoborohydride. In addition, classic *N*-methylation alternatives, such as using iodomethane, unavoidably would lead to *N*-methylation of the thiazole ring.^59^

These issues could be avoided by designing a route to synthesizing the thiazole heterocycle from scratch. Compound **D1** and its *N*-methylated form (**E4**) can be easily prepared by simple Hantzsch thiazole synthesis followed by chlorination or bromination. Interestingly, again, none of the 10 best CAR-identified routes proposed the synthesis of the heterocycle (see the Associated Content, as the data is on Data Availability Statement). Additionally, designing synthetic routes for the heterocycle from scratch could bring structural versatility due to the limited commercial availability of thiazoles compared to α-haloketone compounds. To explore these alternatives, we modified the constraints for the system to mandate heterocycle synthesis while keeping the price capped at $10/g.

The best CAR-proposed route included a 7-step synthetic pathway (CAR Route E, Figure 2), employing a similar strategy when using the price constraint alone, with amide coupling followed by sulfoximine synthesis to deliver the final product. As expected, with the constraints imposing the heterocycle synthesis, classic Hantzsch thiazole synthesis was proposed with *N*-methylation happening in this step. These changes led to a route with the same number of steps as previously reported but with a 556-fold reduction in the price of the building blocks (see the Supporting Information, Section S1d). The resulting overall route, shown in Figure 2 (CAR route E), was submitted for experimental validation.

Methylaminothiazole **E3** was synthesized via Hantzch synthesis using chloroacetone (**E1**) and *N*-methylthiourea (**E2**) with a good isolated yield (84%). Notably, precipitating the product in water using saturated sodium bicarbonate was sufficient to obtain the pure product. Compound **E3** was then successfully chlorinated using *N*-chlorosuccinimide (NCS), leading to chloromethylaminothiazole **E4** in good yield (71%). The *C-*sulfenylation of **E4** to form **E5** was also achieved in an excellent yield (93%).

The oxidation of **E5** using hydrogen peroxide led to desired sulfoxide **E6** with a good isolated yield (87%). Here, the use of mCPBA as the oxidant also led to a good yield (77%). However, photooxidation experiments showed evidence of the polymerization of the substrate, which may be due to the presence of the secondary amine, as this group typically engages in photoinduced electron transfer. A possible alternative was to perform amide coupling prior to oxidation. Upon reversing the order, the oxidation achieved a low yield (13%) using riboflavin tetraacetate as a photocatalyst, even after 24 h of light irradiation, with a complex mixture of side products observed.

Compound **E9** was synthesized through Suzuki coupling with an excellent yield (95%). Interestingly, the CAR proposed that Suzuki coupling could be performed using carboxylic acid **E8** instead of the methyl ester parent used on the patent, avoiding the need for a hydrolysis step. In addition, the CAR proposed the use of Pd(OAc)_2_ in contrast with the complex phosphine-based catalyst typically used on these coupling reactions.

The next step was dedicated to amide coupling. The use of coupling reagents, such as EDC and HOBt, is the most common approach for amide couplings reported in the literature. Not surprisingly, the CAR tool suggested the use of EDC or DCC as coupling agents, but both failed to produce the desired product, necessitating the screening of a series of coupling agents and various operating conditions (see the Supporting Information). Only when using HATU was it possible to obtain amide **E10** with a yield of 73%.

Compound **E10** was then fed to NH-sulfoximine synthesis using PhI(OAc)_2_ and ammonium carbamate, as previously tested, leading to the production of IM-204 with an excellent step yield (79%) and a 26% overall yield. This result represents more than a 3-fold increase in the overall API synthesis yield compared to the patent that reports the synthesis of the targeted API.^45,46^

Most importantly, the green metrics analysis shows a considerable improvement in the environmental performance. The overall score increased from zero to six, with a significant improvement in the “Reaction” category. This category is particularly relevant due to the incorporation of not only yields and the number of steps but also the atom economy of the process. A slightly better performance in the “Process” category was also observed due to the change from energy-intensive Buchwald–Hartwig coupling (reflux) to amide coupling (conducted at room temperature).

Notably, some of the main penalties in the overall green metrics stem from the solvents used in the total synthesis of IM-204. Another point of optimization for a safer process could be substituting the toxic HATU that is used as the coupling agent in the amide coupling step. Therefore, we considered a human-in-the-loop approach to modify the route and further investigated whether changes in solvents and greener reaction alternatives could be considered. This proposed human-in-the-loop approach is consistent with the most recent findings, which suggest that this approach may deliver better results compared to pure AI-based methods.^60^ The overall changes are shown in Figure 2 (CAR route F).

A straightforward intuitive change was the use of renewable ethanol as a substitute for the CMR (carcinogenic, mutagenic, and reprotoxic chemical) solvent methanol. For Hantzsch synthesis using chloroacetone (**E1**) and *N*-methylthiourea (**E2**), changing from methanol to ethanol did not significantly alter the reaction, and methylaminothiazole **F1** was obtained with a similar isolated yield (83%).

Amide coupling, using typical coupling agents (EDC/DCC/HATU), usually generates a stoichiometric amount of waste, increasing the process mass intensity (PMI) (usually >50). To improve the overall green metrics of the synthetic route, we attempted to use boronic acid catalysis, which typically presents an overall low PMI (<5).^61^ However, no conversion was observed using the most common boronic acid catalysts in the literature (see the Supporting Information). Nevertheless, we successfully changed the solvent for the coupling between **F3** and **F4** to give coupled sulfide **F5**, moving from the CMR solvent dichloromethane (DCM) to renewable 2-methyltetrahydrofuran (2-MeTHF).

The oxidation of **F5** into sulfoxide **F6** was achieved in a 91% yield. Then, **F6** was successfully converted to the sulfoximine product, leading to the final API in the presence of ethanol as the solvent with only a small reduction in the step yield (70%, IM-204d). Nevertheless, the overall yield obtained remained the same (26%). These small changes, however, significantly improved the overall GreenMotion score from 6 to 16 and the Solvent score from 5 to 55.

Finally, the reference provided the CAR tool for the conversion of sulfoxides to sulfoximines using PhI(OAc)_2_, and an ammonia source (Figure 2, **D4** to **D5**) also reported a direct conversion of sulfides to sulfoximines.^58,62^ This method could eliminate one reaction step and the use of oxidants. When the proposed modification was experimentally validated, it was possible to produce the targeted API IM-204 from **F5** with an 83% isolated step yield, increasing the overall yield to 35%, a 4-fold increase compared to the previously reported 8% overall yield.^45,46^ Additionally, the green metrics showed a slight improvement in both the overall and “reaction” scores, which were increased to 18 and 22, respectively.

## Conclusions and Perspectives

Computer-aided retrosynthesis has already demonstrated its capability to generate practicable routes for the synthesis of modestly complex targets. Our methodology illustrates that it is feasible to identify effective, ecofriendly, and experimentally viable total synthetic pathways for even more complex synthetic routes commonly associated with active pharmaceutical ingredients (APIs), as demonstrated with IM-204. Compared to the patented synthetic route of IM-204, the CAR tools delivered a systematic methodology that improved the overall yield from 8% (across 7 steps) to 26% (across 7 steps) while enhancing the greenness overall score from 0 to 6, with significant improvements observed in both the process and reaction scores. By implementation of simple and intuitive modifications, such as substituting methanol with ethanol, it was possible to further enhance the green metrics scores, delivering an overall score of 16. Finally, a human-in-the-loop approach was implemented to help reduce the total number of synthetic steps from 7 to 6, which further increased the overall yield to 35% and enhanced the green metrics to an overall score of 18. Moreover, these changes were accomplished alongside a 556-fold reduction in the total cost of the building blocks. Consequently, this work delivered a comparative study of modern CAR tools and demonstrated their capabilities in the development of more effective, viable, and greener total synthetic routes for complex molecules such as API. The proposed method also contributes to the development of CAR benchmarks emphasizing its role in the development of green-by-design and efficient synthesis of pharmaceuticals and lays the ground for more holistic and robust in silica tools across all development stages.

Combining CAR with other AI technologies, such as yield prediction tools and automated synthesis platforms, could unleash the full potential of CAR in chemical discovery. This integration can enhance data quality and fidelity, reduce resource consumption, and accelerate development timeline.

## Data Availability

Synthia and IBM Rxn reports for the 10 best-ranked routes are available on the Loughborough University Repository and can be accessed at 10.17028/rd.lboro.26792575. Data from CAS ChemPlanner and Reaxys Retrosynthesis are proprietary and are unable to be publicly shared.

## Supporting Information Available

Details of materials and methods for the experimental validation as well as characterization data for the compounds; more details of the CAR results in the text (PDF)
