# Supplementary material for: Computer-Aided Retrosynthesis for Greener and Optimal Total Synthesis of a Helicase-Primase Inhibitor Active Pharmaceutical Ingredient
Source: JACS Au. 2024 Oct 2;4(11):4263–72. doi: 10.1021/jacsau.4c00624 (PMC11709084; doi:10.1021/jacsau.4c00624)
Supplement: Supplementary file 1 — au4c00624_si_001.pdf [file au4c00624_si_001.pdf]

Supporting Information

Computer-Aided Retrosynthesis for Greener and Optimal Total Synthesis of a helicase-primase inhibitor Active Pharmaceutical Ingredient

Rodolfo I. Teixeira,[1*] Michael Andresini[2], Renzo Luisi[2] and Brahim Benyahia[1*]
[1]Department of Chemical Engineering, Loughborough University, Loughborough, LE11 3TU, UK.
[2]Department of Pharmacy - Drug Sciences, University of Bari "A. Moro", Bari, 70125, Italy
**email addresses: r.i.teixeira@lboro.ac.uk, b.benyahia@lboro.ac.uk.*

Keywords: Computer-Aided Retrosynthesis, Computer-Assisted Synthesis Planning, Drug Design, Green-by-Design, Green Chemistry, Pharmaceuticals, Total Synthesis, Helicase-primase Inhibitor.

Contents

S1. Experimental Details S2
- a) General Methods S2
- b) Retrosynthesis Methods S2
- c) Synthesis S3
- d) Total costing of the building blocks..S10

S2. Additional Retrosynthesis data S11

S3. Characterization data for compounds S12

S1. Experimental Details

a) General

Reagents and solvents were purchased from Fisher and used without further purification, unless otherwise described. Sodium thiomethoxide (NaSMe) and palladium acetate ($Pd(OAc)_2$) (99.9%) were purchased from Merck. Phenylboronic acids, *N,N′*-Dicyclohexylcarbodiimide (DCC), *N*-(3-Dimethylaminopropyl)-*N′*-ethylcarbodiimide hydrochloride (EDC-HCl), 1-[Bis(dimethylamino)methylene]-1H-1,2,3-triazolo[4,5-b]pyridinium 3-oxid hexafluorophosphate (HATU), 1-Hydroxybenzotriazole hydrate (HOBt) $Pd(OAc)_2$ 98% and *N,N′*-dimethylethylenediamine (DMEDA) and trans-*N,N′*-dimethyl-1,2-cyclohexanediamine (DMCDA) were purchased from FluoroChem. All solvents used were HPLC grade or higher.

Automatic flash chromatography was performed using a Teledyne ISCO CombiFlash Rf+ system, using UV detection. The methods were developed by using the scout run for defining the gradient. Hexane and ethyl acetate (EtOAc) were used as eluents.

Proton nuclear magnetic resonance (^{1}H NMR), proton-decoupled carbon nuclear magnetic resonance (^{13}C NMR) spectra and 2D NMR spectra (COSY, HSQC, and HMBC) were obtained using a JEOL ECS-400 or ECZ-500 spectrometer. The ^{1}H residual signals of the solvent were used as reference ($CDCl_3$ 7.26 ppm, DMSO-d_6 2.50 ppm), as well as the ^{13}C signals of the solvent ($CDCl_3$ 77.16 ppm, DMSO-d_6 39.52 ppm). Data are represented as follows: chemical shift (δ), integration, multiplicity (s = singlet, d = doublet, t = triplet, q = quartet, dd = doublet doublet, ddd = doublet doublet doublet, dt = doublet triplet, tdt = triplet doublet triplet, m = multiplet), coupling constants (J) is in Hertz (Hz). NMR spectra were processed with MestReNova Software.

GC-MS analyses were carried out using an Agilent GC 6890N coupled with a 5975C VL MSD with triple-axis detector GC-2014 system equipped with a 30 m length, 0.25 mm diameter Supelco SPB-50 column with a 0.25 µm film thickness of fused silica with matrix active group of poly(50% diphenyl / 50% dimethyl siloxane) phase bonded phase – capillary intermediate polarity. Sample injection was performed by an 7693A autosampler using a 1 µL injection volume of sample (100.0 split ratio, split flow: 100 mL/min) with a front inlet temperature of 280°C. The oven was set with a temperature gradient from 60°C to 280°C over 10 min and a holding temperature of 280°C for a further 5 min (15 min total). Helium was as the carrier gas at column flow rate of 1 mL min^{-1} (8.2317 psi). Mass detection (MSD) was used, with a temperature of 250°C.

HRMS were performed by the School of Science (Chemistry Department) of the University of Loughborough using a LTQ XL Orbitrap, using 99.9% Acetonitrile/0.1% formic acid as mobile phase. Injection Volume was 10uL using Flow injection Analysis. FTIR Analysis were performed on an iS50 FT-IR spectrometer using the Attenuated Total Reflection (ATR) module. Samples were measured on the absorption mode using air as background and the baseline was corrected using asymmetric least squares (ALS) function.

b) Retrosynthesis Methods

IM-204 Simplified Molecular Input Line Entry System (SMILES) input:
O=C(N(C1=NC(C)=C(S(C)(=N)=O)S1)C)CC(C=C2)=CC=C2C3=C(F)C=CC(F)=C3

IBM Rxn
IBM RXN was used to deliver Automatic Retrosynthesis on 06/11/2023 with the AI model disconnection-aware-2022-06-24. The Quality tuning was set to High Quality. The input was performed using the SMILES string above. The search was performed under no price limits for the starting materials and under a constraint to the price (20 USD per g/mL). The value of acceptance step (FAP) was kept as 0.65. The tool is based on molecular transformer language models trained on 2.5 million chemical reactions. These models are non-rule based.

Reaxys Retrosynthesis
Reaxys Retrosynthesis was used to predict the synthetic routes on 13/03/2023 using the prediction mode. The published mode was deactivated, since this mode is the regular Reaxys search and only search Reaxys Database for published routes for the target molecule. Notice that the Prediction mode also look at the published routes but show a mixture of results depending on Reaxys ranking score. The SMILES string above was input to the system. The number of output routes was set at 20 routes (the system will show the 20 best predictions). The processing time was extended to guarantee more interactions.

CAS Retrosynthesis
In CAS Retrosynthesis, a synthetic depth of 4 steps (maximum allowed by the CAR tool) was considered under default or common rules and without constraints on the cost of the starting materials. Notice that searches on CAS Retrosynthesis expires after a month and the results can no longer be accessed, and a new search needs to be performed. The ‘evidence’ score was set to zero to allow the CAR tool to explore solutions that has no evidence in their database. The results on CAS ChemPlanner are presented as ‘predicted’ and ‘experimental’. The predict results shows the retrosynthetic results with a depth of 4 steps. That usually leads to incomplete routes for complex molecules. The search can be complemented by ‘experimental’ results where the synthesis of building blocks suggested by the CAS ChemPlanner are searched in the Scifinder database.

Merck Synthia
Synthia was used for automatic retrosynthesis on 18/08/2023 and using the Expert-coded rules and 2000 interactions. Syhtia was also re-run on 17/11/2023 and no differences were observed. Computations were performed using 10000 interactions and led to no differences for the target API. Protecting group use was set to level 3 out of 4 (unlimited). The computations were set to use selective reaction. Building blocks popularity was set to 1, that is the minimum time that the structure appears on the database. For the price constraints, a target price of 10 USD per gram of materials was set. Prices are based on built-in Merck US catalogue. For the chemical constraint route, the tool for building heterocycles from scratch was activated, and all convergent routes were constraint to build the thiazole ring from available building blocks. The output was set to deliver the 50 best routes.

c) Synthesis

4-methyl-5-methylsulfanyl-thiazol-2-ylamine ***(D2)***

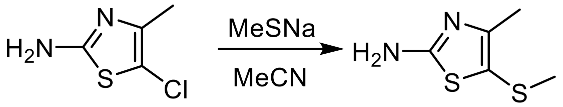

D1 **D2**

To a solution of 5-chloro-4-methylthiazol-2-amine (**D1**, 3.71g, 25 mmol) in 25mL of MeCN was added a solution of NaSMe (2.55g, 36.5 mmol, 1.5 eq.) in 25mL of MeCN. The solution was heated to reflux for 2h. After this time, the mixture was reduced, suspended in water (50 mL), and extracted with EtOAc (2 X 50 mL). The organic layers were dried ($MgSO_4$) and evaporated to yield give a yellow solid (crude **D2**, 3.64g) that was used directly in the next step without further purification.
^{1}H NMR (400 MHz, Acetonitrile-*d*$_3$): δ 2.24 (s, 1H), 2.18 (s, 1H). CG-MS: rt: 9.30 min; mz: 160.0 [M]; 145.0 [M-NH_2]

2-Bromo-4-methyl-5-(methylthio)thiazole ***(D3)***

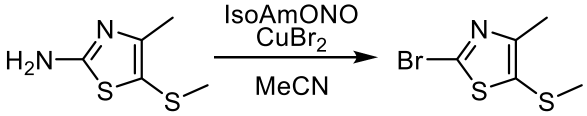

D2 **D3**

Compound **D2** (3.64g, 22 mmol) was dissolved in MeCN (200 mL) and then $CuBr_2$ (7.62g, 1.5 eq.) was added. The reaction flask was sealed with a rubber septum and the suspension was degassed on a stream of N_2 for 15 minutes. The reaction flask was then transferred to an ice salt bath. The solution was stirred for another 15 min while still under a stream of nitrogen. Then isoamyl nitrite (6 mL, 2 eq.) was then added dropwise using a syringe pump (0.5 mL min^{-1}). The reaction was allowed to stir for 1h under ice. The reaction mixture was allowed to warm to room temperature and left stirring for a further 3h. The solution was then poured into HCl ~1M (400 mL) and stirred for 5 min. Then EtOAc was added (250 mL). The layers were separated, and the Organic layer was washed one more time with HCl 1M. Then, the combined aqueous layers were extracted with EtOAc (2 x 150 mL). The organic layers were combined, dried over Na_2SO_4, filtered over a pad of silica, and the solvent removed on a rotavaporator. The residue was purified by Flash Chromatography using a Teledyne. NextGen flash system. Product obtained as a dark yellow oil (**D3**, 3.79g, 17 mmol, 68%, 2-step yield).
^{1}H NMR (400 MHz, $CDCl_3$-*d*) δ 2.44 (s, 1H), 2.36 (s, 1H). CG-MS: 8.13 min; mz: 224.8, 222.9 [M].

2-bromo-4-methyl-5-(methylsulfinyl)thiazole ***(D4)***

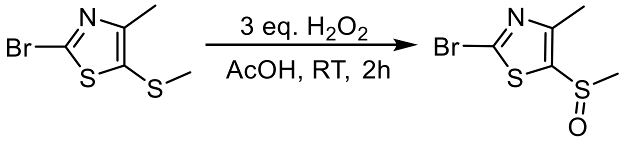

D3 **D4**

a) To a solution of sulfide **D3** (1.05g, 4.7 mmol) in glacial acetic acid (4 mL) was slowly added hydrogen peroxide (30% w/w, 1.8 mL, 17.6 mmol). The reaction mixture was then stirred at room temperature for 2h and the completing was monitored by CG-MS. The resulting solution was neutralized with aqueous NaOH (4 M, ~15 mL) and the product was extracted with EtOAc (3 x 10 mL). The organic layer was dried over anhydrous Na_2SO_4 and then concentrated under reduced pressure to yield a dark yellow solid (**D4**, 1.02g, 4.3 mmol, 91%).
^{1}H NMR (400 MHz, Chloroform-*d*) δ 2.91 (s, 1H), 2.52 (s, 1H). ^{13}C NMR (126 MHz, Chloroform-*d*) δ 157.99, 141.13, 134.74, 45.60, 16.29. GC-MS: rt: 10.60 min; mz: 238.9, 240.8 [M]; 223.9, 225.9 [M-Me].

b) Oxidation using *m*-CPBA. To a solution of **D3** (0.1929g, 0.86 mmol) in dichloromethane (10 mL) was added m-chloroperbenzoic acid (0.33g, 70%, 1.33 mmol) in small batches. The reaction was allowed to stir for around 30 min. Ethyl acetate (40 mL) was added to the reaction mixture, which was then washed with saturated aqueous sodium bicarbonate. The aqueous layer was further extracted with EtOAc (2 x 20 mL). Finally, the combined organic layers were extracted with water and brine, dried over magnesium sulphate and brown solid (**D4**, 0.1178g, 0.49 mmol).

c) Photooxidation. In a glass vial containing **D3** (0.2103g, 0.94 mmol) and tetra-*O*-acetylriboflavin (0.0027g, 0.5 mol%) was added 2 mL of methanol. The reaction mixture was transferred to a 3D printed parallel reactor and was left stirring under Blue LED (Kessil PR 160L, 427 nm) irradiation for 4h. During the irradiation a flow of air was bubbled trough the solution to guarantee enough oxygen was present. The completion of the reaction was verified by GC-MS. The reaction mixture was diluted with EtOAc and filtered over silica to afford the desired product (**D4**, 0.2098g, 0.87 mmol, 93%).

2-bromo-4-methyl-5-(methylsulfonimidoyl)thiazole ***(D5)***

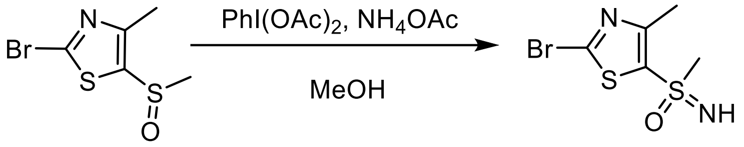

D4 **D5**

The sulfoxide **D4** (0.4272g, 1.8 mmol), (diacetoxyiodo)benzene (1.4612g, 4.5 mmol, 2.5 eq.) and ammonium carbamate (0.3038g, 3.9 mmol, 2.2 eq.) were added to a flask containing a stirrer bar. MeOH (3 mL) was added, and the reaction was stirred at 25°C for 3h. The solvent of the reaction mixture was evaporated using rotatory evaporator. The crude product was purified using flash chromatography (Teledyne CombiFlash NextGen+, gradient from 1:9 hexane:EtOAc to pure EtOAc) to afford the desired product as an off-white solid. (**D5**, 0.3809g, 1.5 mmol, 83%)
^{1}H NMR (400 MHz, Chloroform-*d*) δ 3.21 (s, 3H), 2.67 (s, 3H). ^{13}C NMR (101 MHz, Chloroform-*d*) δ 156.74, 140.39, 138.63, 46.61, 16.04. GC-MS: rt: 10.98 min; mz: 253.9, 255.9 [M]. HRMS - Calc: 253.9183 [M]; 254.9256 [M+H$^+$]. Found: 254.9248, 256.9224 [M+H$^+$], error: 3.1 ppm.

2-(2',5'-difluoro-[1,1'-biphenyl]-4-yl)-N-methylethanamide ***(D8)***

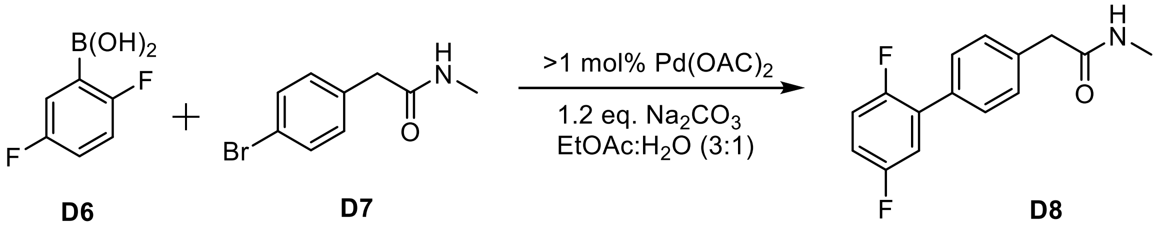

D6 **D7** **D8**

To a flask containing 2-(4-bromophenyl)-N-methylethanamide (**D7**, 1.1236g, 4.9 mmol) was added 2,5-Difluorophenylboronic acid (**D6**, 0.9657g, 6.1 mmol, 1.25 eq.), palladium acetate (0.0161g, 1 mol %), sodium carbonate (0.6402g), water (5 mL) and ethyl acetate (10 mL). The reaction was heated to 65 ^{o}C and stirred for 18 hours. The reaction was allowed to cool to room temperature and the mixture was diluted with water (10 mL), acidified with hydrochloric acid to produce a precipitate, and extracted with EtOAc (3 × 15 mL). The combined organic layers were dried over $MgSO_4$, and the solvent removed on rotatory evaporator to obtain a white solid (**D8**, 1.1779g, 4.5 mmol, 92%)
^{1}H NMR (400 MHz, Chloroform-*d*) δ 7.53 (dd, *J* = 8.3, 1.7 Hz, 2H), 7.35 (d, *J* = 8.2 Hz, 2H), 7.18 – 7.06 (m, 2H), 7.06 – 6.95 (m, 1H), 5.41 (s, 1H), 3.62 (s, 2H), 2.80 (d, *J* = 4.8 Hz, 3H).
^{19}F NMR (376 MHz, Chloroform-*d*) δ -119.06, -124.29.
^{13}C NMR (101 MHz, Chloroform-*d*) δ 171.02, 158.57 (d), 155.49 (d), 134.76, 133.63, 130.26 (dd), 129.49, 129.22 (d), 116.97 (dd), 116.50 (dd), 115.09 (dd), 43.14, 26.29.
HRMS - Calc: 261.0965 [M]; 262.1038 [M+H+]. Found: 262.1028 [M+H+], error 3.8 ppm.

*2-(2',5'-difluoro-[1,1'-biphenyl]-4-yl)-N-methyl-N-(4-methyl-5-(S-methylsulfonimidoyl)thiazol-2-yl)acetamide (**IM-204a**)*

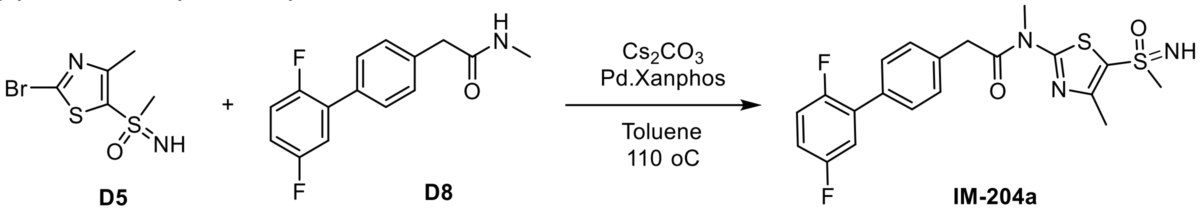

D5 **D8** **IM-204a**

To a dried, sealed tube under a nitrogen atmosphere was added **D5** (0.1003g, 0.39 mmol), **D8** (0.0981g, 0.37 mmol), >99% grade $Pd(OAc)_2$ (0.0084g, 10 mol%, 0.037 mmol), 98%+ grade xantphos (0.0416g, 20 mol%, 0.072 mmol) and Cs_2CO_3 (0.1844 g, 0.56 mmol). The tube was sealed with a Teflon cap with a PTFE seal. Then deoxygenated dry toluene (4 mL) was added under a stream of nitrogen. The reaction mixture was stirred at 100°C for 72h. The mixture was filtered through celite, and the solvent removed on rotatory evaporator. The crude was purified by flash chromatography (Teledyne CombiFlash NextGen+, gradient starting with pure hexane, then from hexane:EtOAc 9:1 to pure EtOAc) to afford the product was a white solid (**IM204a**, 8mg, 0.02 mmol, 5% yield (13% by NMR)).
^{1}H NMR (400 MHz, Chloroform-*d*) δ 7.53 (dd, *J* = 8.3, 1.7 Hz, 2H), 7.35 (d, *J* = 8.6 Hz, 2H), 7.17 – 7.05 (m, 2H), 7.05 – 6.94 (m, 1H), 4.07 (s, 2H), 3.73 (s, 3H), 3.19 (s, 3H), 3.10 (s, 1H), 2.61 (s, 3H).

Attempts to use crude **D5** led to no product. Use of lower purity $Pd(OAc)_2$/Xantphos and non-dry toluene also led to no product detection. Attempts to use copper iodide and copper bromide as catalysts in combination with *N,N'*-dimethylethylenediamine (DMEDA) and trans-*N,N'*-dimethyl-1,2-cyclohexanediamine (DMCDA) as ligands also failed to give the desired product and the starting materials were recovered from the mixture.

*2-methylamino-4-methylthiazole **(E3/F1)***

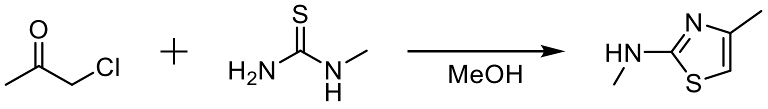

E1 **E2** **E3**

To a solution of chloroacetone (**E1**, 97.7 mmol, 9.04g) in 100 mL of MeOH were added 1-methylthiourea (**E2**, 110 mmol, 9.9g 1.13 eq.) and 3 mL of pyridine, and the mixture was stirred at room temperature for 4 h. The mixture was concentrated, and the precipitate was washed with saturated $NaHCO_3$ solution (*ca.* 60 mL), filtered, and dried in desiccator overnight to afford the title compound as a white solid. The product was spectroscopy pure and could be used on the next step, but a significant water signal could still be observed by NMR. In order to obtain dry product for characterization purposes, the wet product was then dissolved in toluene (*ca.* 50 mL), and the solvent was removed in a rotavap to azeotropic removal of water. The procedure was repeated 4 times. The residue was then suspended in 10mL EtOAc and evaporated and dried in a rotavap (4h at 20 mmbar at 40°C) to give an off white solid (**E3**, 10.53g, 82 mmol, 84% yield). **F1** was prepared in the same way using EtOH as solvent (**F1**, 5.79g, 45.2 mmol, 83% yield; from 5.04g of chloroacetone) .
^{1}H NMR (400 MHz, Chloroform-*d*) δ 6.05 (q, *J* = 1.1 Hz, 1H), 5.80 (s, 1H), 2.94 (s, 3H), 2.23 (d, *J* = 1.1 Hz, 3H). GC-MS: rt: 6.81 min; mz: 128.0, 127.0 [M]; 99.0, 100.0 [M-NMe]. HRMS - Calc: 128.0408 [M]; 129.0481 [M+H+]. Found: 129.0479 [M+H], error 1.5 ppm:

5-chloro-N,4-dimethyl-1,3-thiazol-2-amine ***(E4/F2)***

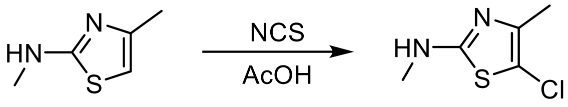

E3 **E4**

A mixture of 2-methylamino-4-methylthiazole (**E3**, 4.21g, 32.8 mmol, 1 eq.) was dissolved in about 40mL of Acetic acid. The mixture was put to stir in an oil bath at a starting temperature of about 65°C. The N-chlorosuccinimide (NCS, 5.41g, 40.5 mmol, 1.25 eq.) was added in a single batch. The reaction was poured into ice. 150 mL of NaOH 4M was carefully added to the solution, not allowing the temperature to warm up higher than 5ºC. The reaction was then fully neutralised by adding a saturated carbonated solution. The crude was extracted 3x using a mixture of Et_2O:EtOAc (1:1; ~200 mL total). The organic layers were combined, washed with brine, and dried using $MgSO_4$. the organic layer was then filtered over silica and washed with ~200 mL Et_2O:EtOAc (1:1). The combined fractions were evaporated to give a yellow solid (**E4**, 3.92g, 23.2 mmol, 71%). The reaction was repeated to obtain **F2** from **F1**, and similar results were obtained.
^{1}H NMR (400 MHz, Chloroform-*d*) δ 4.46 (s, 1H), 2.91 (s, 3H), 2.16 (s, 3H). GC-MS: 7.98 min; mz: 162.0, 164.0 [M]; 133.9, 135.9 [M-NMe]. ^{13}C NMR (126 MHz, Chloroform-*d*) δ 167.12, 144.24, 101.90, 31.89, 14.39. HRMS - Calc: 162.0018 [M]; 163.0091 [M+H$^+$]. Found: 163.0086 [M+H$^+$], error 3.1ppm.

N,4-dimethyl-5-(methylthio)thiazol-2-amine ***(E5/F3)***

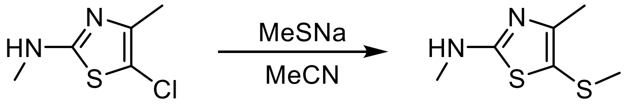

E4 **E5**

To a solution of **E4** (4.10g, 25.2 mmol) in 25 mL of MeOH was added a solution of NaSMe (3.12 g, 44.5 mmol, 1.8 eq.) in 25 mL of MeOH slowly. The solution was heated to reflux for 2h. After this time, the mixture was reduced, suspended in water, and extracted with EtOAc (3 X 50 mL). The organic layers were dried ($MgSO_4$) and evaporated to yield give a yellow solid (**E5**, 4.08g, 23.4 mmol, 93%). The reaction was repeated to obtain **F3** from **F2**, and similar results were obtained.
^{1}H NMR (400 MHz, Chloroform-*d*) δ 2.93 (s, 1H), 2.29 (s, 1H), 2.27 (s, 1H). ^{13}C NMR (126 MHz, Chloroform-*d*) δ 171.08, 156.71, 114.28, 32.02, 22.57, 16.10. GC-MS: rt: 9.51 min; mz: 174.0 [M]; 159.0 [M-Me]. HRMS - Calc: 174.0285 [M]; 175.0358 [M+H$^+$]. Found: 175.0353 [M+H$^+$], error 2.8ppm.

N,4-dimethyl-5-(methylsulfinyl)thiazol-2-amine ***(E6)***

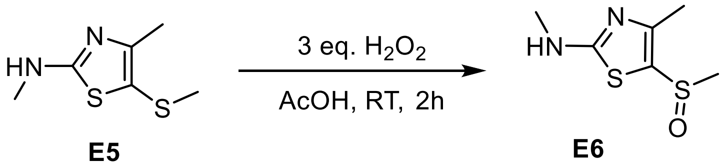

E5 **E6**

Reaction was performed as described to compound **D4**. Used **E5** (1.75g, 10mmol) to afford **E6** (1.66g, 8.7 mmol, 87%). ^{1}H NMR (400 MHz, Chloroform-*d*) δ 6.39 (s, 1H), 2.98 (s, 3H), 2.87 (s, 3H), 2.36 (s, 3H). ^{13}C NMR (126 MHz, Chloroform-*d*) δ 173.49, 155.70, 120.37, 43.05, 32.04, 16.32. GC-MS: rt: 13.45 min; mz: 190.0 [M]; 175.0 [M-Me].

2-(2,5'-difluoro-[1,1'-biphenyl]-4-yl)acetic acid ***(E9/F4)***

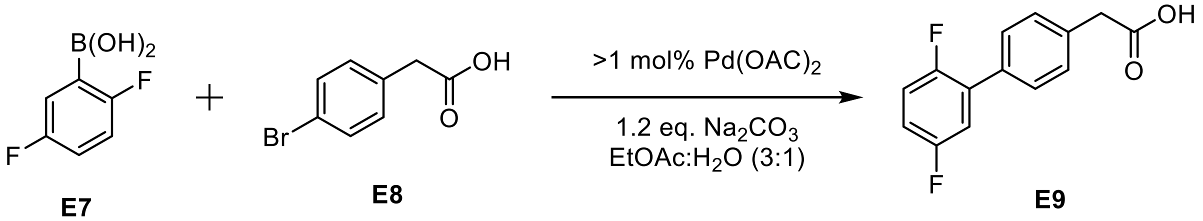

E7 **E8** **E9**

To 4-bromophenylacetic acid (**E8**, 10.48g, 48.7 mmol) was added 2,5-Difluorophenylboronic acid (**E7**, 10.27g, 65mmol, 1.3 eq.*), palladium acetate (0.1613g, 1 mol%), sodium carbonate (7.02g), water (50 mL) and ethyl acetate (150 mL). The reaction was heated to 65°C and stirred for 18 hours. The reaction was allowed to cool to room temperature and the mixture was diluted with water (100 mL), acidified with hydrochloric acid to produce a precipitate, and extracted with EtOAc (3 × 100 mL). The combined organic layers were dried over $MgSO_4$, and the solvent removed on rotatory evaporator to afford a white solid (**E9**, 11.49g, 46.2 mmol, 95%). Similar results were obtained for **F4**.
*1.0, 1.1 and 1.2 equivalents of the boronic acid were tested, but the full conversion of 4-bromophenylacetic acid was only observed when using 1.3 equivalents of 2,5-Difluorophenylboronic acid.
^{1}H NMR (400 MHz, Chloroform-*d*) δ 7.51 (dd, J = 8.3, 1.8 Hz, 2H), 7.38 (d, J = 8.2 Hz, 2H), 7.18 – 7.05 (m, 2H), 7.04 – 6.94 (m, 1H), 3.72 (s, 2H). ^{19}F NMR (376 MHz, Chloroform-*d*) δ -119.14, -124.12. ^{13}C NMR (101 MHz, Chloroform-*d*) δ 176.84, 158.60 (d), 155.56 (d), 133.74, 133.09, 129.68 (dd), 129.45, 128.97 (d), 116.98 (dd), 116.61 (dd), 115.07 (dd), 40.45.

2-(2',5'-difluoro-[1,1'-biphenyl]-4-yl)-N-methyl-N-(4-methyl-5-(methylsulfinyl)thiazol-2-yl)acetamide ***(E10)***

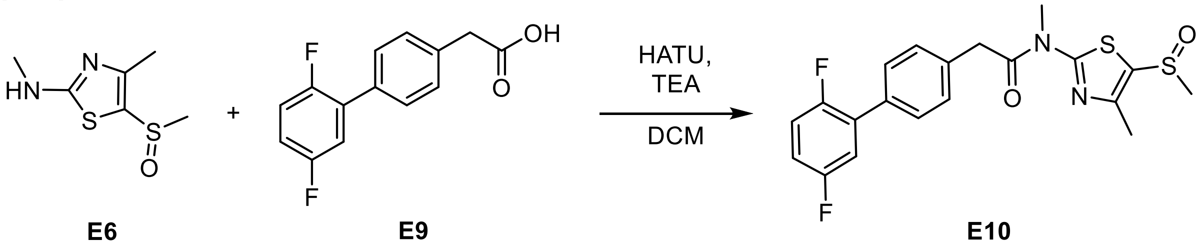

E6 **E9** **E10**

E9 (1.0108g, 4.1 mmol), HATU (2.3307g, 6 mmol) and DIPEA (1ml, 6 mmol) were dissolved in 10 mL of DCM at 0°C. Then **E6** (0.8347g, 4.4 mmol) in 10 ml DCM was added dropwise using a syringe pump (1 mL min^{-1}). The solution was allowed to warm to room temperature and let to stir overnight. The reaction mixture was extracted with water (2 x 20 mL). the organic layer was dried over Mg_2SO_4 and the solvent evaporated. The residue was purified by flash chromatography (Teledyne CombiFlash NextGen+, gradient starting with pure hexane, then from hexane:EtOAc 9:1 to 1:1) to afford the product was a white solid (**E10**, 1.25g, 3 mmol, 73%).
^{1}H NMR (500 MHz, Chloroform-d) δ 7.52 (d, J = 8.0 Hz, 2H), 7.34 (d, J = 8.0 Hz, 2H), 7.15 – 7.05 (m, 2H), 7.02 – 6.94 (m, 1H), 4.07 (s, 2H), 3.73 (s, 3H), 2.93 (s, 3H), 2.49 (s, 3H).
^{19}F NMR (471 MHz, Chloroform-*d*) δ -118.72, -123.86.
^{13}C NMR (101 MHz, Chloroform-*d*) δ 170.97 (s), 161.72 (s,), 158.67 (d), 155.78 (d), 151.55 (s), 134.18 (s), 132.80 (s), 129.74 (dd), 129.55 (d), 129.43 (s), 129.36 (s), 117.32 (dd), 116.84 (dd), 115.47 (dd), 42.61 (s), 41.70 (s), 35.10 (s, C-17), 16.23 (s).
HRMS - Calc: 420.0778 [M]; 421.0851 [M+H]. Found: 421.0849 [M+H], error 0.5 ppm.

The coupling using DCC/DMAP, EDC-HCl, and HOBt were attempted, but no product was recovery from the reaction mixture.
The use of boronic acid as catalysts for the amidation was also attempted, but again no product was detected. The boronic acid catalysts tested were: 3,4,5-Trifluorophenylboronic acid, 2,4-Bis(trifluoromethyl)phenylboronic acid, 2-Nitrophenylboronic acid, 1-Thianthrenylboronic acid and 5-Methoxy-2-iodophenylboronic acid.

*2-(2',5'-difluoro-[1,1'-biphenyl]-4-yl)-N-methyl-N-(4-methyl-5-(S-methylsulfonimidoyl)thiazol-2-yl)acetamide (**IM-204b**)*

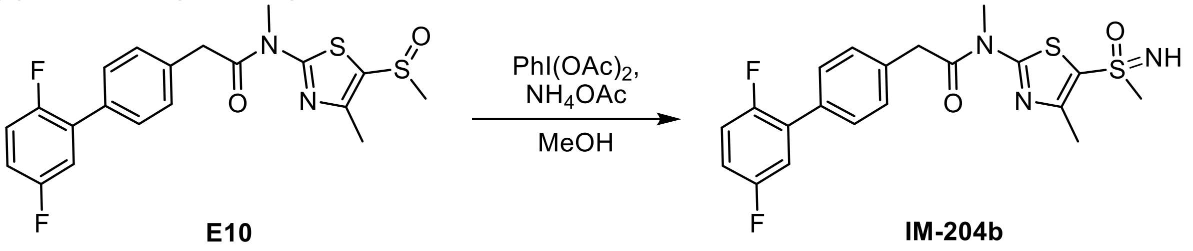

Reaction was performed as described to compound **D5** using **E10** (0.2044g, 0.49 mmol), (diacetoxyiodo)benzene (0.4065g, 1.26 mmol), ammonium acetate (0.098g, 1.27 mmol) and MeOH (1 mL). The residue was purified by flash chromatography (Teledyne CombiFlash NextGen+, gradient starting with pure hexane, then from hexane:EtOAc 9:1 to pure EtOAc) to afford the product was a white solid (**IM204b**, 0.1686g, 0.39 mmol, 79%).

^{1}H NMR (400 MHz, Chloroform-*d*) δ 7.53 (dd, *J* = 8.3, 1.7 Hz, 2H), 7.35 (d, *J* = 8.6 Hz, 2H), 7.17 – 7.05 (m, 2H), 7.05 – 6.94 (m, 1H), 4.07 (s, 2H), 3.73 (s, 3H), 3.19 (s, 3H), 3.10 (s, 1H), 2.61 (s, 3H).

^{19}F NMR (376 MHz, Chloroform-*d*) δ -119.04, -124.22.

^{13}C NMR (101 MHz, Chloroform-*d*) δ 170.93 (s, C-14), 160.82 (s, C-18), 158.60 (d, C-3), 156.15 (d, C-6), 152.09 (s, C-21), 134.25 (s, C-7), 132.69 (s, C-10), 129.72 (dd, C-5), 129.58 (d, C-8/12), 129.34 (s, C-9/11), 128.67 (s, C-20), 117.31 (dd, C-2), 116.85 (dd, C-4), 115.50 (dd, C-1), 47.04 (s, C-25), 41.65 (s, C-13), 34.71 (s, C-17), 16.33 (s, C-23). Signals were confirmed by COSY, HSQC and HMBC (see below).

HRMS - Calc: 435.0887 [M]; 436.0960 [M+H]. Found: 436.0933 [M+H], error 6.1 ppm.

*2-(2',5'-difluoro-[1,1'-biphenyl]-4-yl)-N-methyl-N-(4-methyl-5-(methylthio)thiazol-2-yl)acetamide **(F5)***

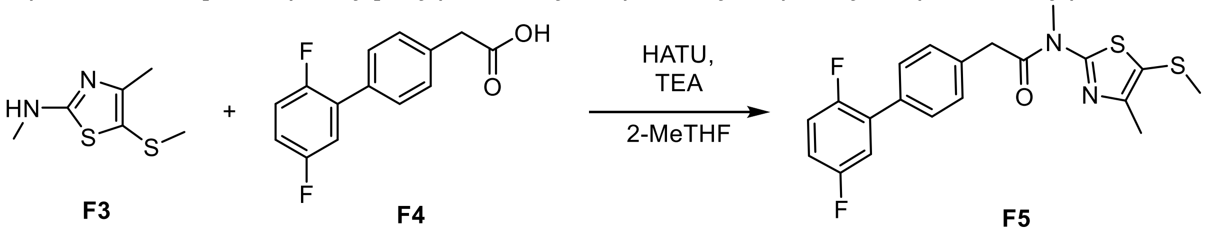

Reaction was performed as described to compound **E10** using **F4** (2.01g, 8.1 mmol), HATU (4.86g, 12.8 mmol), DIPEA (2.1 mL, 12 mmol) in 2-MeTHF (30 mL) and **F3** (1.52g, 8.7 mmol) in 2-MeTHF (30 mL) was added dropwise. Product was purified using Flash chromatography to obtain a white solid product (**F5**, 2.62g, 6.5 mmol, 80%). Alternatively, after the extracting, the crude product can be recrystallized on Et_2O, but with a crystallization yield of 57%. ^{1}H NMR (400 MHz, Chloroform-*d*) δ 7.51 (dd, *J* = 8.2, 1.6 Hz, 1H), 7.35 (d, *J* = 8.3 Hz, 1H), 7.15 – 7.04 (m, 1H), 7.03 – 6.93 (m, 0H), 4.03 (s, 1H), 3.69 (s, 1H), 2.39 (s, 1H), 2.32 (s, 1H). ^{19}F NMR (376 MHz, Chloroform-*d*) δ -119.15, -124.19. ^{13}C NMR (101 MHz, Chloroform-*d*) δ 170.97 (s), 170.36 (s), 159.07 (s), 158.86 (d), 155.78 (d), 150.38 (s), 133.64 (d), 129.85 (dd), 129.47 (s), 119.79 (s), 117.30 (dd), 116.86 (dd), 115.40 (dd), 41.77 (s), 34.71 (s), 21.71 (s, C-17), 15.51 (s).

HRMS - Calc: 404.0829 [M]; 405.0901 [M+H]. Found: 405.0885 [M+H], error 3.9 ppm.

*2-(2',5'-difluoro-[1,1'-biphenyl]-4-yl)-N-methyl-N-(4-methyl-5-(methylsulfinyl)thiazol-2-yl)acetamide **(F6)***

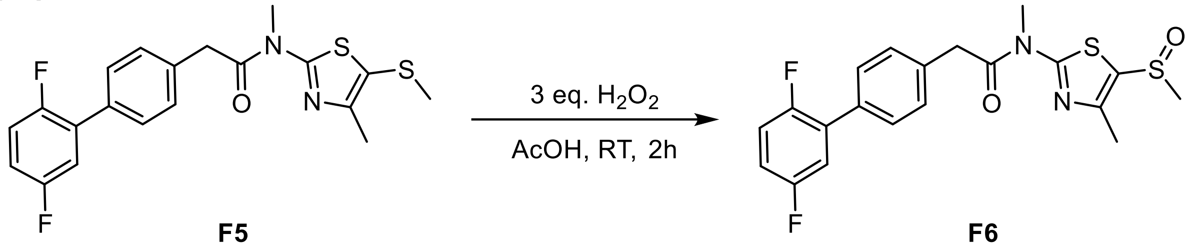

F5 **F6**

Reaction was performed as described to compound **D4** using **F5** (0.448g, 1.1 mmol) to afford **F6** (0.4228g, 1.0 mmol, 91%). ^{1}H NMR (400 MHz, Chloroform-*d*) δ 7.52 (dd, *J* = 8.3, 1.8 Hz, 2H), 7.35 (d, *J* = 8.2 Hz, 2H), 7.16 – 7.05 (m, 2H), 7.03 – 6.94 (m, 1H), 4.03 (d, *J* = 2.0 Hz, 2H), 3.70 (s, 2H), 2.40 (s, 2H), 2.32 (s, 2H).

*2-(2',5'-difluoro-[1,1'-biphenyl]-4-yl)-N-methyl-N-(4-methyl-5-(S-methylsulfonimidoyl)thiazol-2-yl)acetamide (**IM-204c**)*

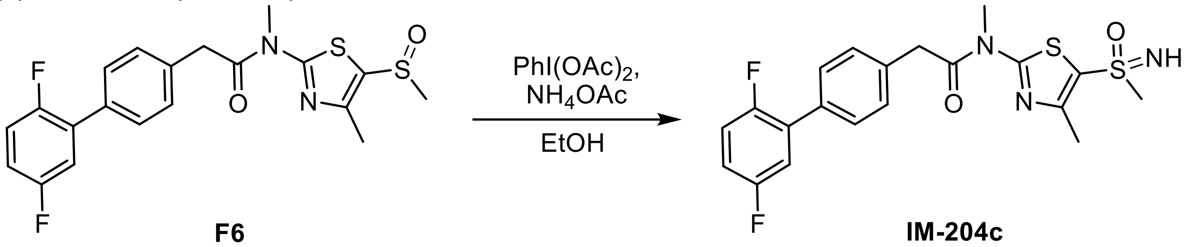

F6 **IM-204c**

Reaction was performed as described to **IM-204b** but using **F6** (0.1447g, 0.34 mmol), (diacetoxyiodo)benzene (0.2920g, 0.91 mmol), ammonium acetate (0.0631g, 0.82 mmol) and EtOH (2 mL). The crude was purified by flash chromatography (Teledyne CombiFlash NextGen+, gradient starting with pure hexane, then from hexane:EtOAc 9:1 to pure EtOAc) to afford the **IM-204c** was a white solid (0.1036g, 0.24 mmol, 70%).
^{1}H NMR (400 MHz, Chloroform-*d*) δ 7.53 (dd, *J* = 8.3, 1.7 Hz, 2H), 7.35 (d, *J* = 8.6 Hz, 2H), 7.17 – 7.05 (m, 2H), 7.05 – 6.94 (m, 1H), 4.07 (s, 2H), 3.73 (s, 3H), 3.19 (s, 3H), 3.10 (s, 1H), 2.61 (s, 3H).

*2-(2',5'-difluoro-[1,1'-biphenyl]-4-yl)-N-methyl-N-(4-methyl-5-(S-methylsulfonimidoyl)thiazol-2-yl)acetamide (**IM-204d**)*

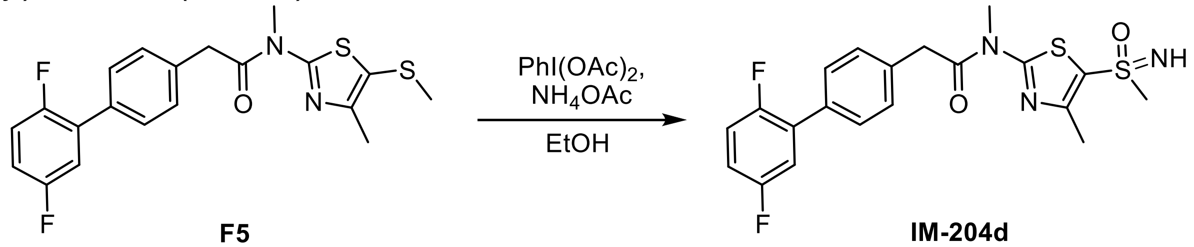

F5 **IM-204d**

Reaction was performed as described to **IM-204b** but using **F5** (0.2044g, 0.51 mmol), (diacetoxyiodo)benzene (0.4065g, 1.26 mmol), ammonium acetate (0.0981g, 1.27 mmol) and EtOH (2mL). The crude was purified by flash chromatography (Teledyne CombiFlash NextGen+, gradient starting with pure hexane, then from hexane:EtOAc 9:1 to pure EtOAc) to ^{1}H NMR (400 MHz, Chloroform-*d*) δ 7.53 (dd, *J* = 8.3, 1.7 Hz, 2H), 7.35 (d, *J* = 8.6 Hz, 2H), 7.17 – 7.05 (m, 2H), 7.05 – 6.94 (m, 1H), 4.07 (s, 2H), 3.73 (s, 3H), 3.19 (s, 3H), 3.10 (s, 1H), 2.61 (s, 3H).

d) Total costing of the building blocks

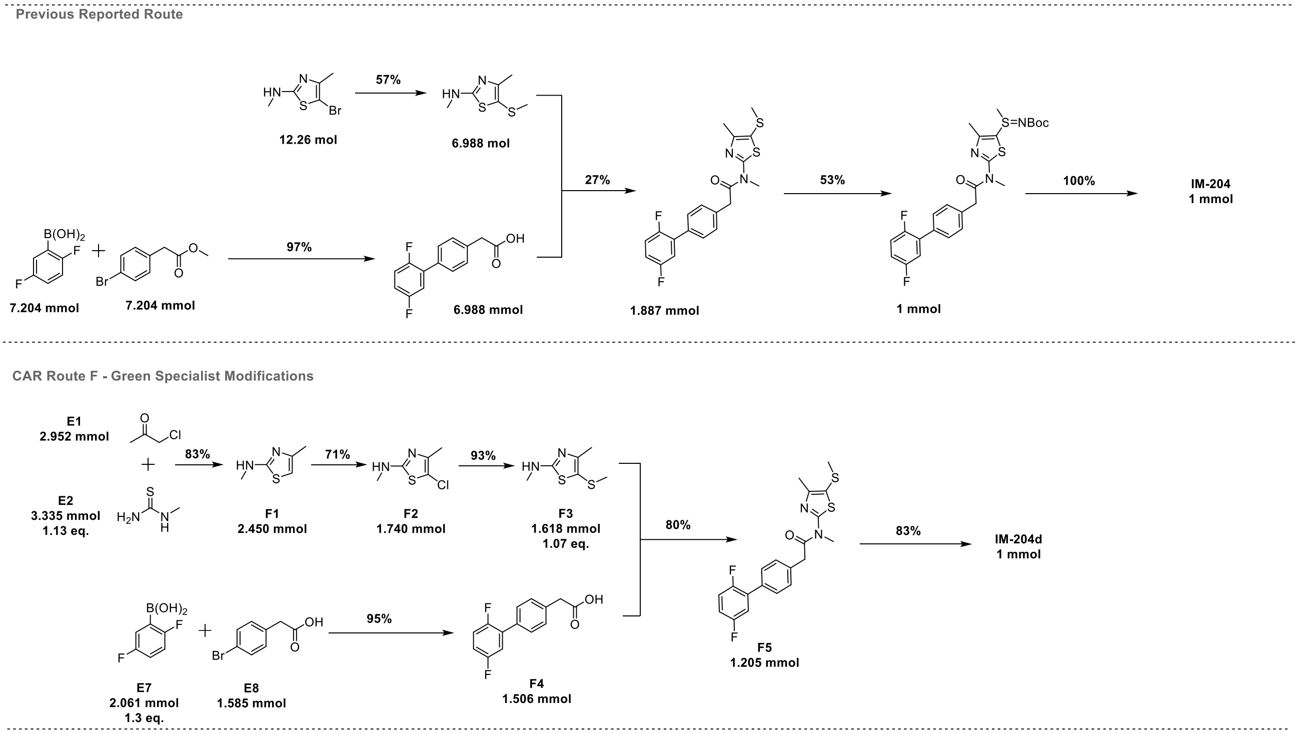

Scheme S1. Calculation of mmol required to obtain 1 mmol of IM-204.

Table S1. Costing of building blocks considered. Search of done on 21 June 2024 on reliable available suppliers in the UK.

	Compound	CAS	Price	Amount	Supplier	MW	Price/mmol
A	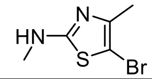	878890-10-9	£1440.99	10 g	Sigma-Aldrich	207.09	£29.84
B	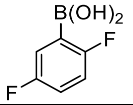	193353-34-3	£115.00	100 g	Fluorochem	157.91	£0.18
C	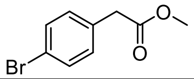	41841-16-1	£105.00	500 g	Fluorochem	229.073	£0.05
D	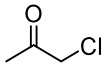	78-95-5	£78.00	500 g	Sigma-Aldrich	92.52	£0.02
E	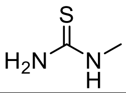	598-52-7	£57.50	100 g	Sigma-Aldrich	90.15	£0.05
F	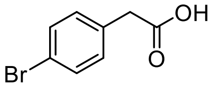	1878-68-8	£18.00	100 g	Fluorochem	215.046	£0.04

Costing of the building blocks for the reported patent of IM-204. Nuilding Block considered as reported on the patent
Price = 12.26×A + 7.204×B 7.204×C = £367.49 per mmol of IM-204

This Work – CAR Route, using the pathway to IM-204d.
Price = 2.952×D + 3.335×E + 1.585×F + 2.061×C = £0.66 per mmol of IM-204

S2. Additional Retrosynthesis data

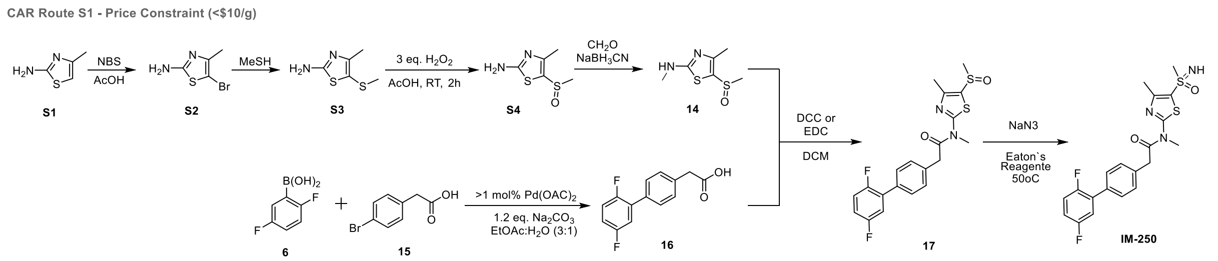

Figure S1. Best scoring route proposed by Synthia using a constraint that restrict the price of building block to less than $10.00 per gram of material.

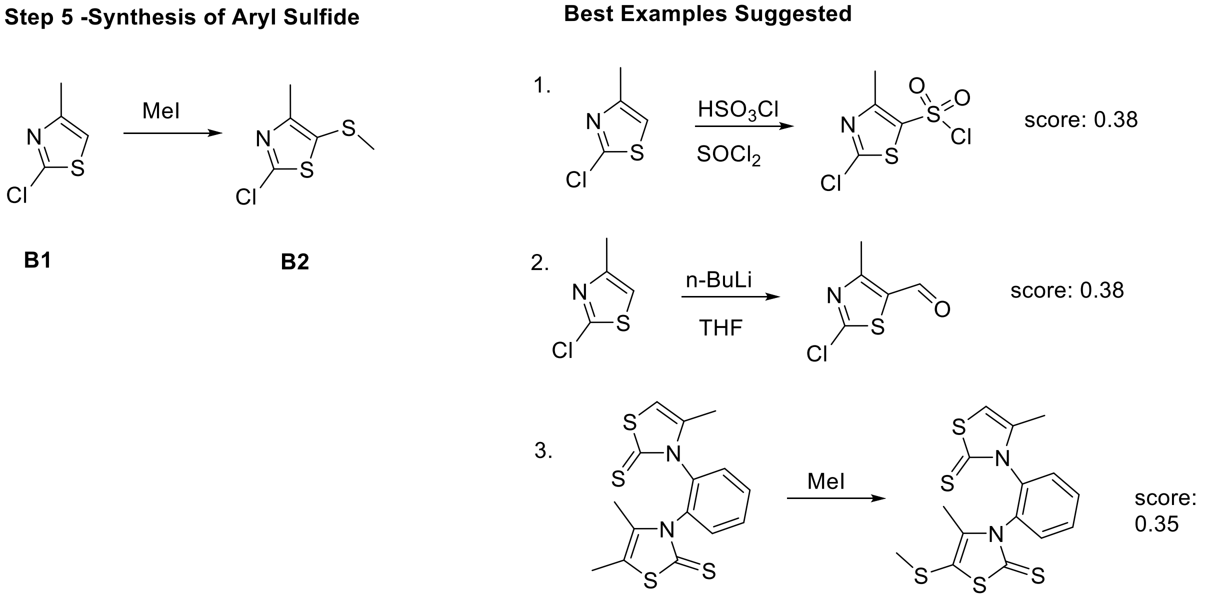

Figure S2. Best ranked examples listed by Reaxys Retrosynthesis for the Synthesis of Aryl sulfide B2.

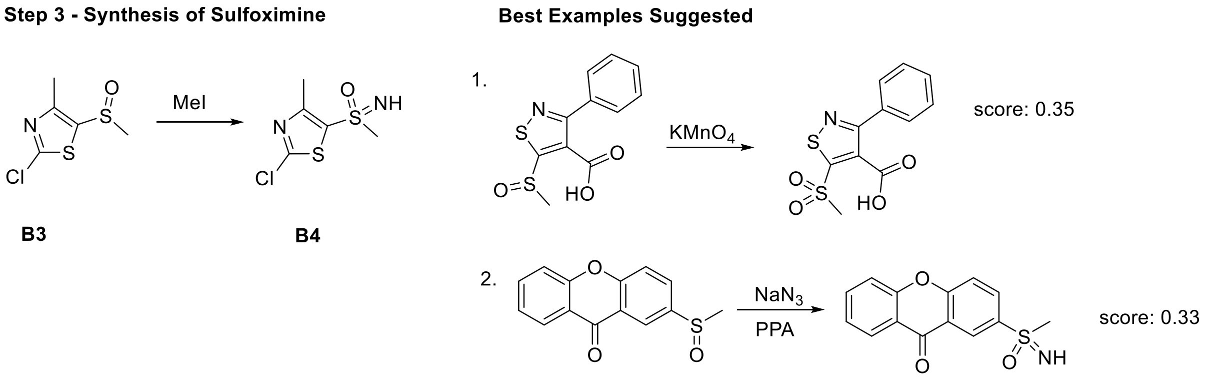

Figure S3. Best ranked examples listed by Reaxys Retrosynthesis for the Synthesis Sulfoximine B4.

S3. Characterization data for compounds

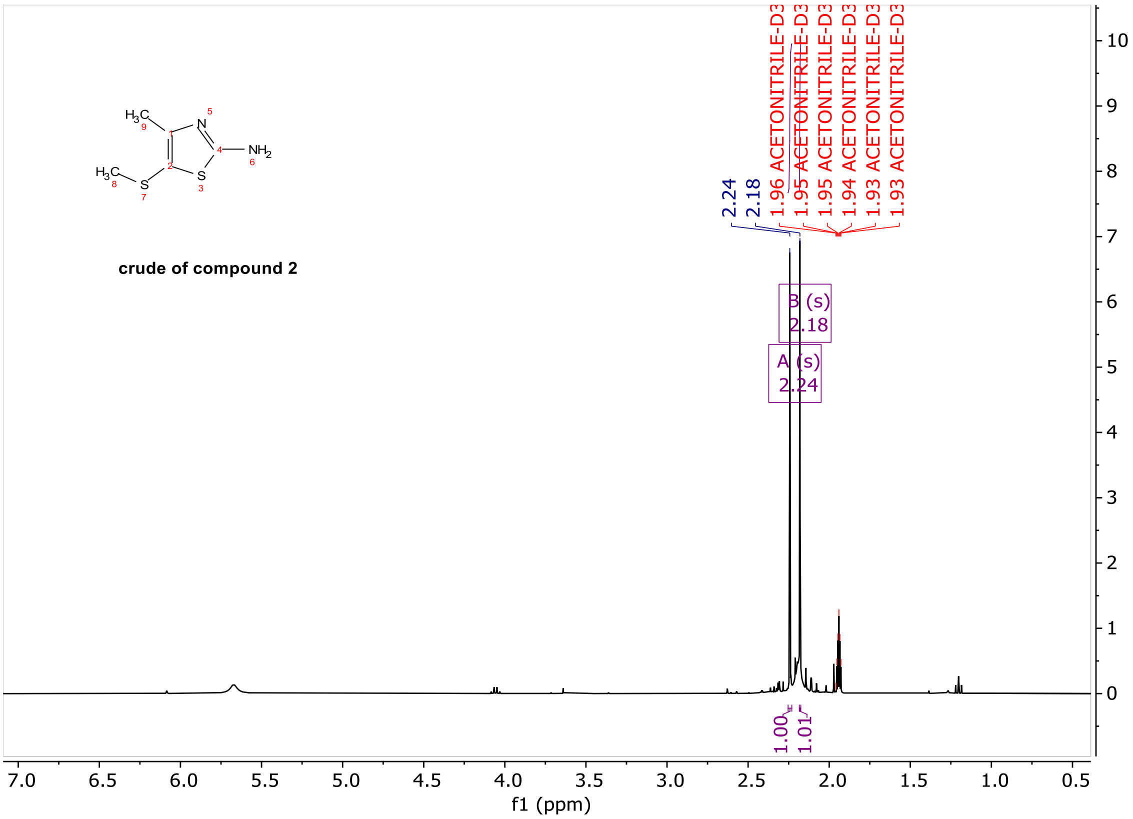

Figure S4: ^{1}H NMR spectrum of crude **D2**.

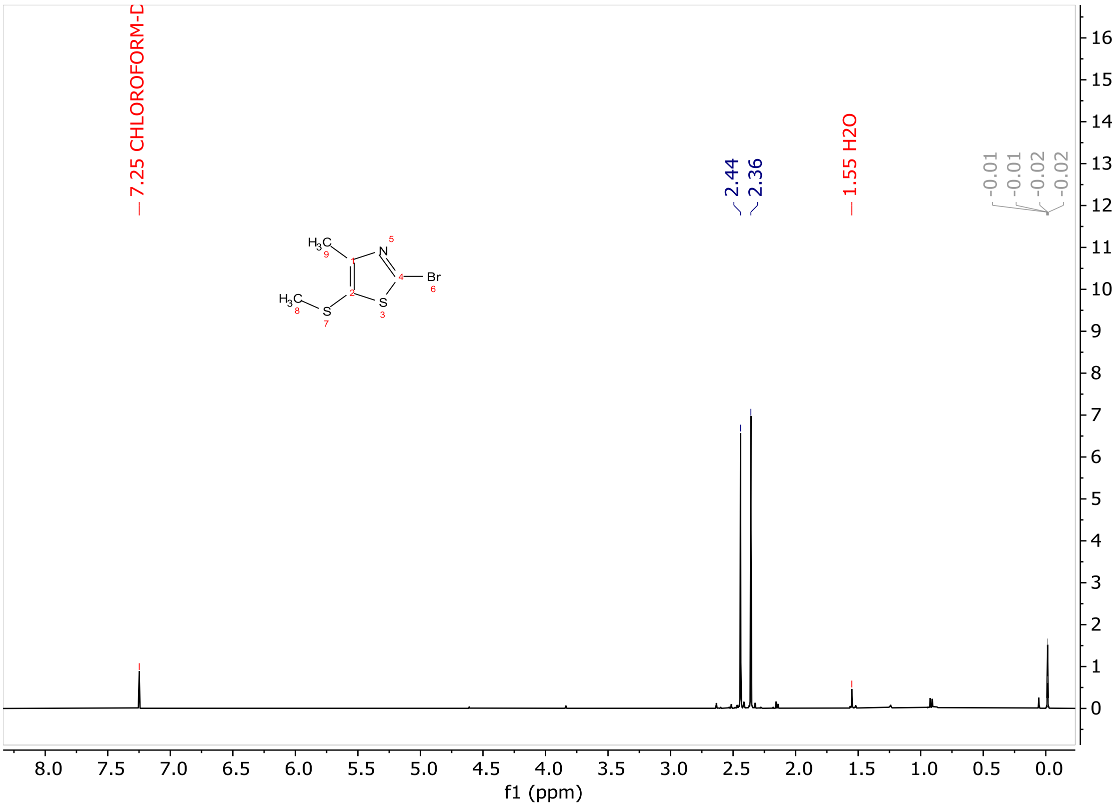

Figure S5: ^{1}H NMR spectrum of **D3**.

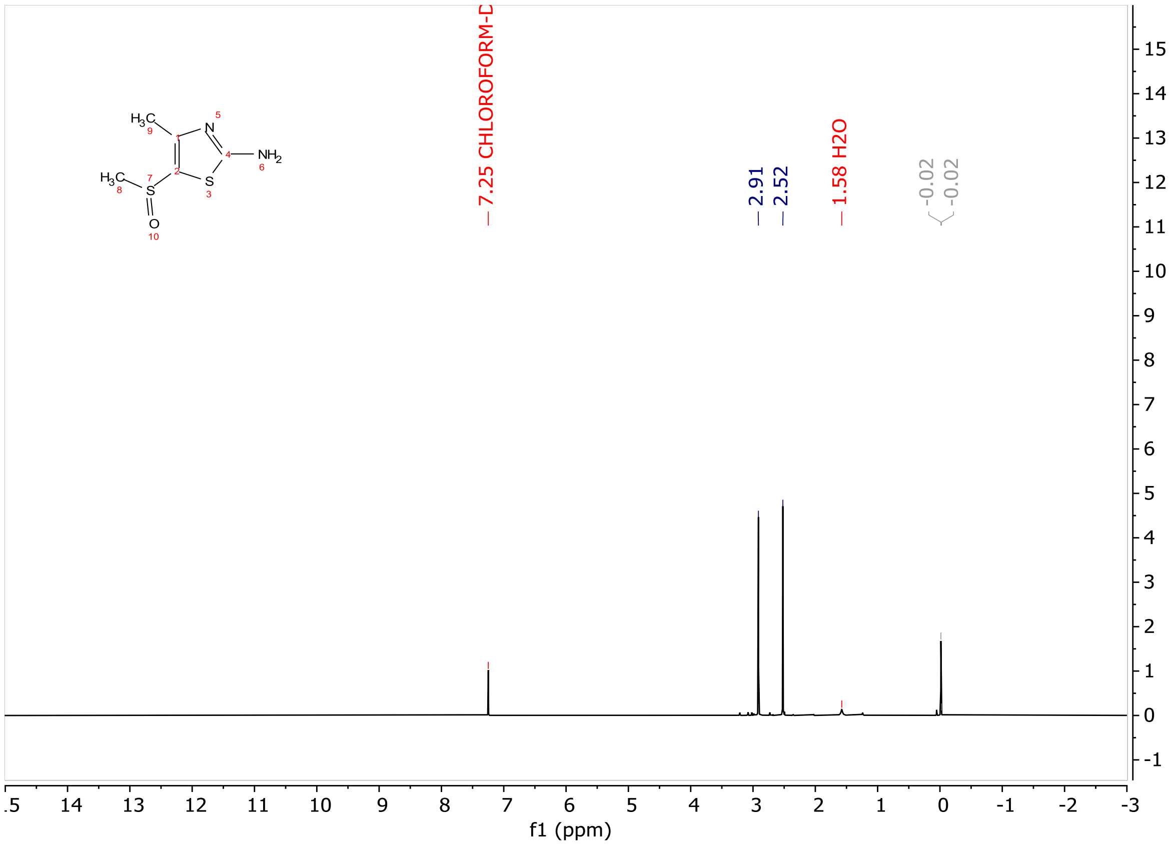

Figure S6: ^{1}H NMR spectrum of **D4**.

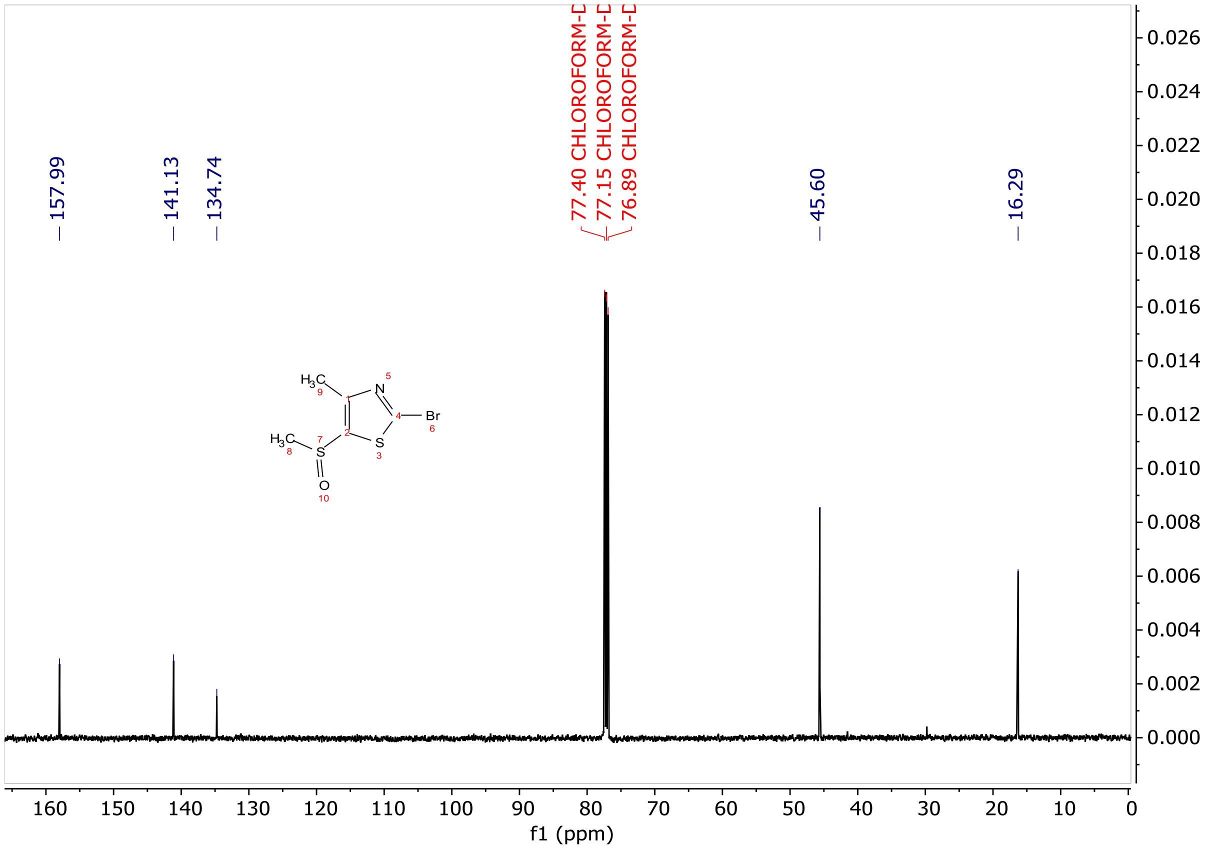

Figure S7: ^{13}C NMR spectrum of **D4**.

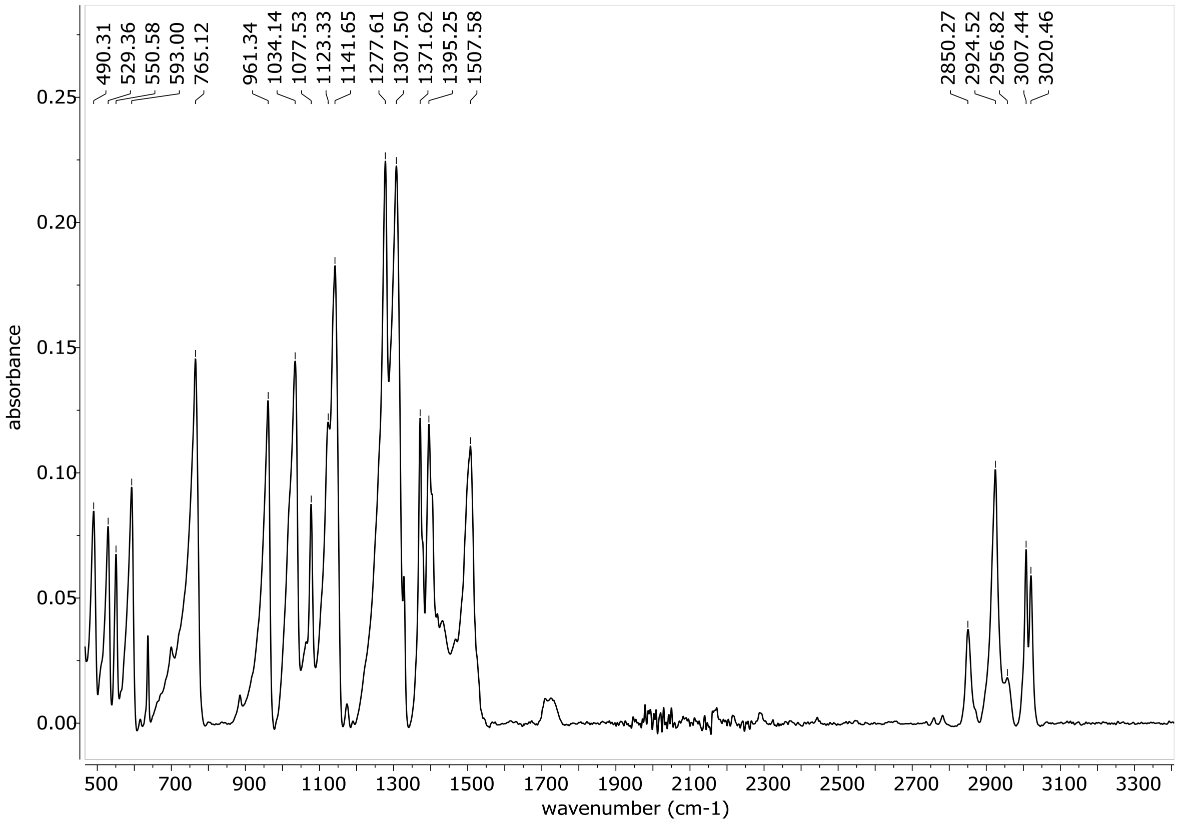

Figure S8: ATR-FTIR spectrum of **D4**.

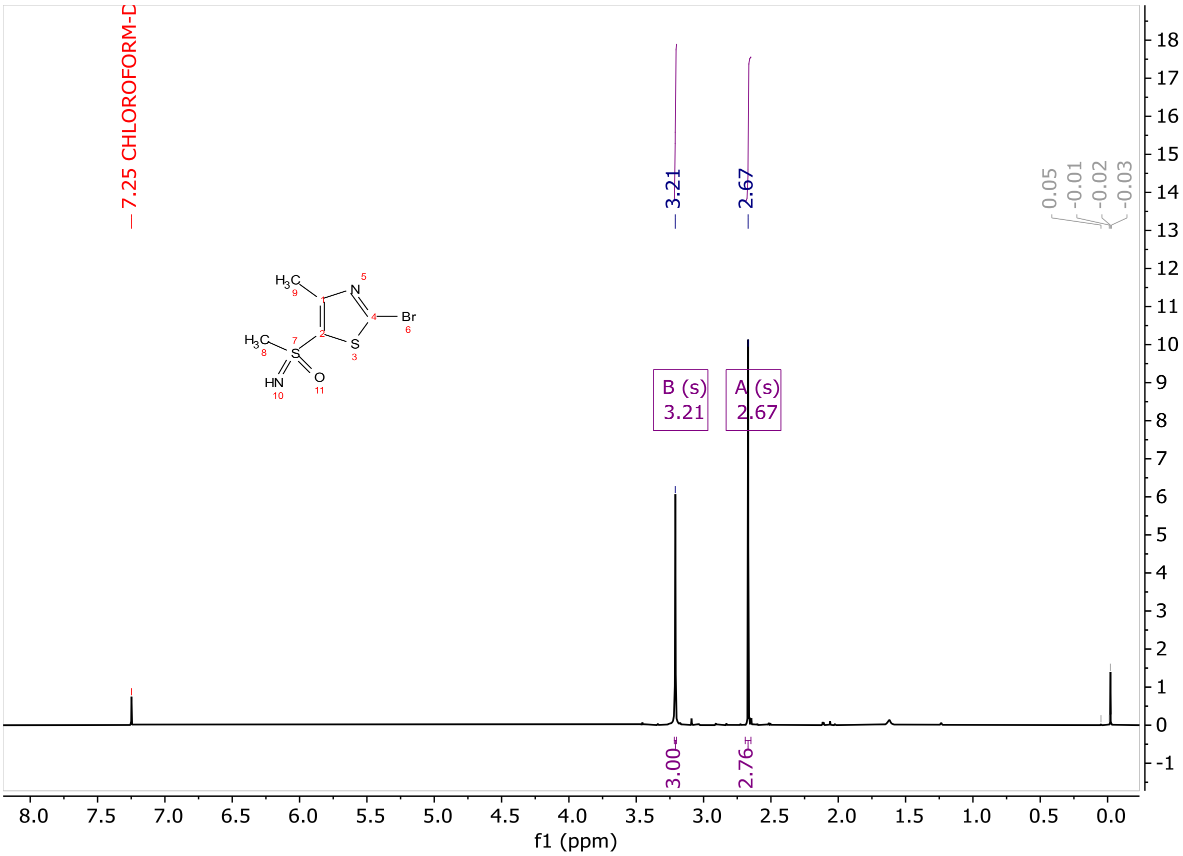

Figure S9: ^{1}H NMR spectrum of **D5**.

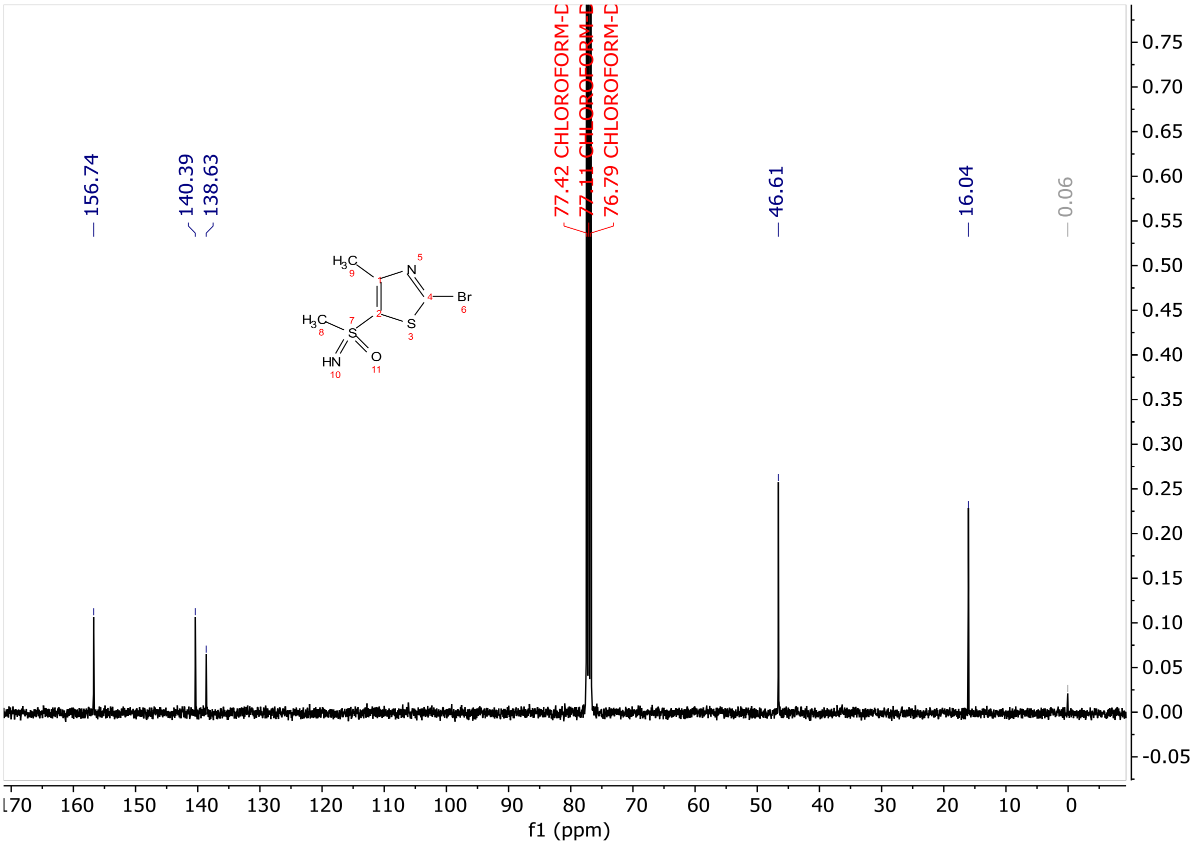

Figure S10: ^{13}C NMR spectrum of **D5**.

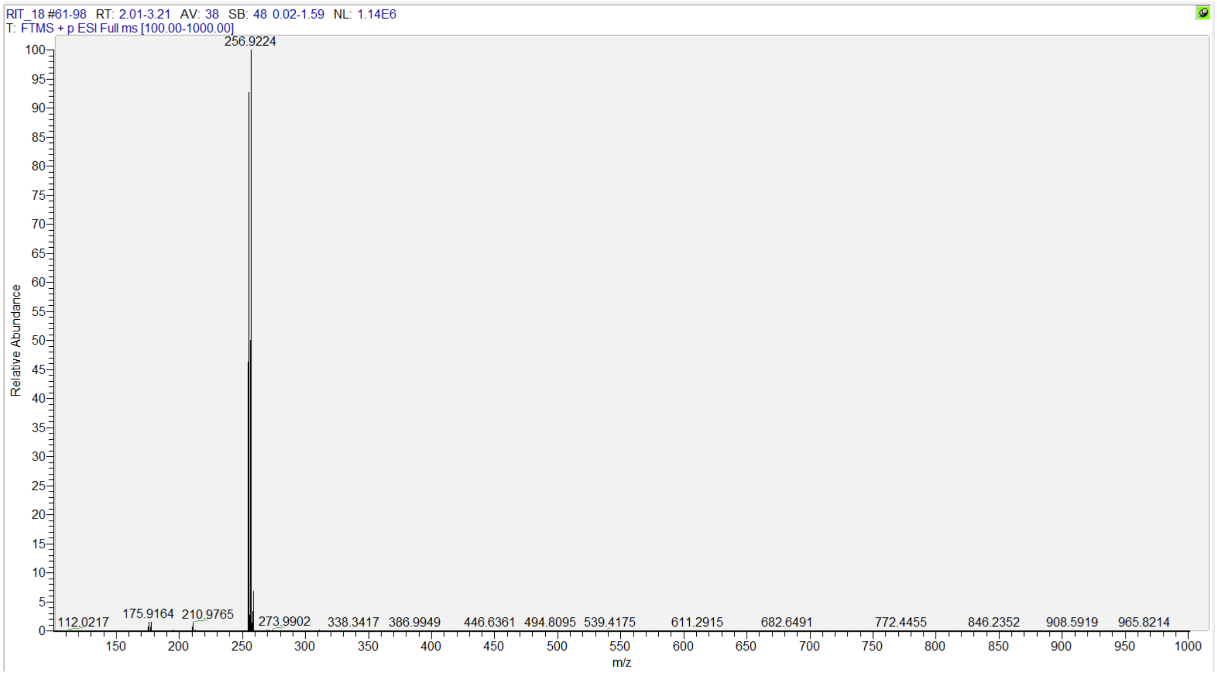

Figure S11: HRMS spectrum of **D5**. Calc: 253.9183 [M]; 254.9256 [M+H$^+$]. Found: 254.9248, 256.9224 [M+H$^+$].

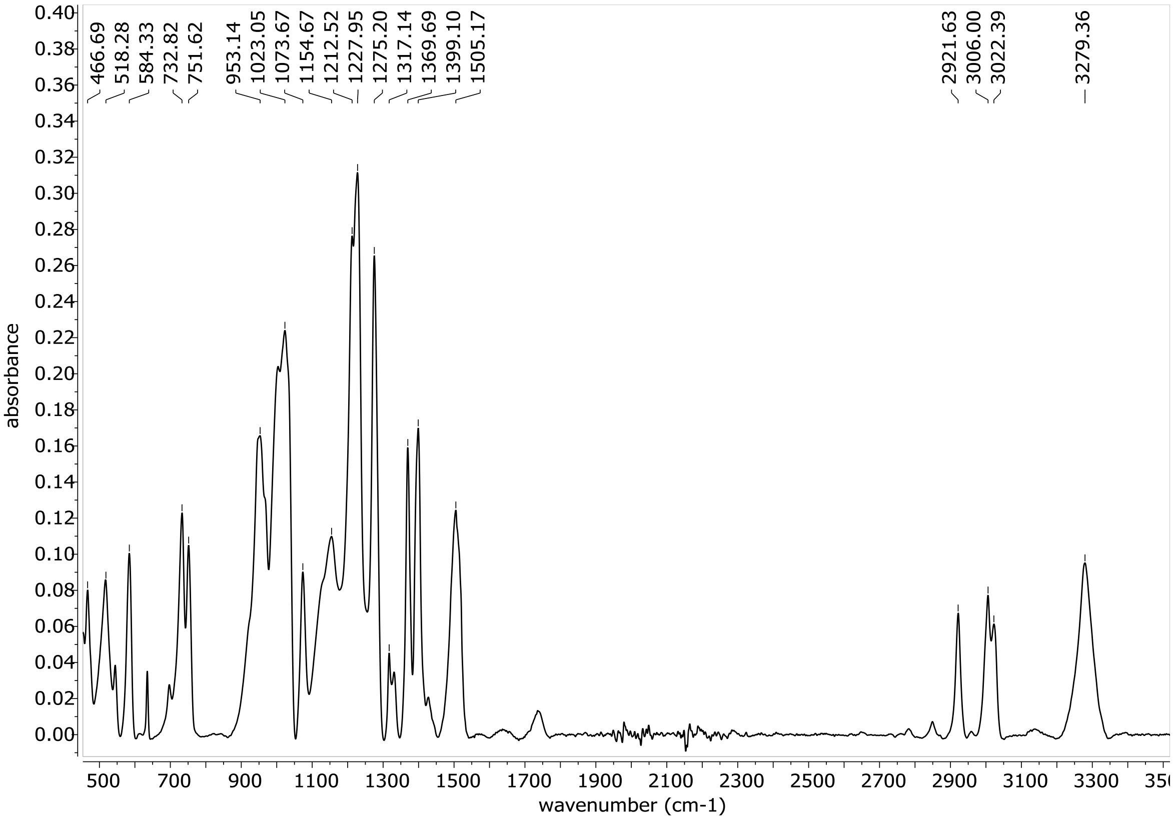

Figure S12: ATR-FTIR spectrum of **D5**.

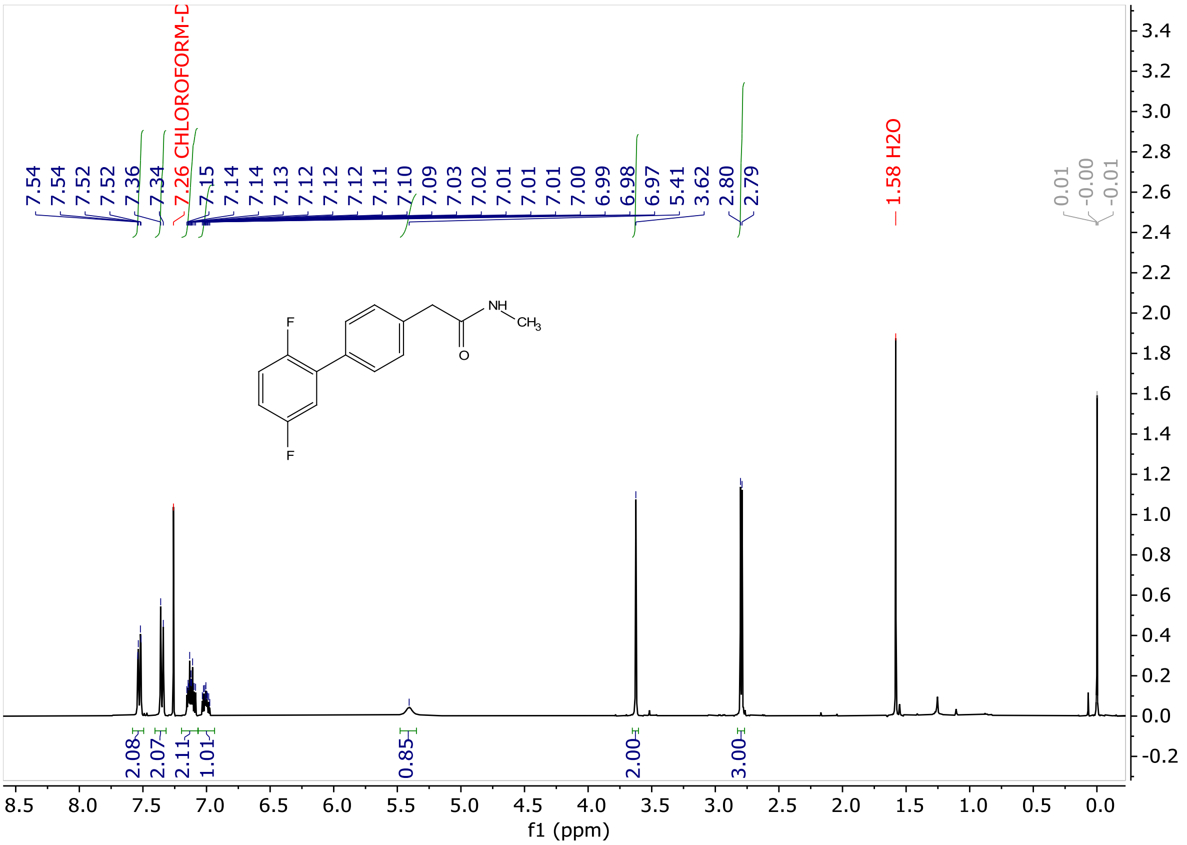

Figure S13: ^{1}H NMR spectrum of **D8**.

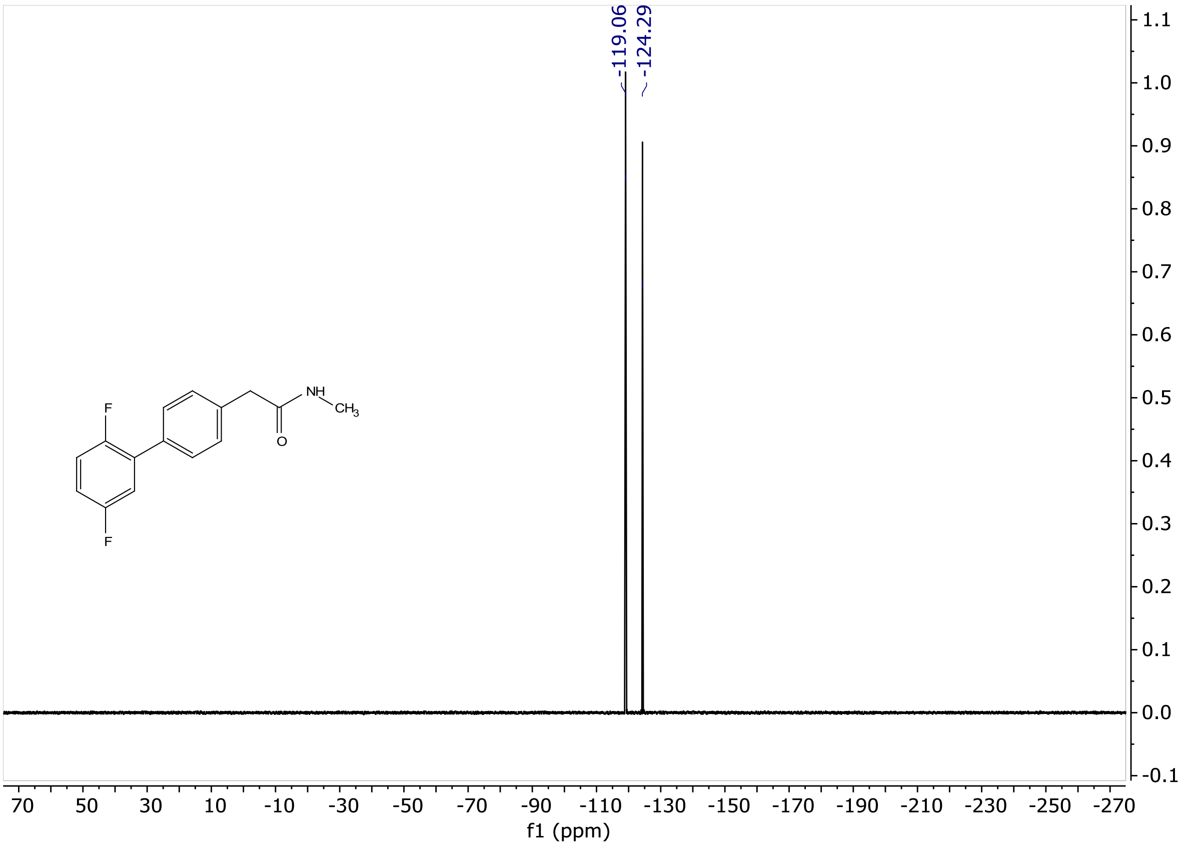

Figure S14: ^{19}F NMR spectrum of **D8**.

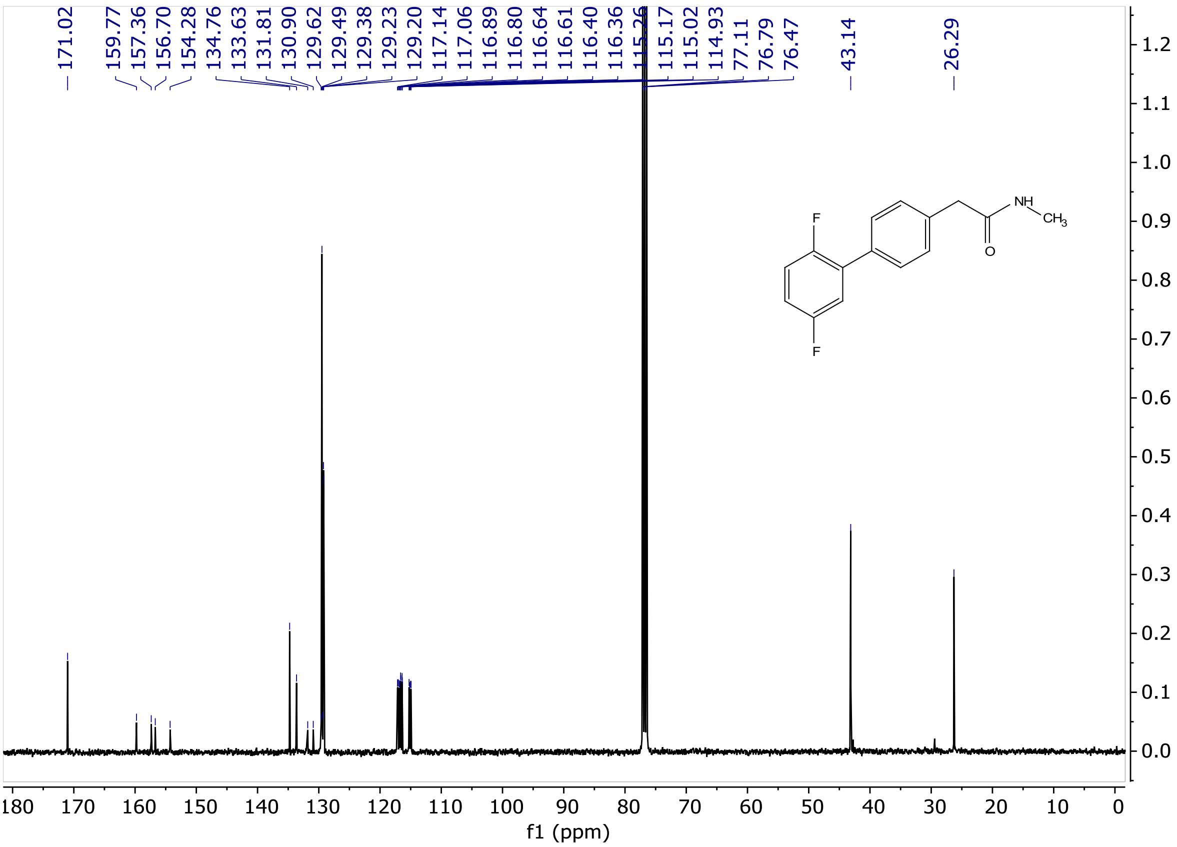

Figure S15: ^{13}C NMR spectrum of **D8**.

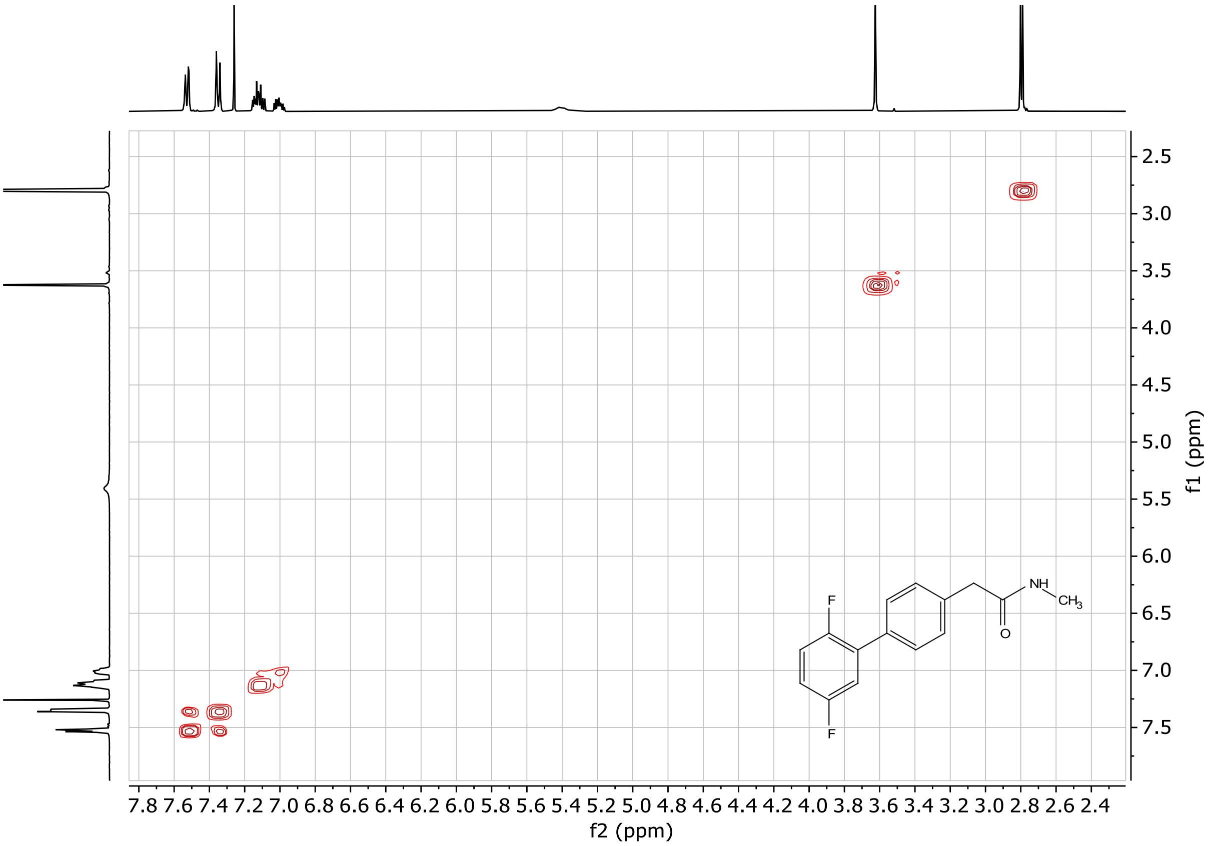

Figure S16: COSY spectrum of **D8**.

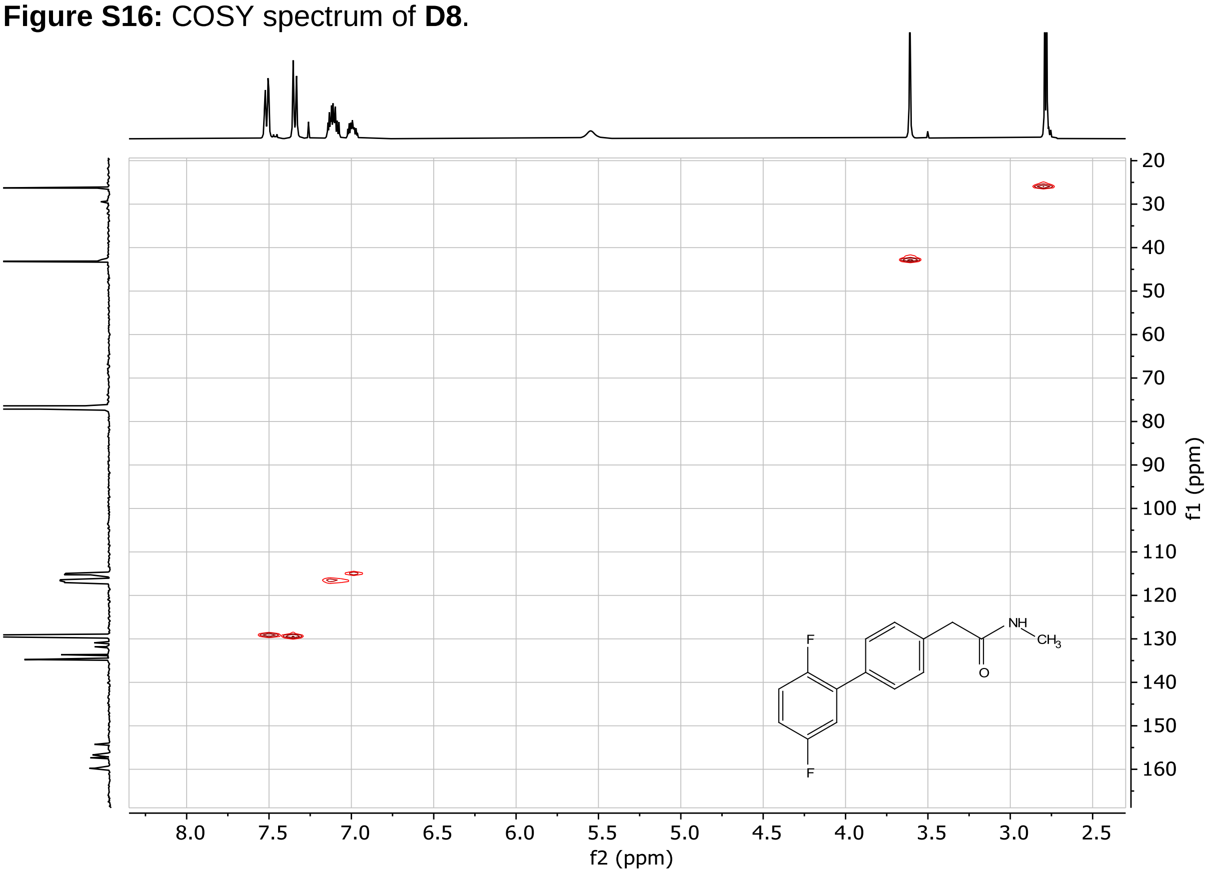

Figure S17: HSQC spectrum of **D8**.

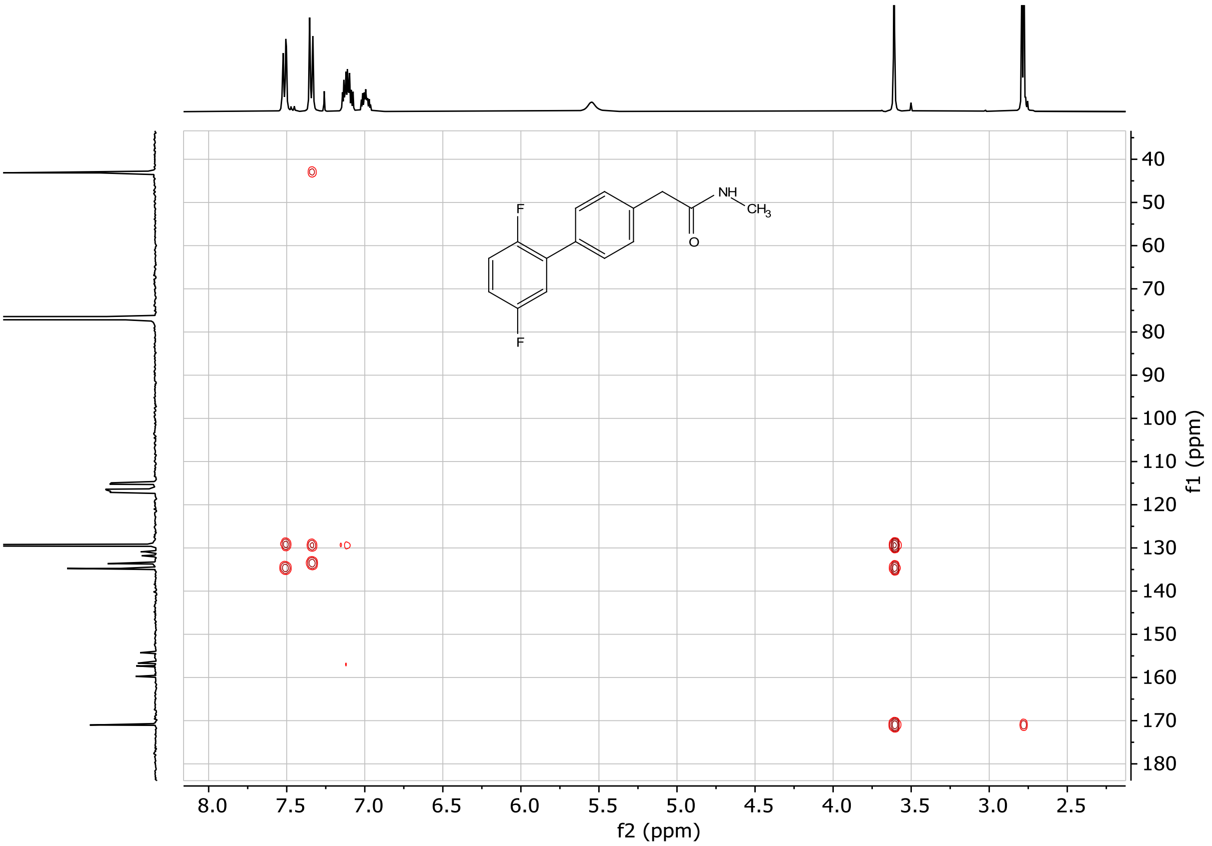

Figure S18: HMBC spectrum of **D8**.

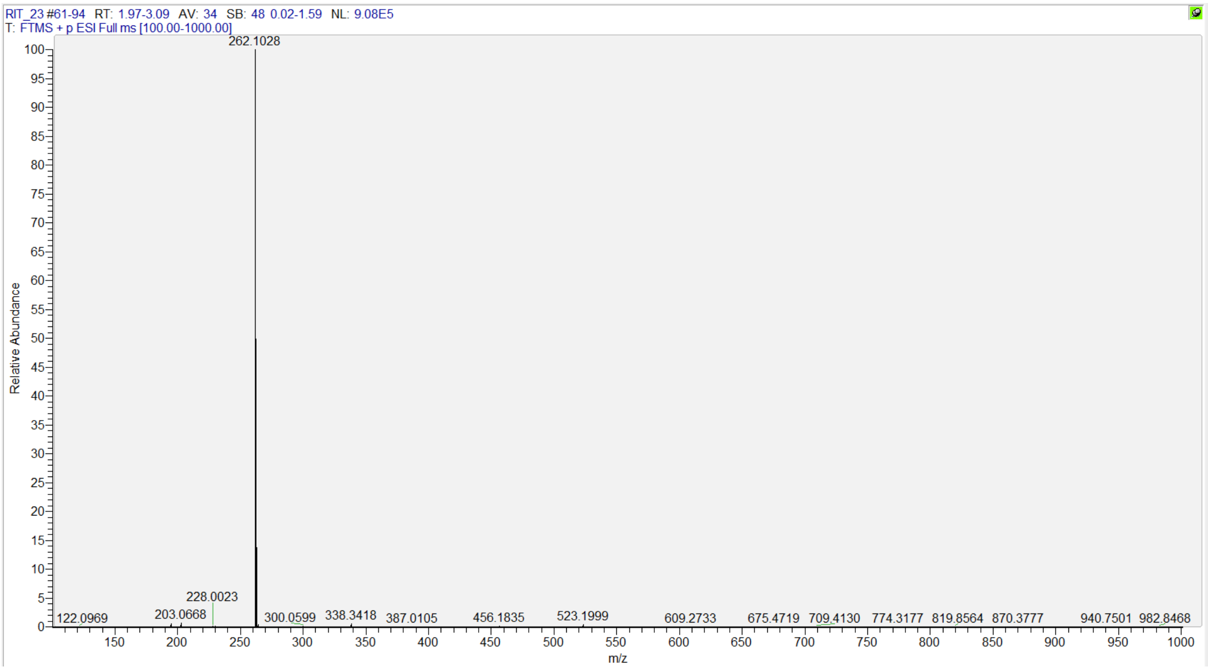

Figure S19: HRMS spectrum of **D8**. Calc: 261.0965 [M]; 262.1038 [M+H$^+$]. Found: 262.1028 [M+H$^+$].

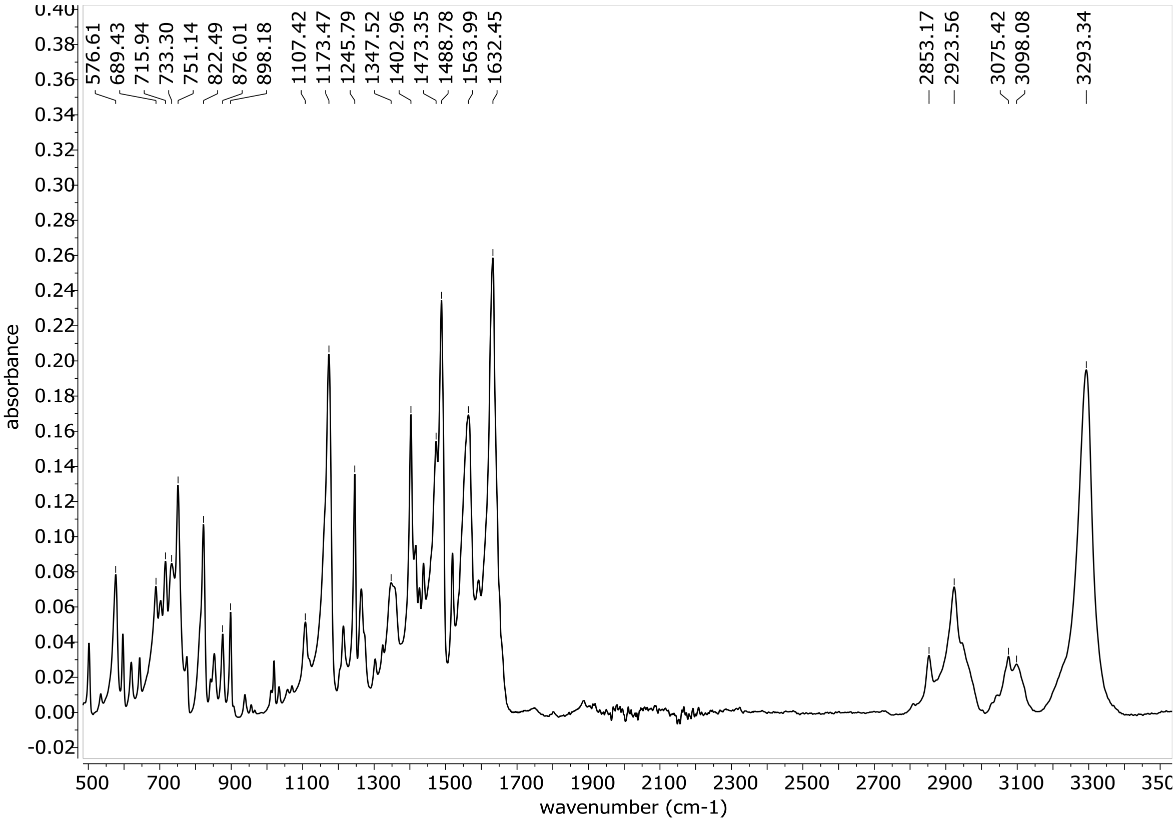

Figure S20: ATR-FTIR spectrum of **D8**.

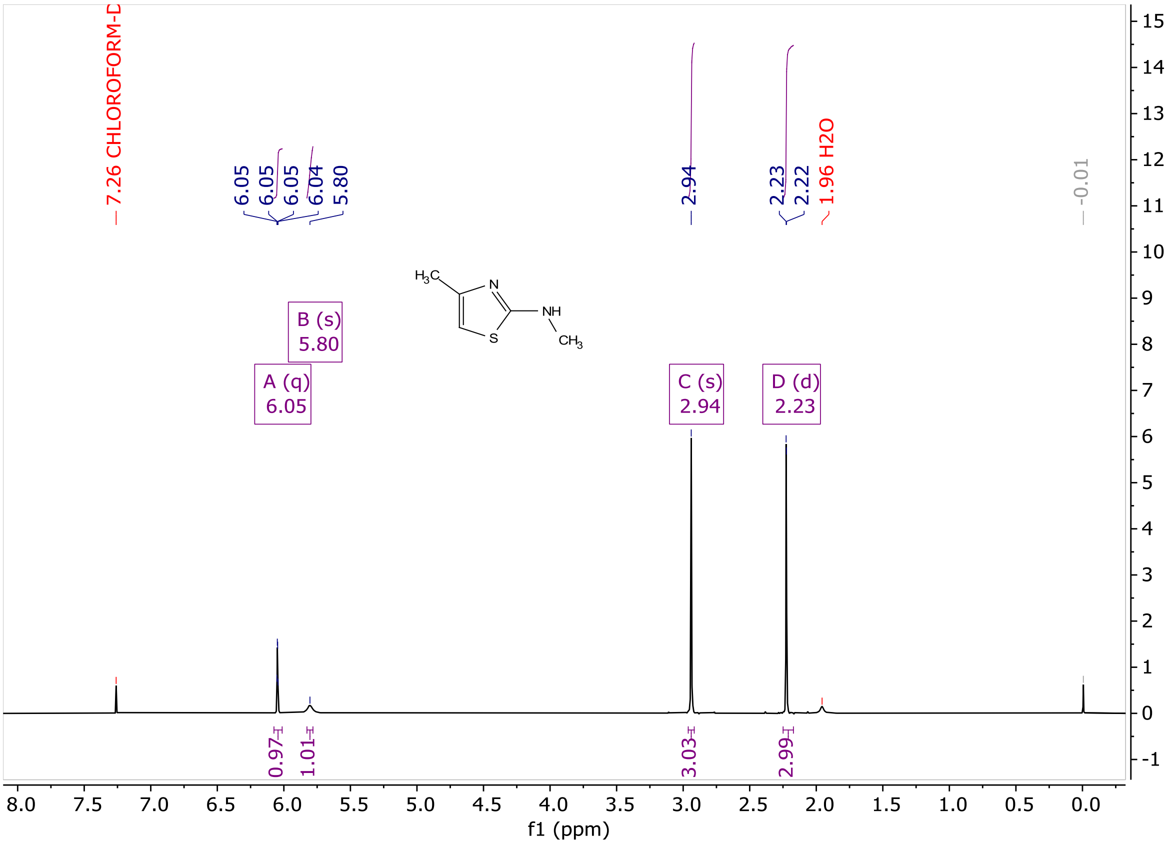

Figure S21: ^{1}H NMR spectrum of **E3**.

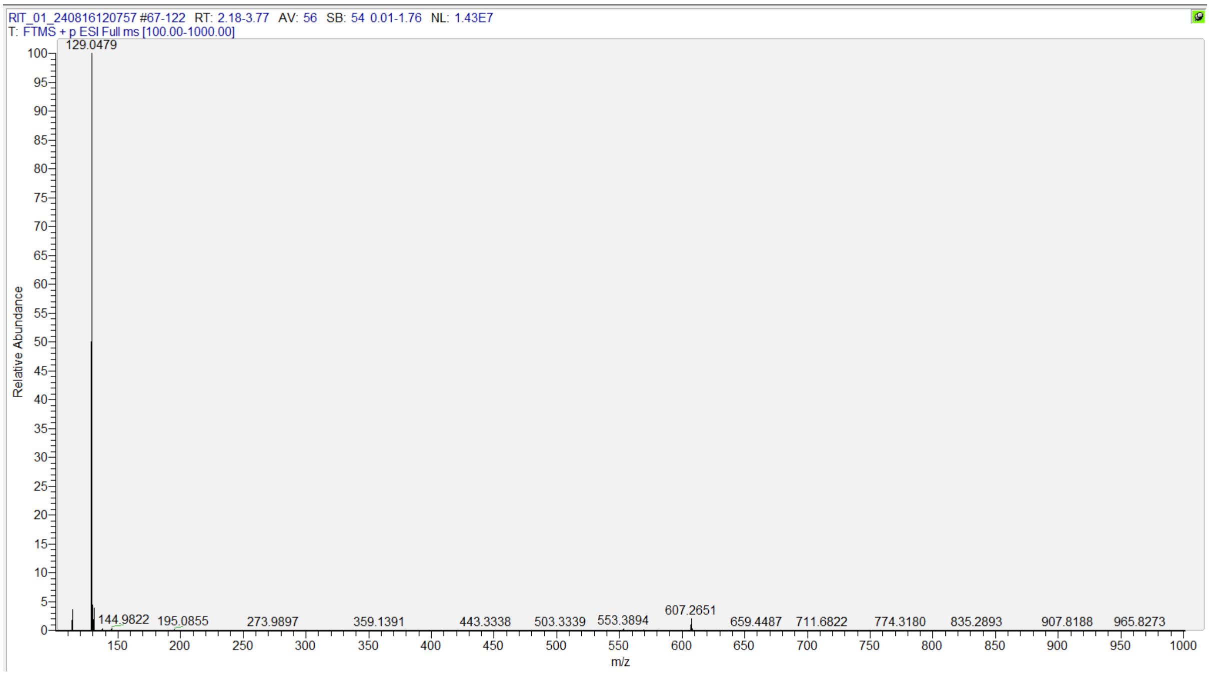

Figure S22: HRMS spectrum of **E3**. Calc: 128.0408 [M]; 129.0481 [M+H$^+$]. Found: 129.0479 [M+H$^+$].

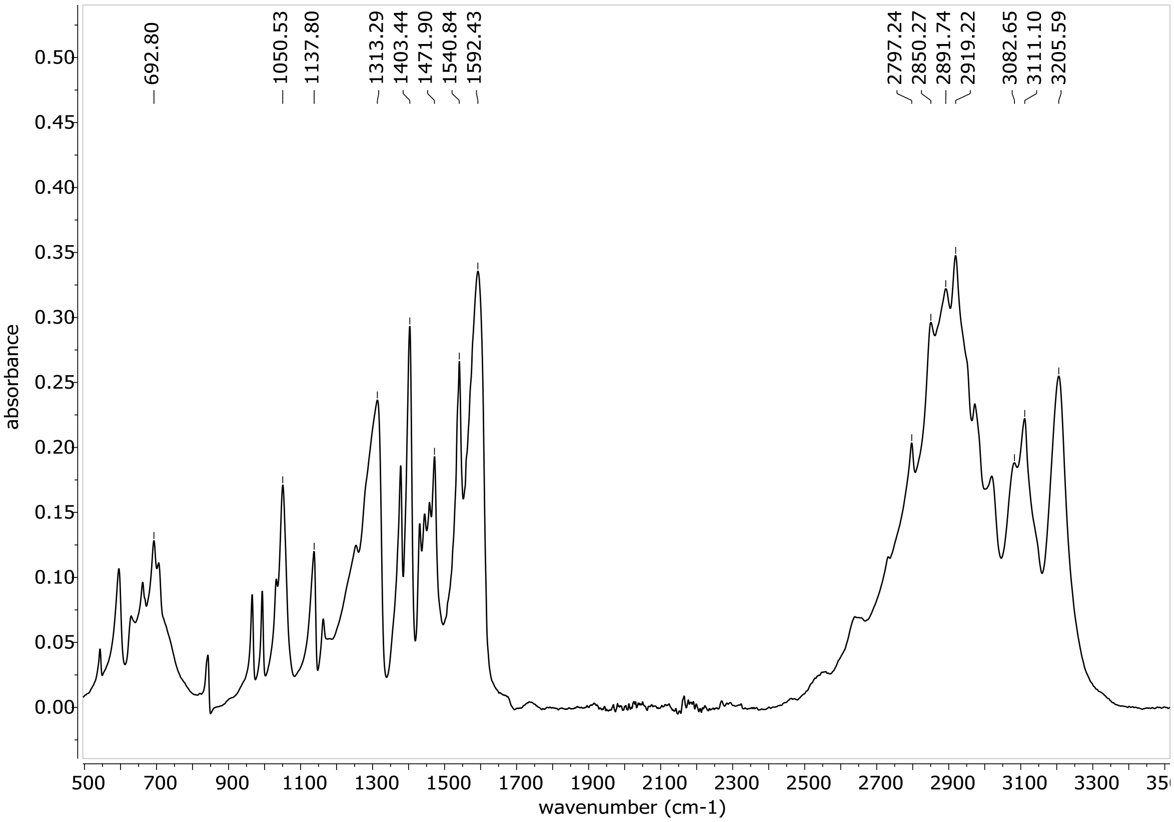

Figure S23: ATR-FTIR spectrum of **E3**.

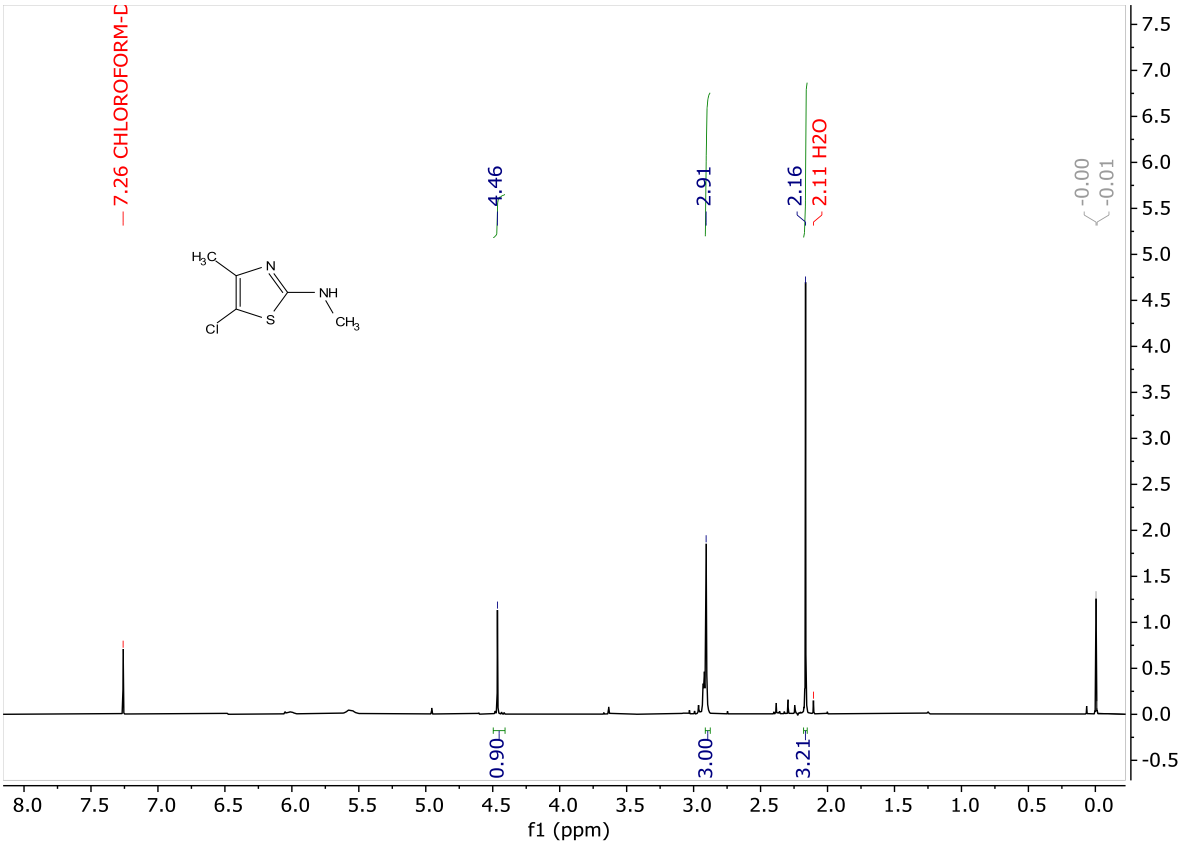

Figure S24: ^{1}H NMR spectrum of **E4**.

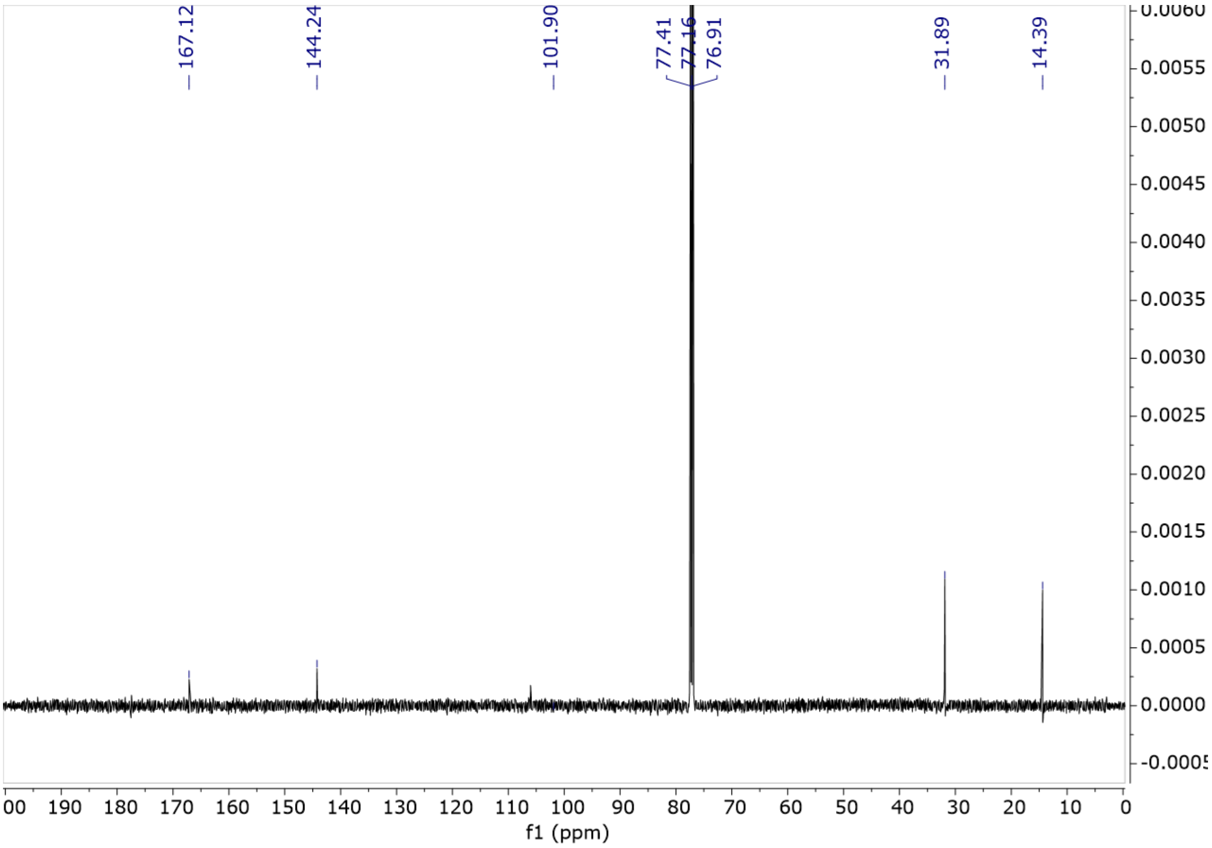

Figure S25: ^{13}C NMR spectrum of **E4**.

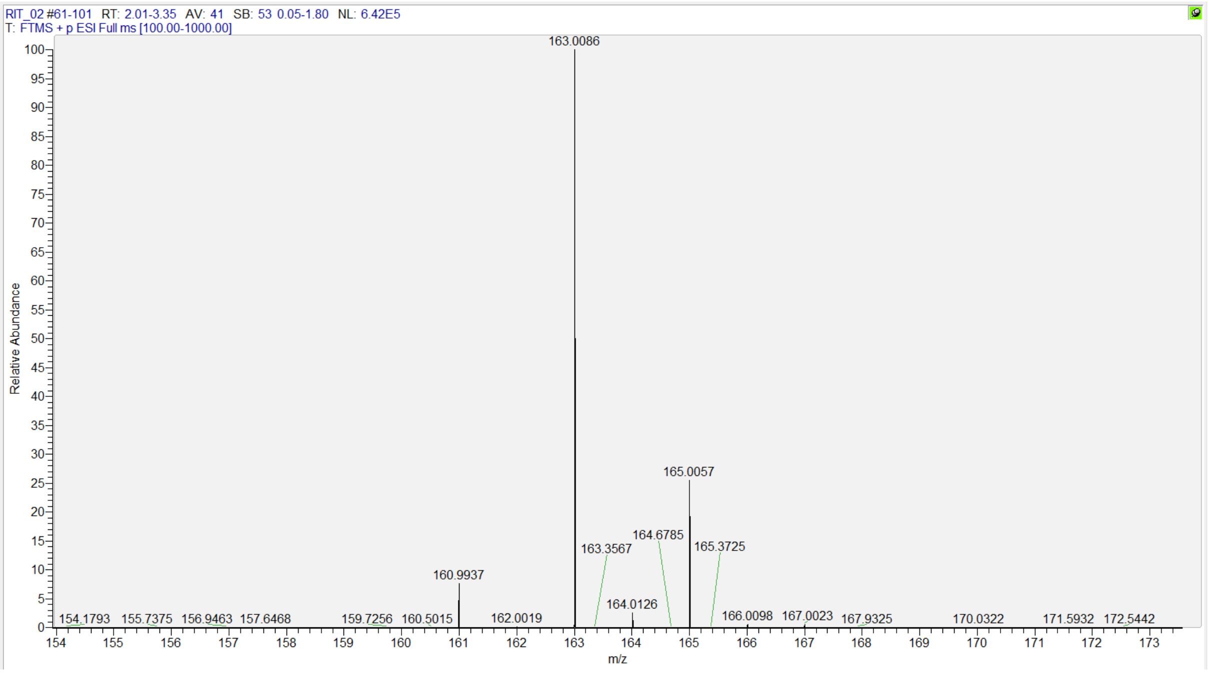

Figure S26: HRMS spectrum of **E4**. Calc: 162.0018 [M]; 163.0091 [M+H$^+$]. Found: 163.0086 [M+H$^+$].

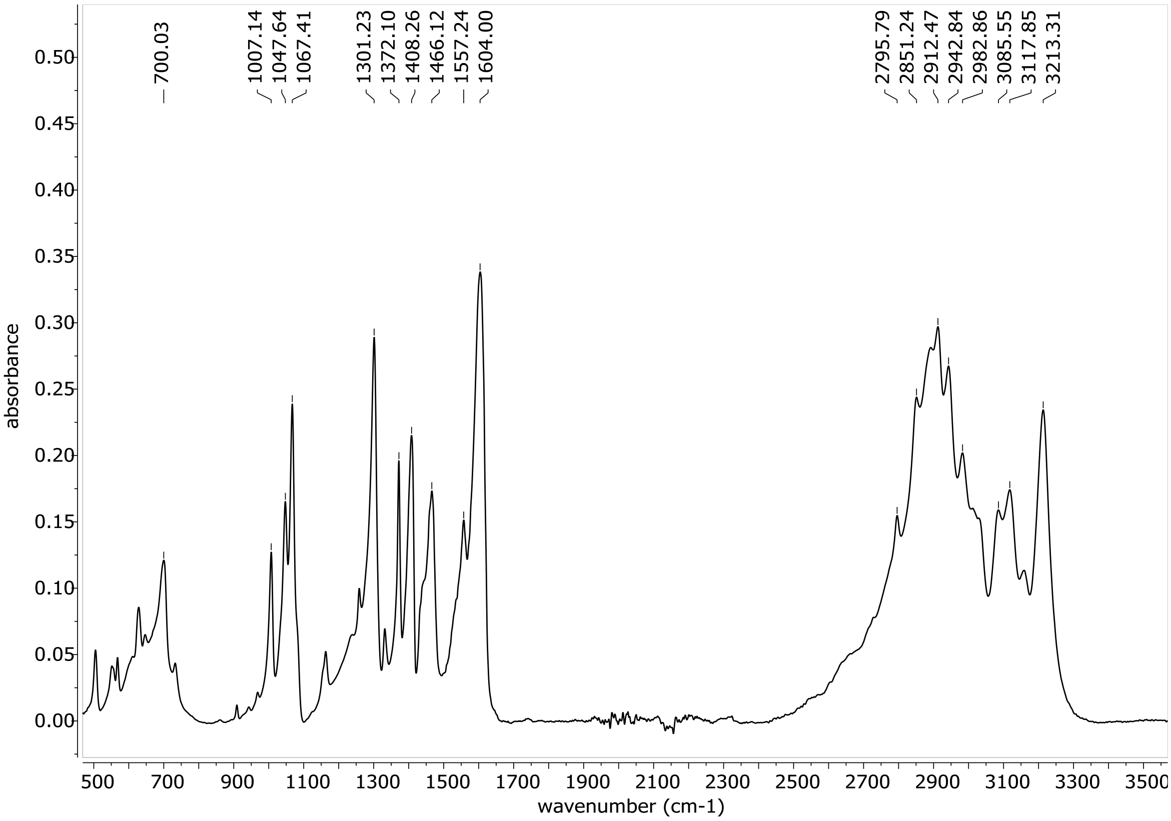

Figure S27: ATR-FTIR spectrum of **E4**.

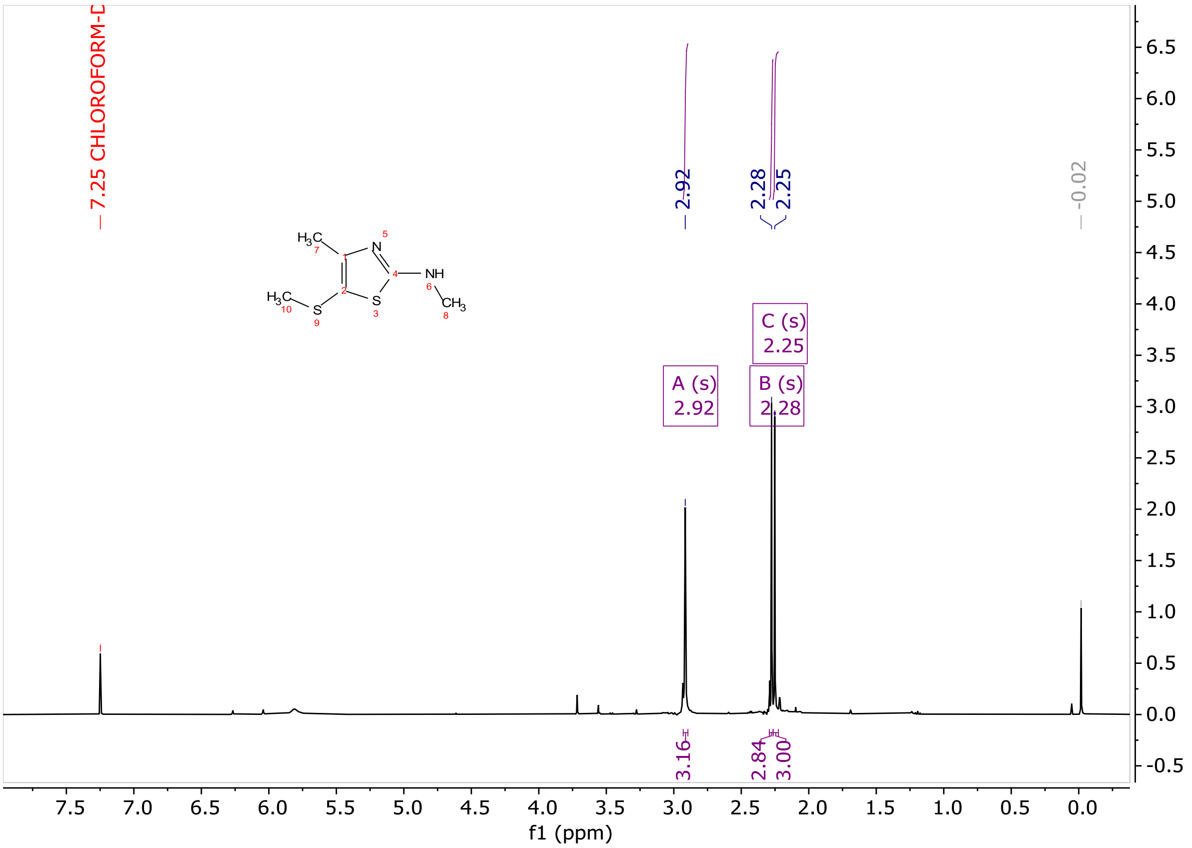

Figure S28: ^{1}H NMR spectrum of **E5**.

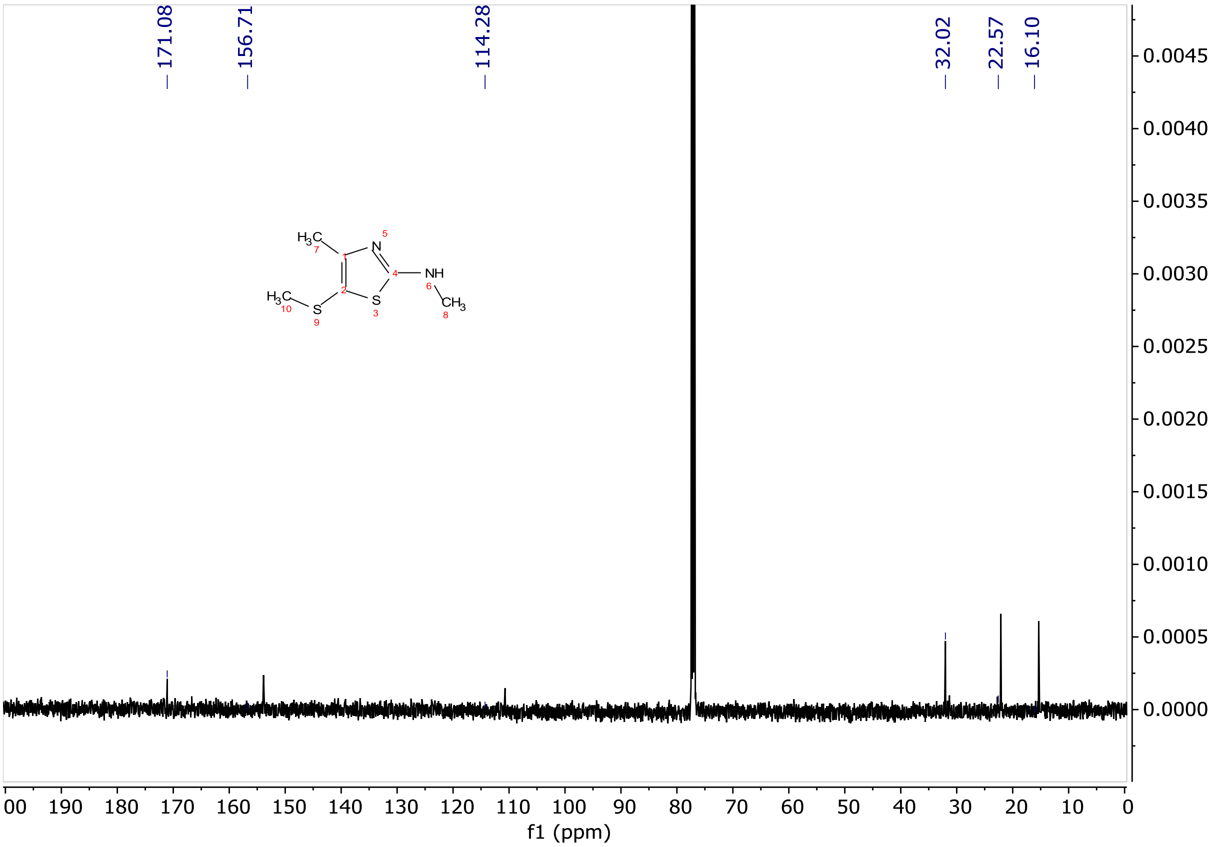

Figure S29: ^{13}C NMR spectrum of **E5**.

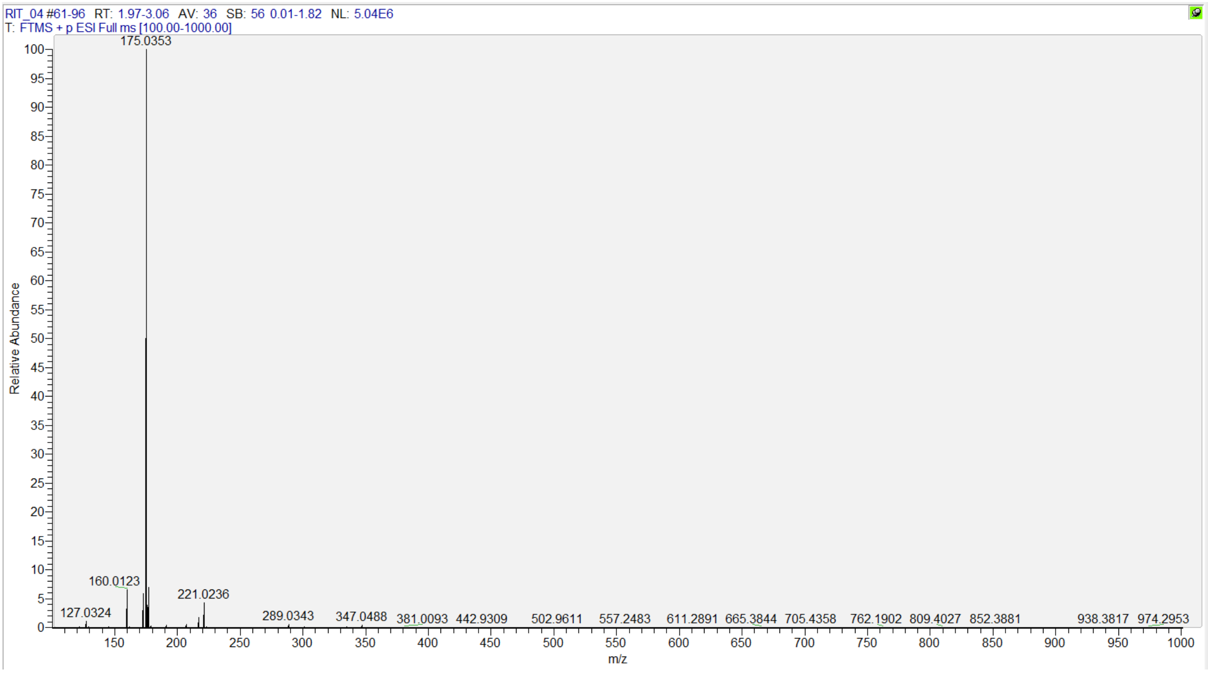

Figure S30: HRMS spectrum of **E5**. Calc: 174.0285 [M]; 175.0358 [M+H$^+$]. Found: 175.0353 [M+H$^+$].

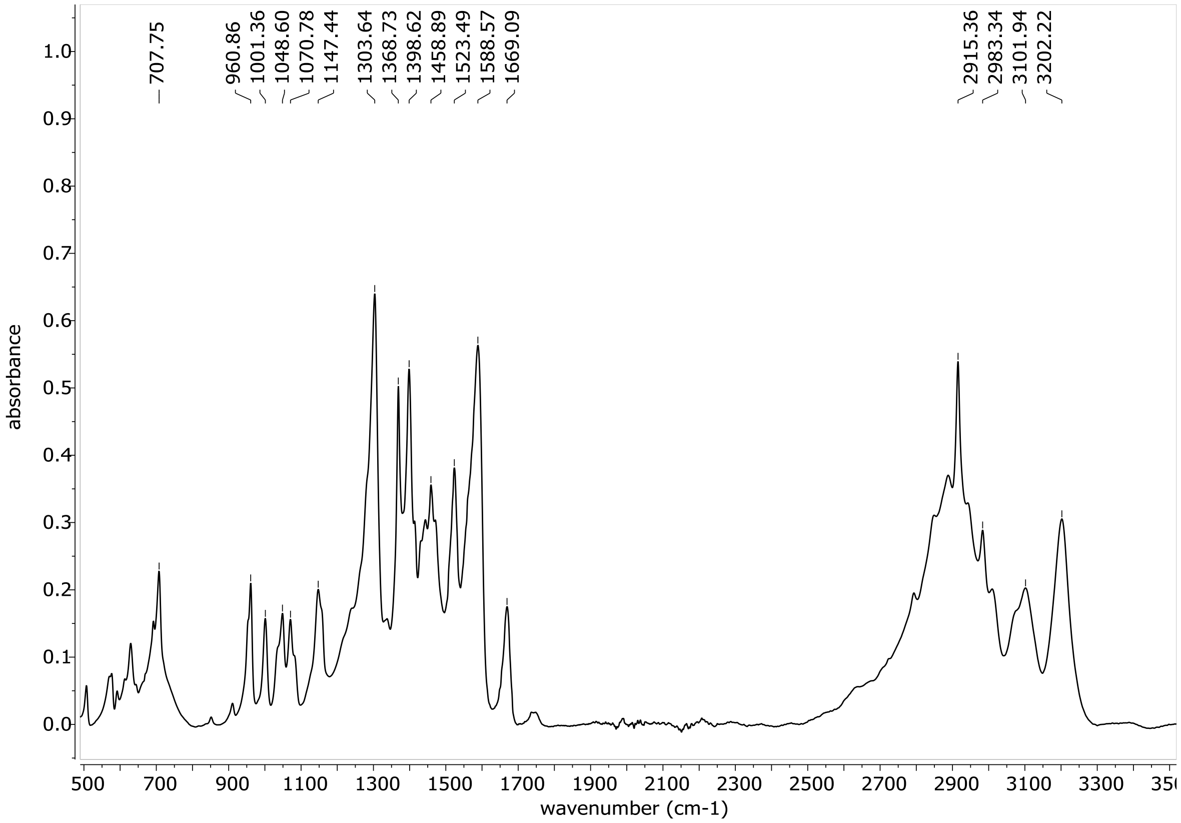

Figure S31: ATR-FTIR spectrum of **E5**.

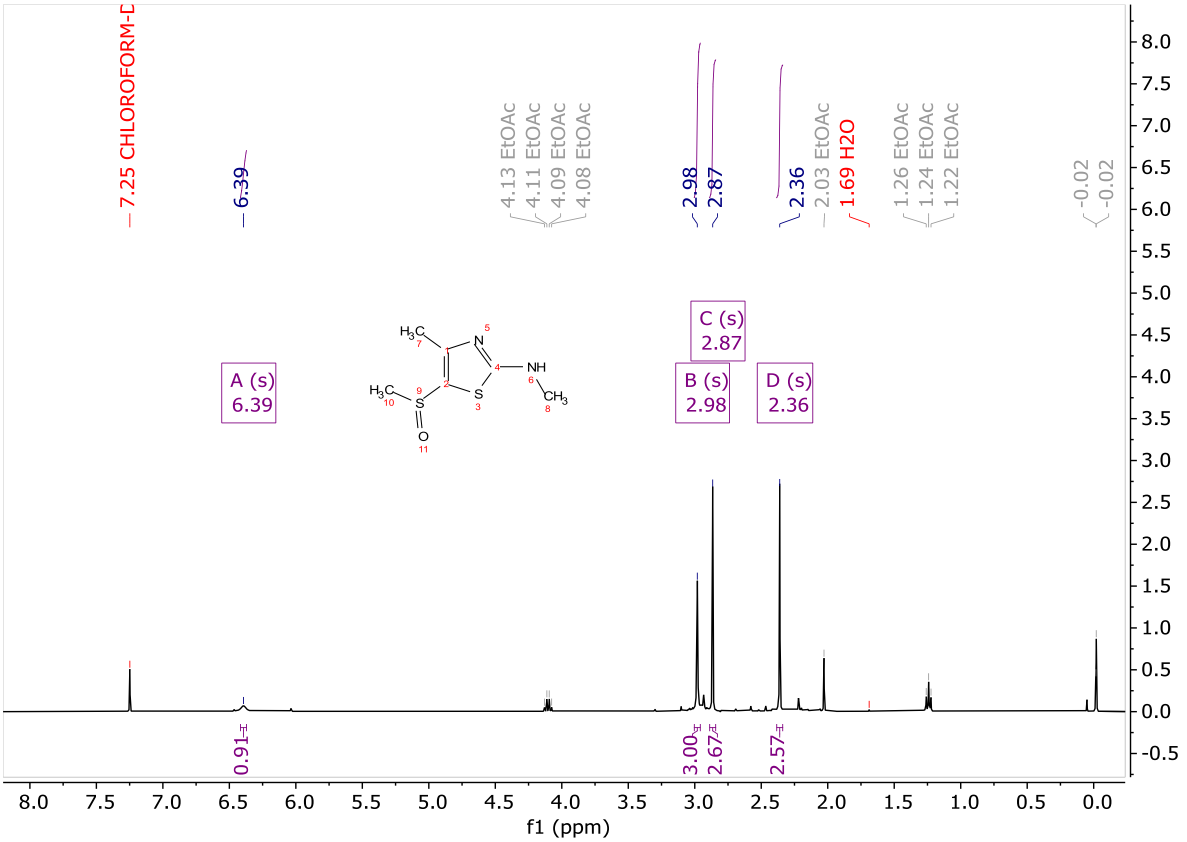

Figure S32: ^{1}H NMR spectrum of **E6**.

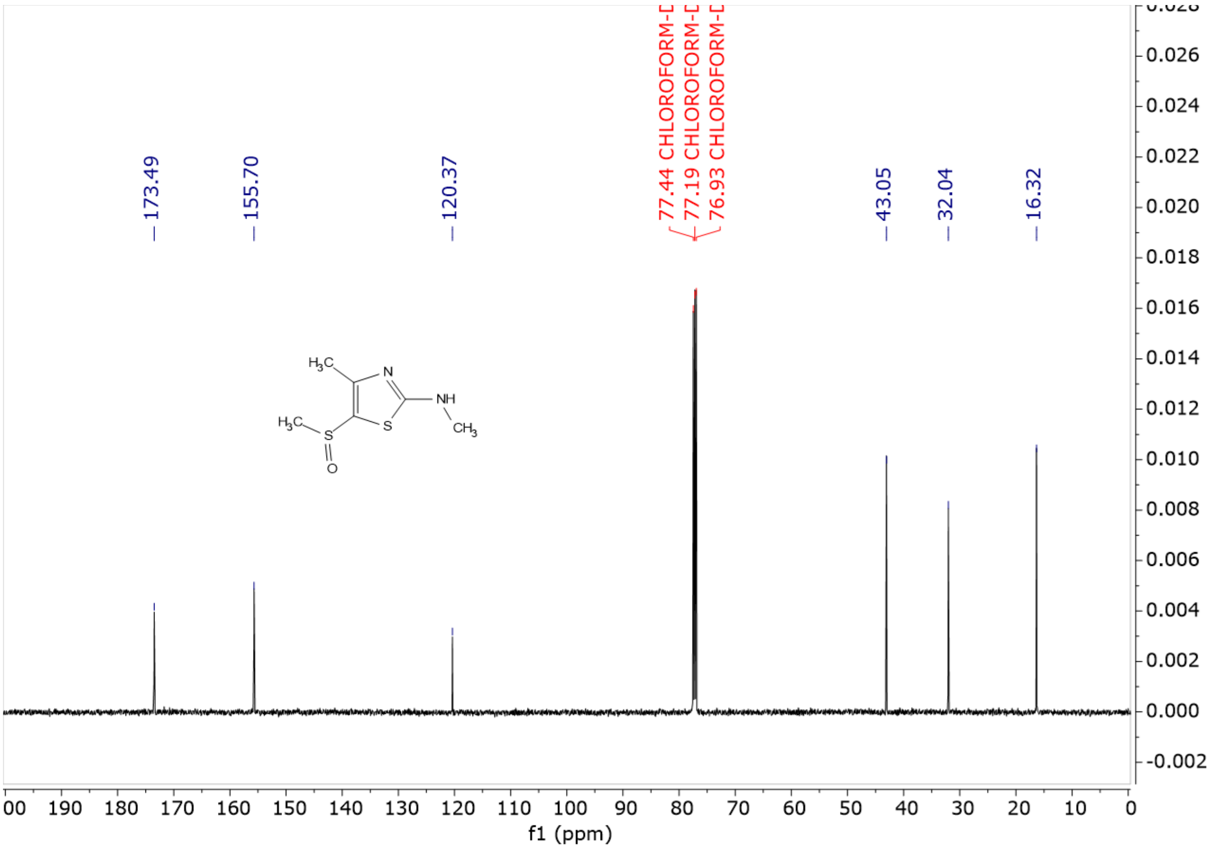

Figure S33: ^{13}C NMR spectrum of **E6**.

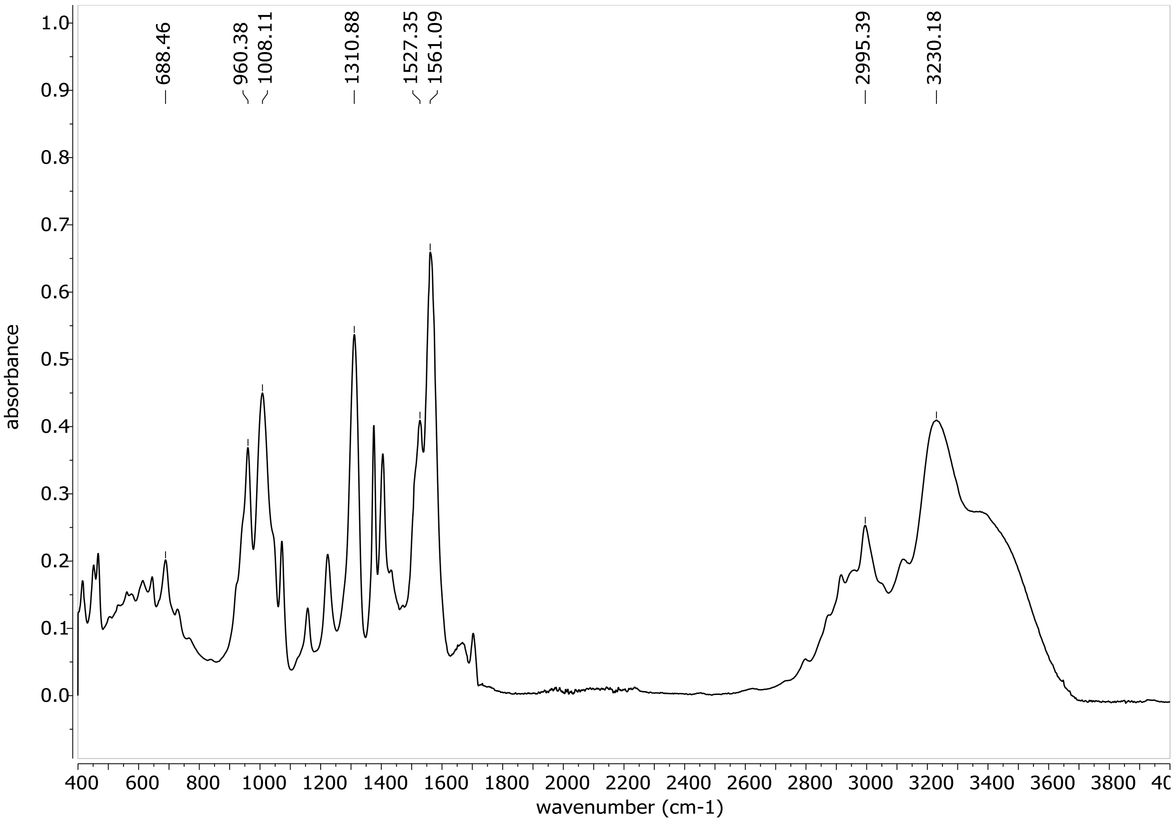

Figure S34: FTIR spectrum of **E6**. Spectrum was recorded on MeOH and the solvent subtracted.

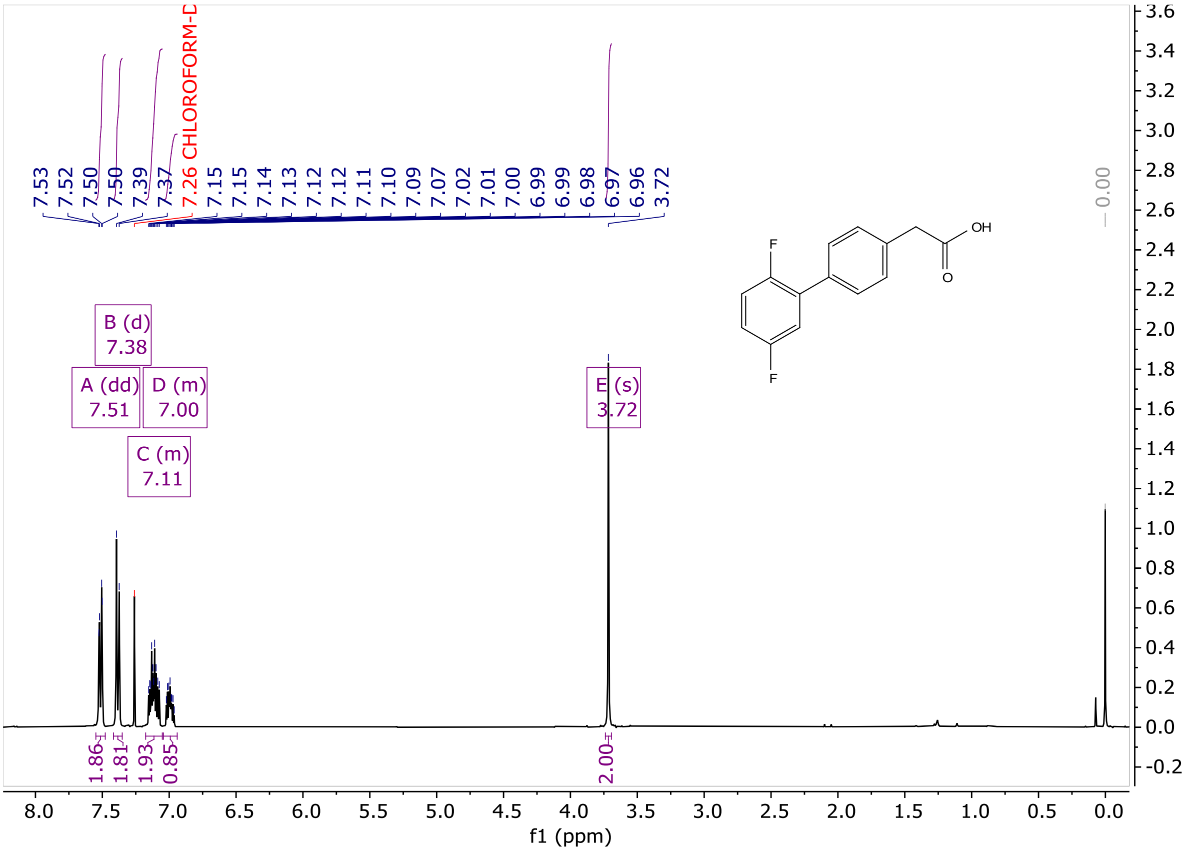

Figure S35: ^{1}H NMR spectrum of **E9**.

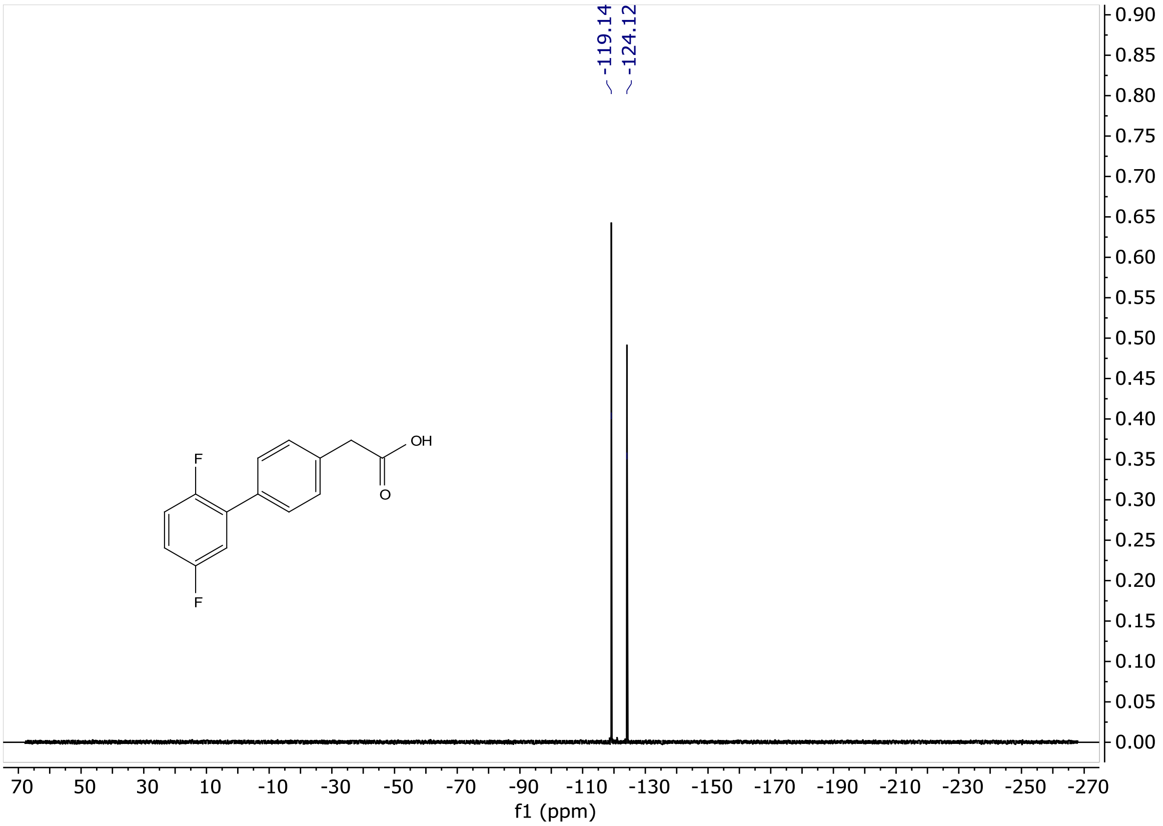

Figure S36: ^{19}F NMR spectrum of **E9**.

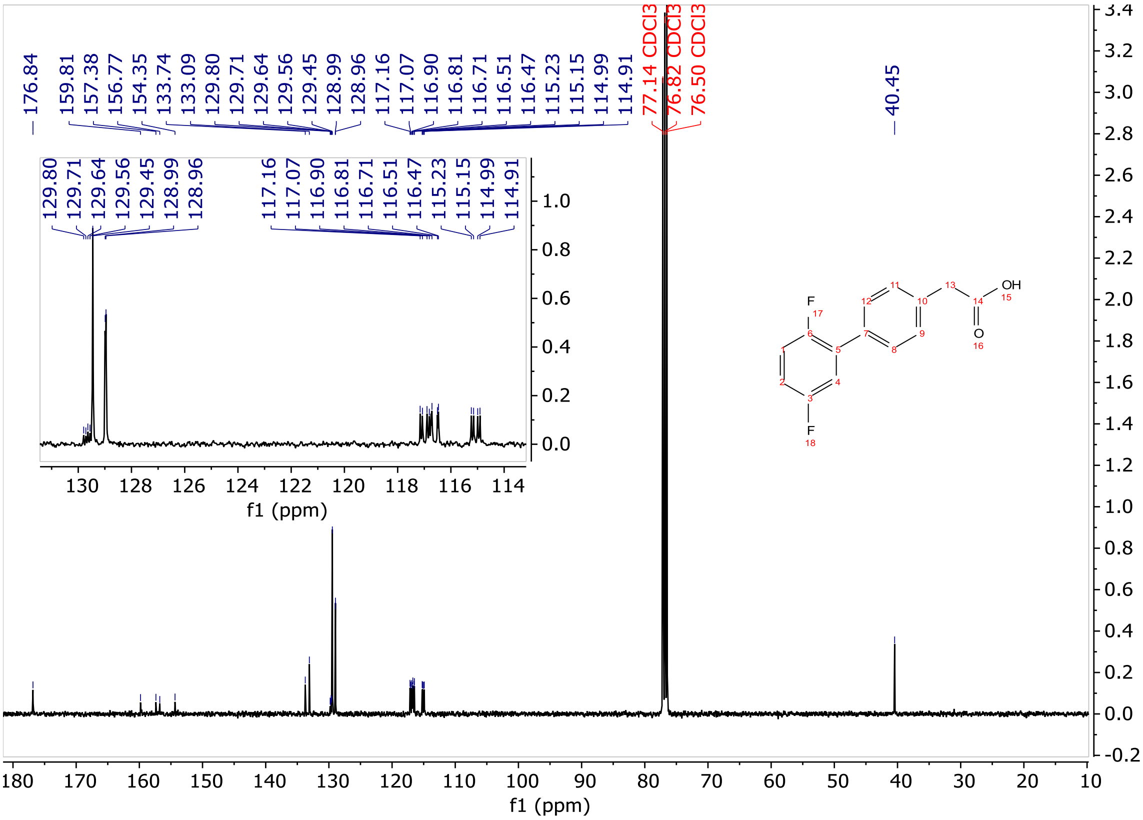

Figure S37: ^{13}C NMR spectrum of **E9**.

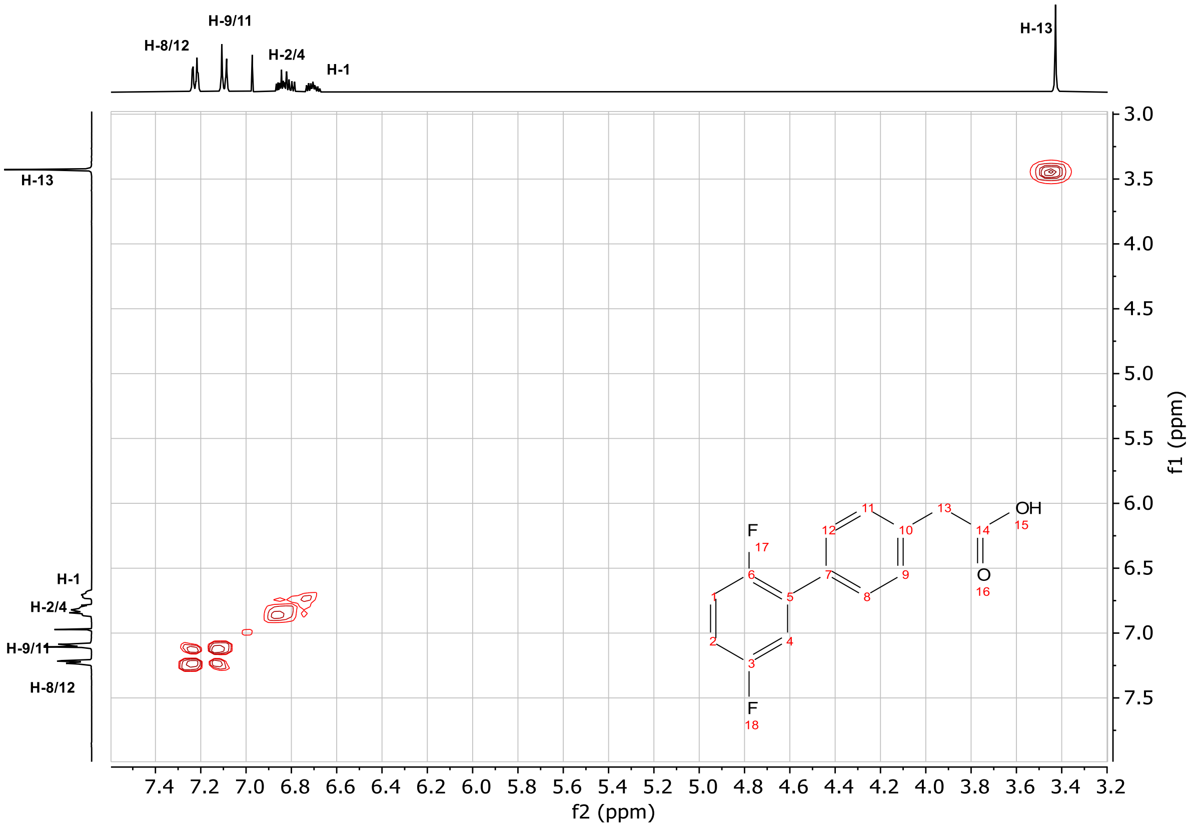

Figure S38: COSY spectrum of **E9**.

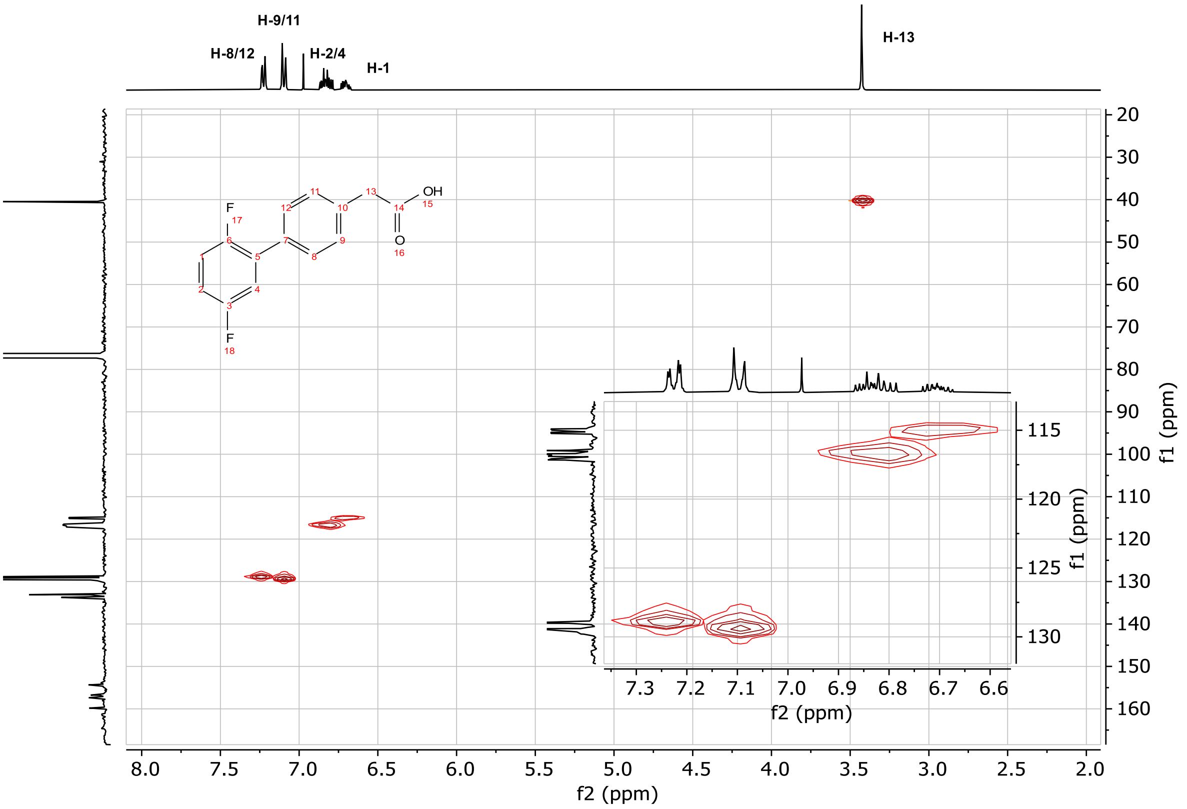

Figure S39: HSQC spectrum of **E9**.

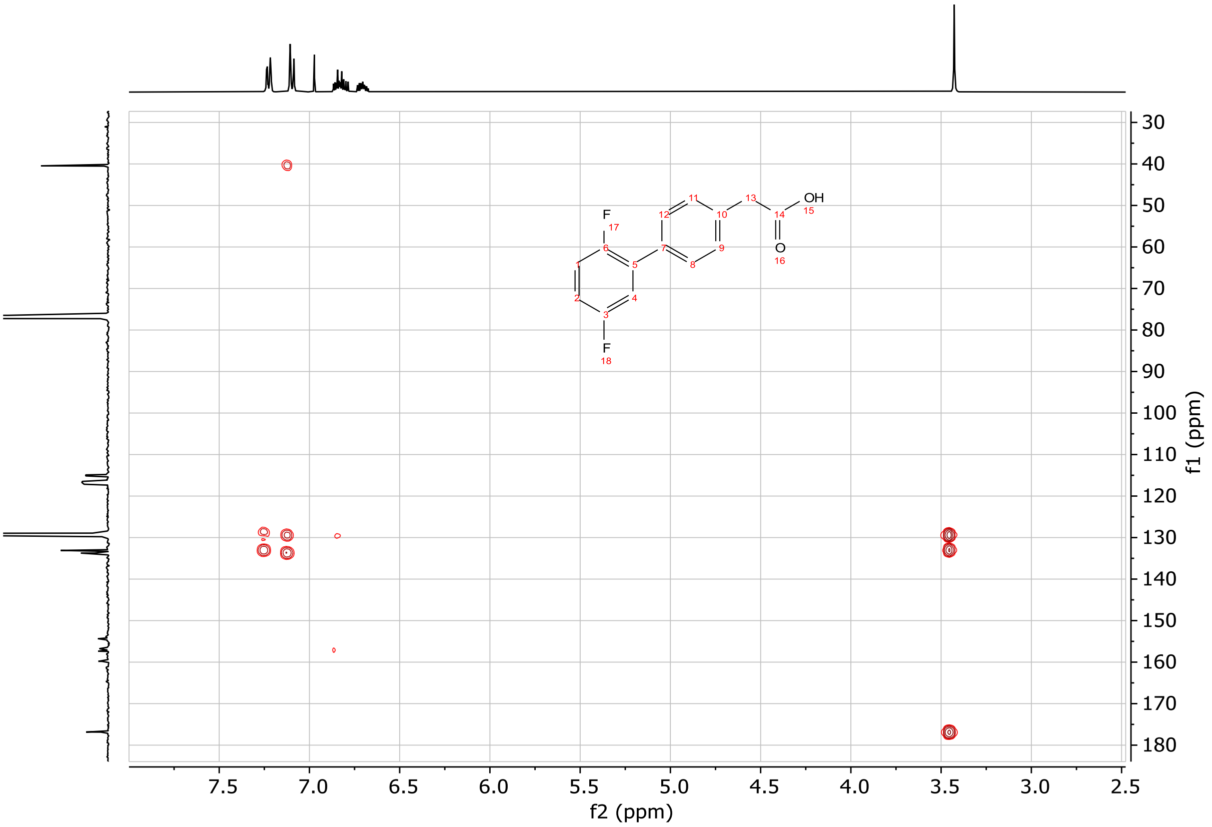

Figure S40: HMBC spectrum of **E9**.

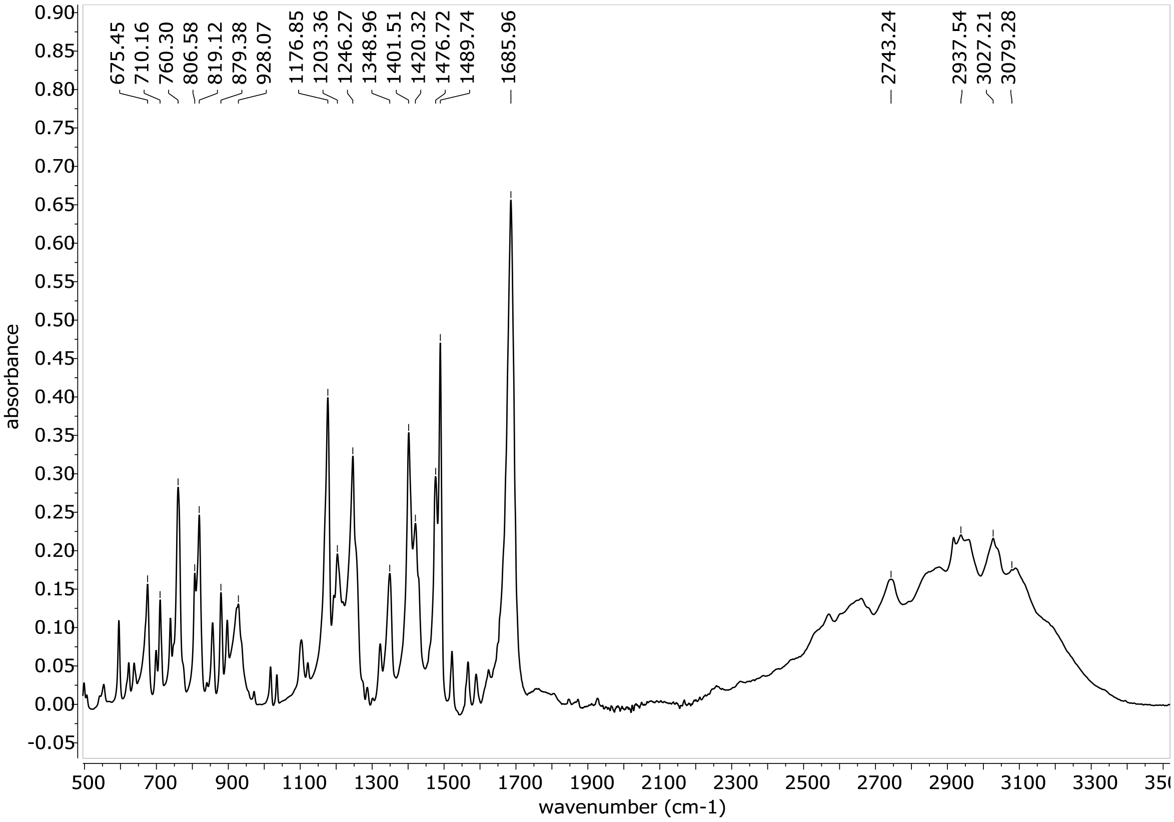

Figure S41: ATR-FTIR spectrum of **E9**.

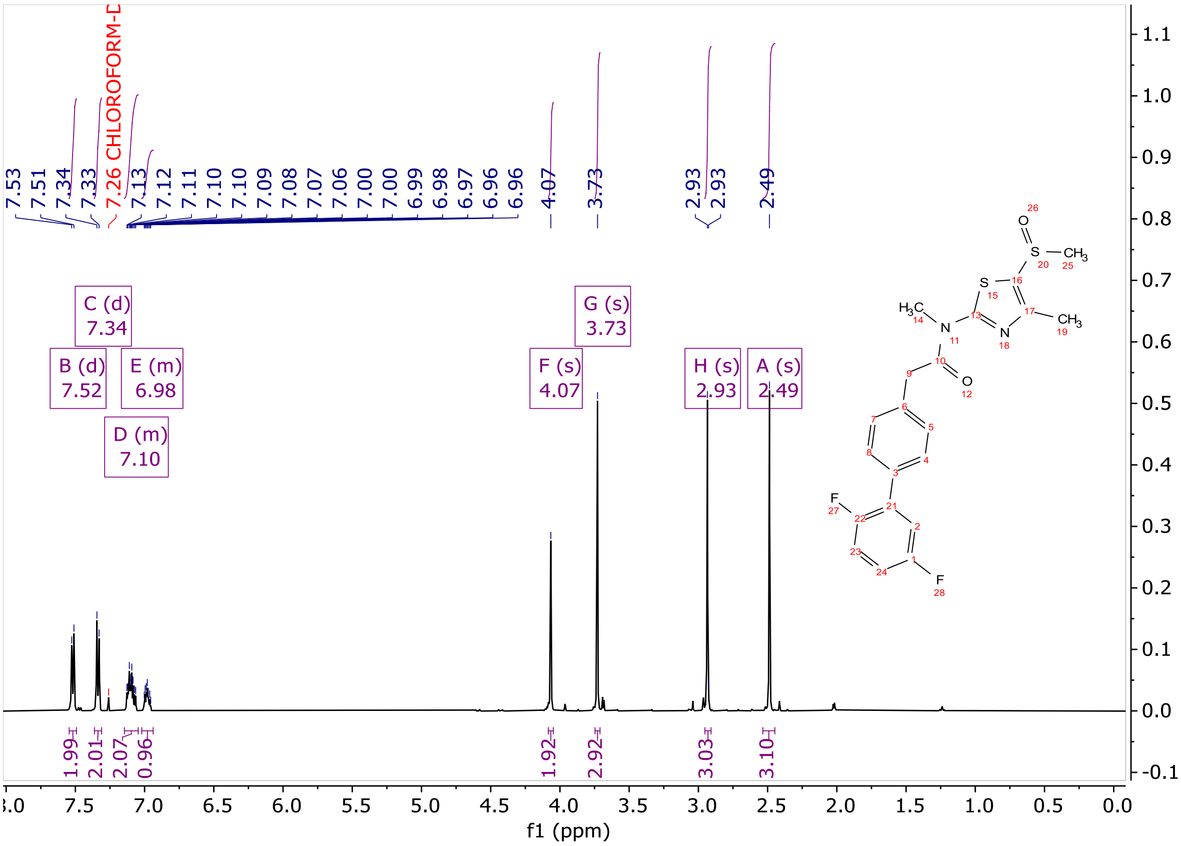

Figure S42: ^{1}H NMR spectrum of **E10**.

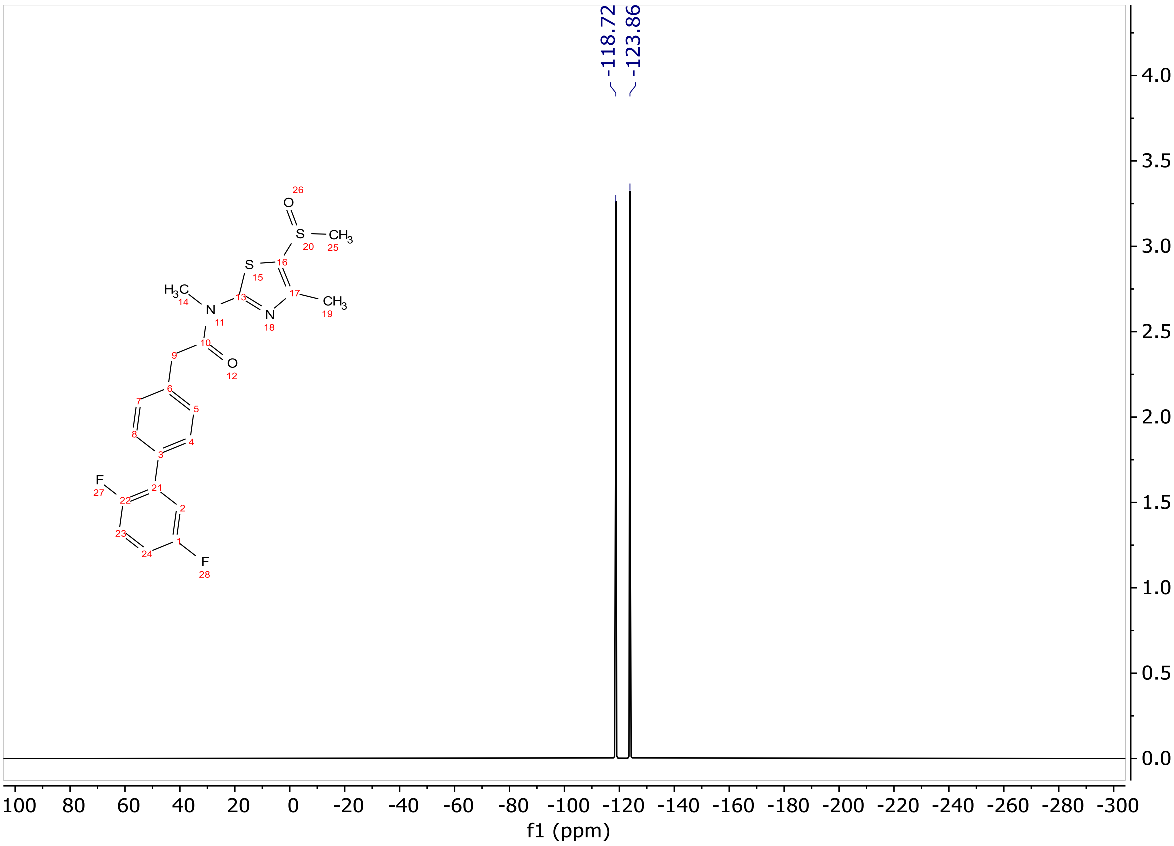

Figure S43: ^{19}F NMR spectrum of **E10**.

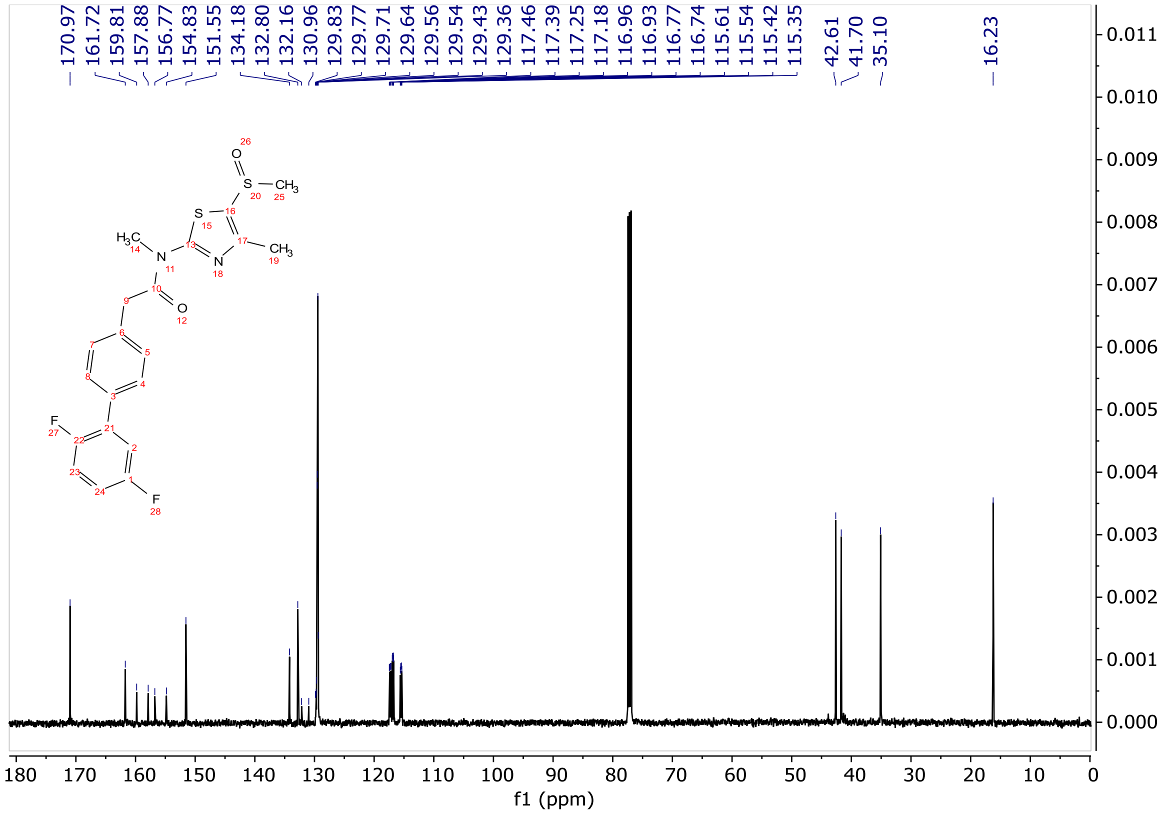

Figure S44: ^{13}C NMR spectrum of **E10**.

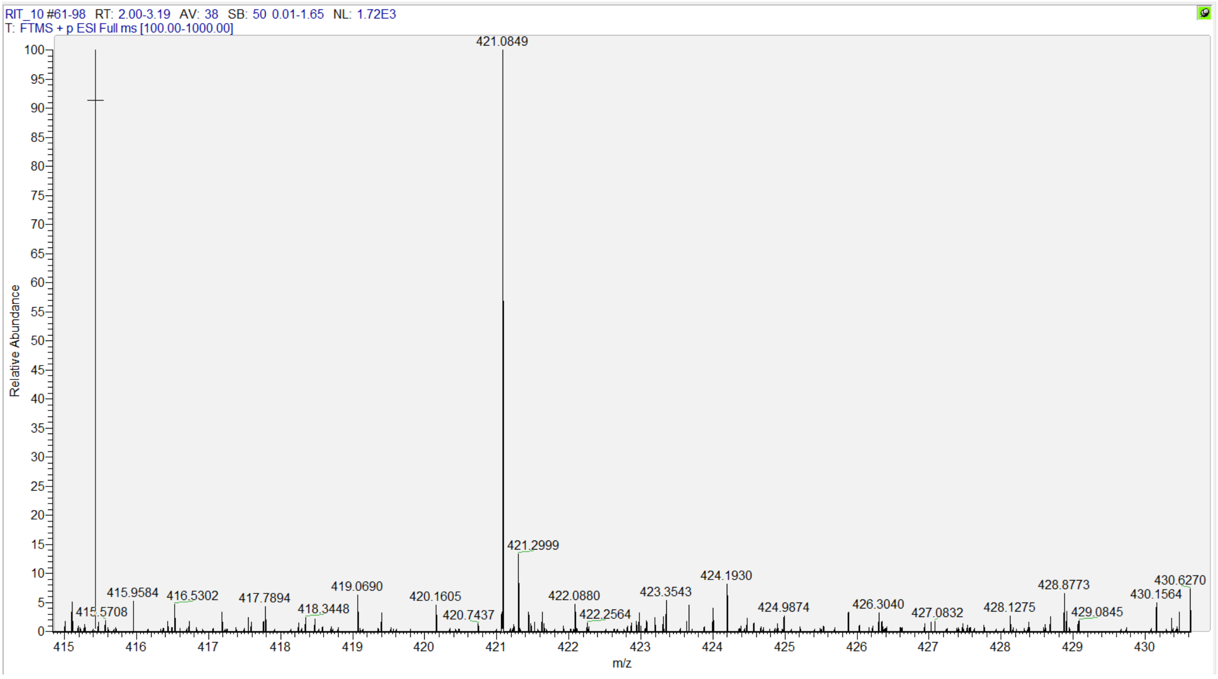

Figure S45: HRMS spectrum of **E10**. Calc: 420.0778 [M]; 421.0851 [M+H]. Found: 421.0849 [M+H].

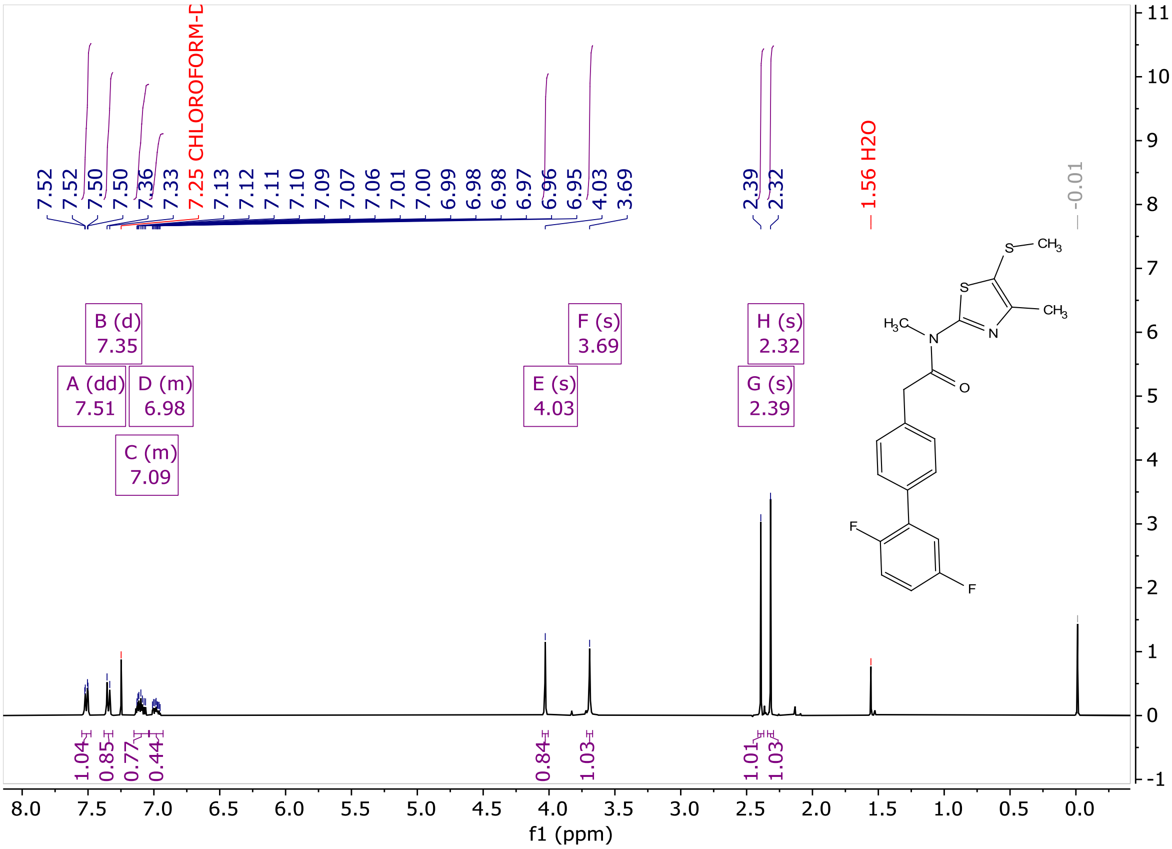

Figure S46: ^{1}H NMR spectrum of **F5**.

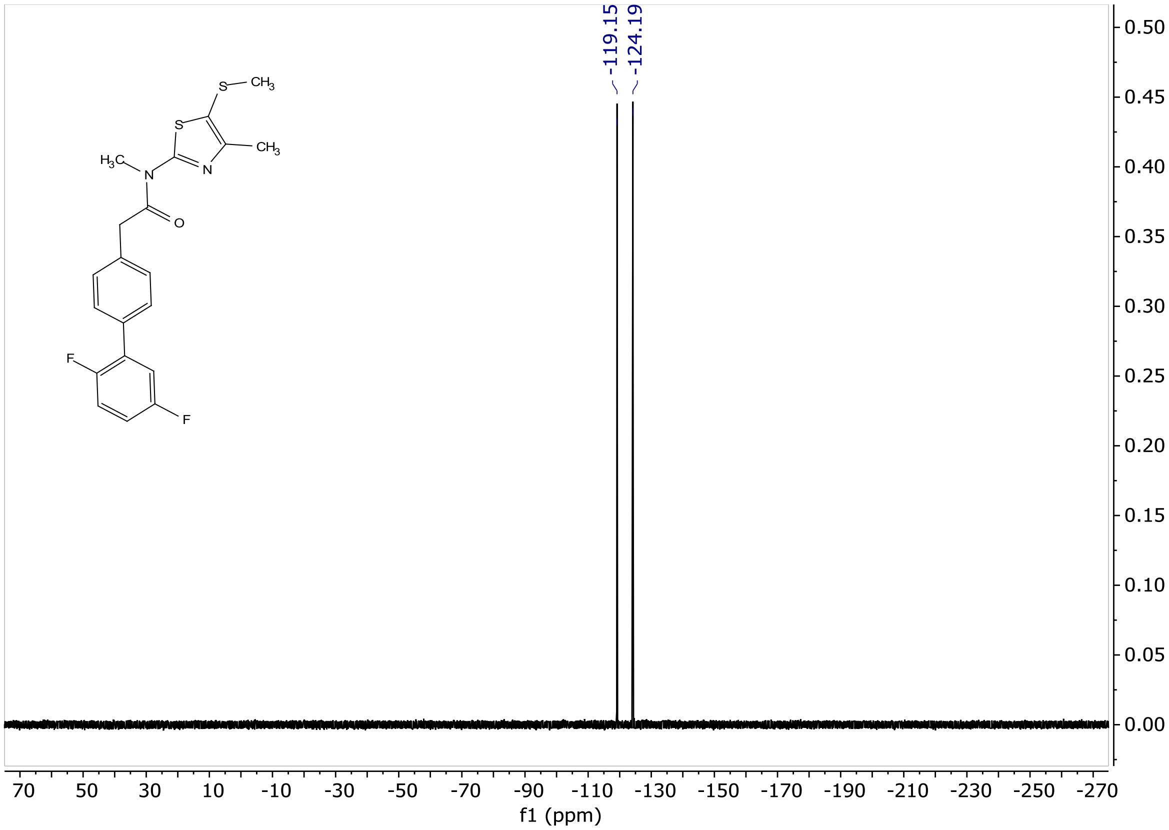

Figure S47: ^{19}F NMR spectrum of **F5**.

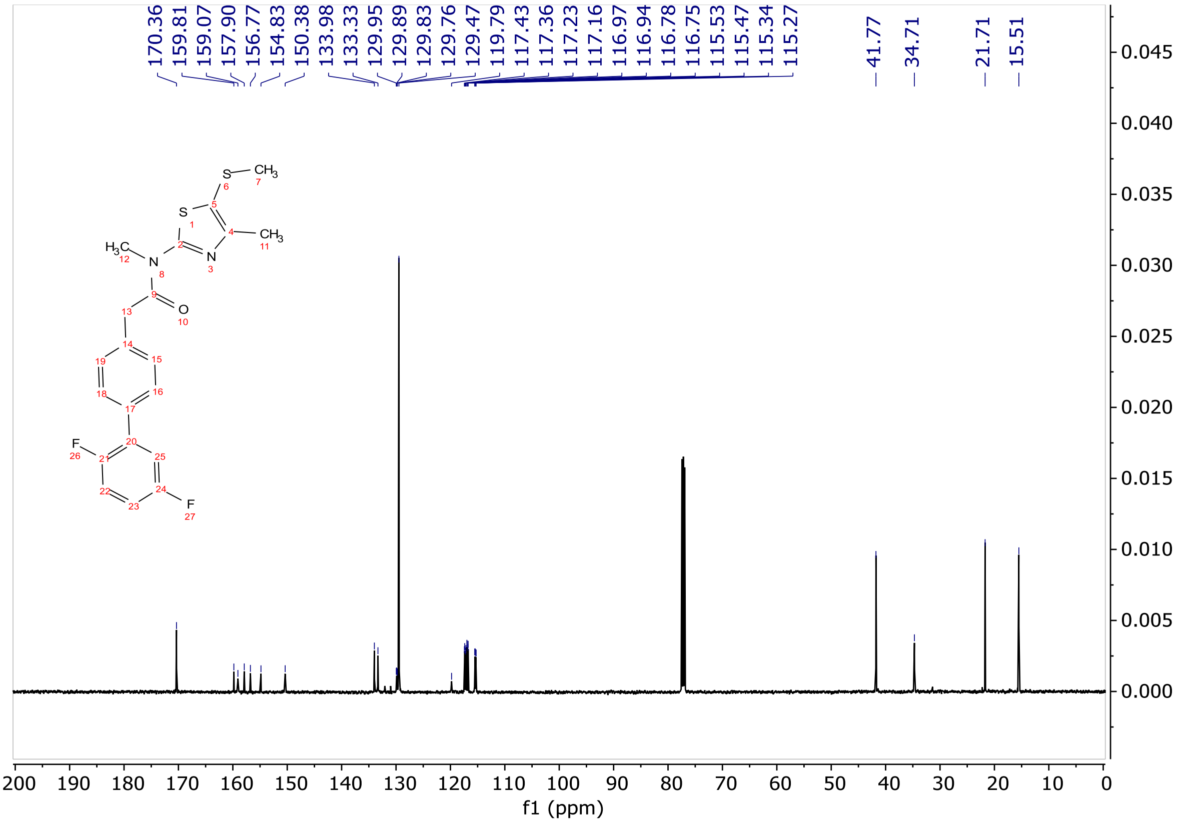

Figure S48: ^{13}C NMR spectrum of **F5**.

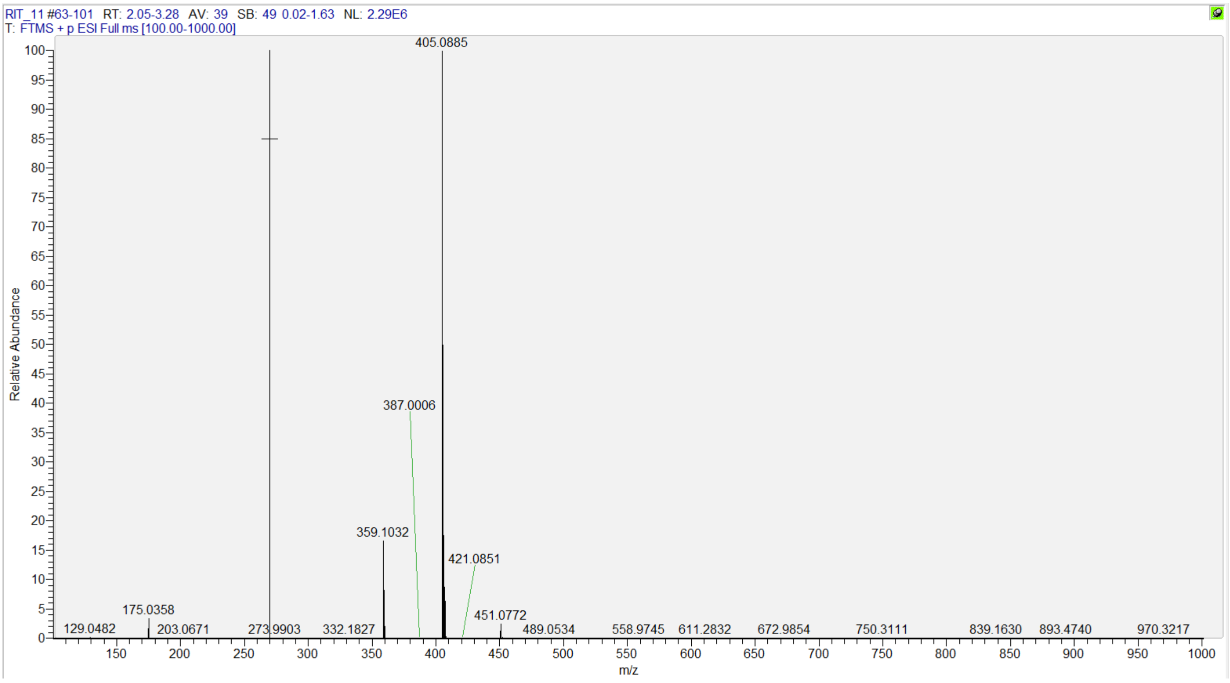

Figure S49: HRMS spectrum of **F5**. Calc: 404.0829 [M]; 405.0901 [M+H]. Found: 405.0885 [M+H]

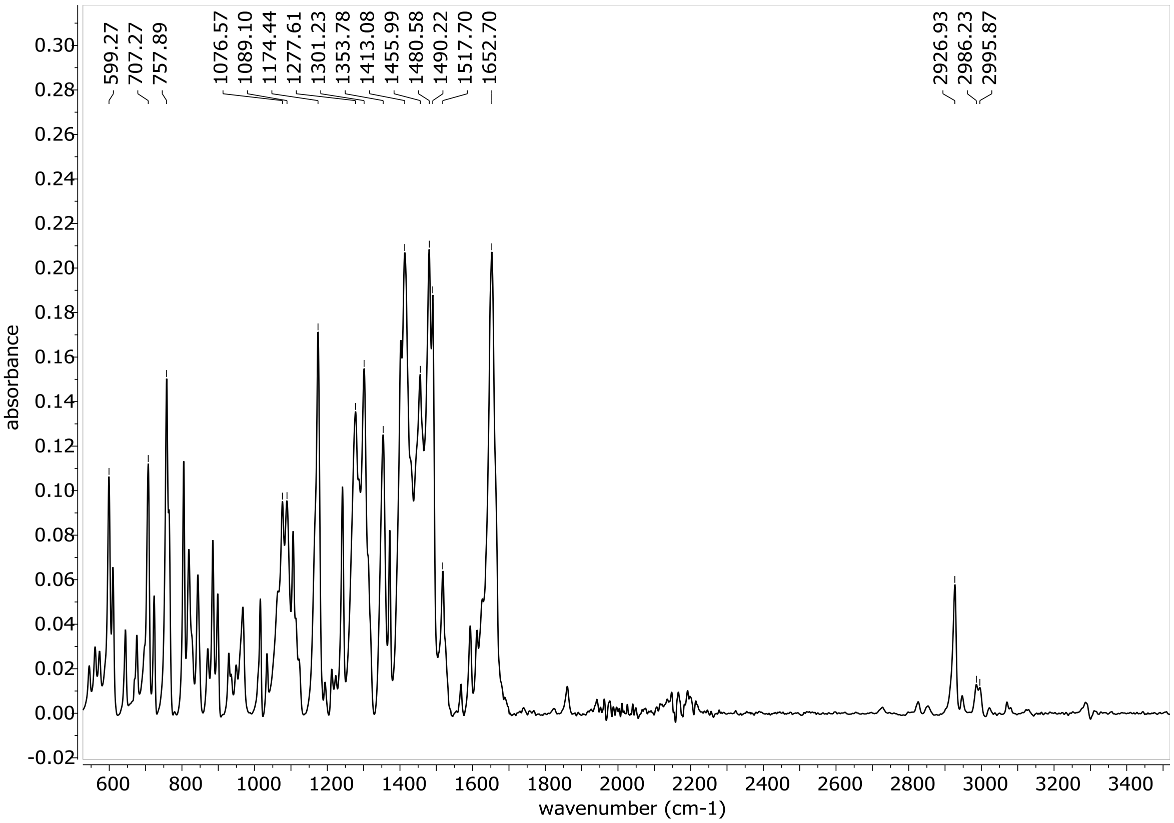

Figure S50: ATR-FTIR spectrum of **F5**.

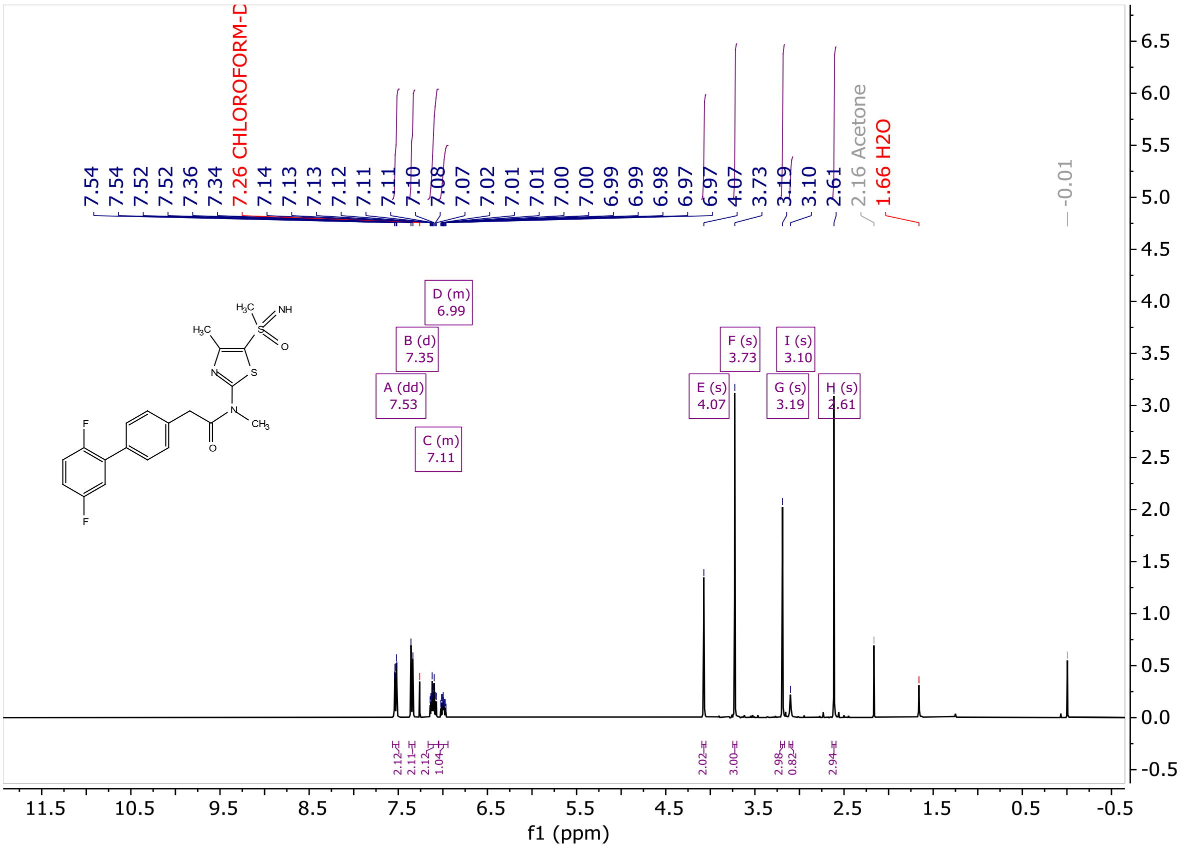

Figure S51: ^{1}H NMR spectrum of **IM-204**.

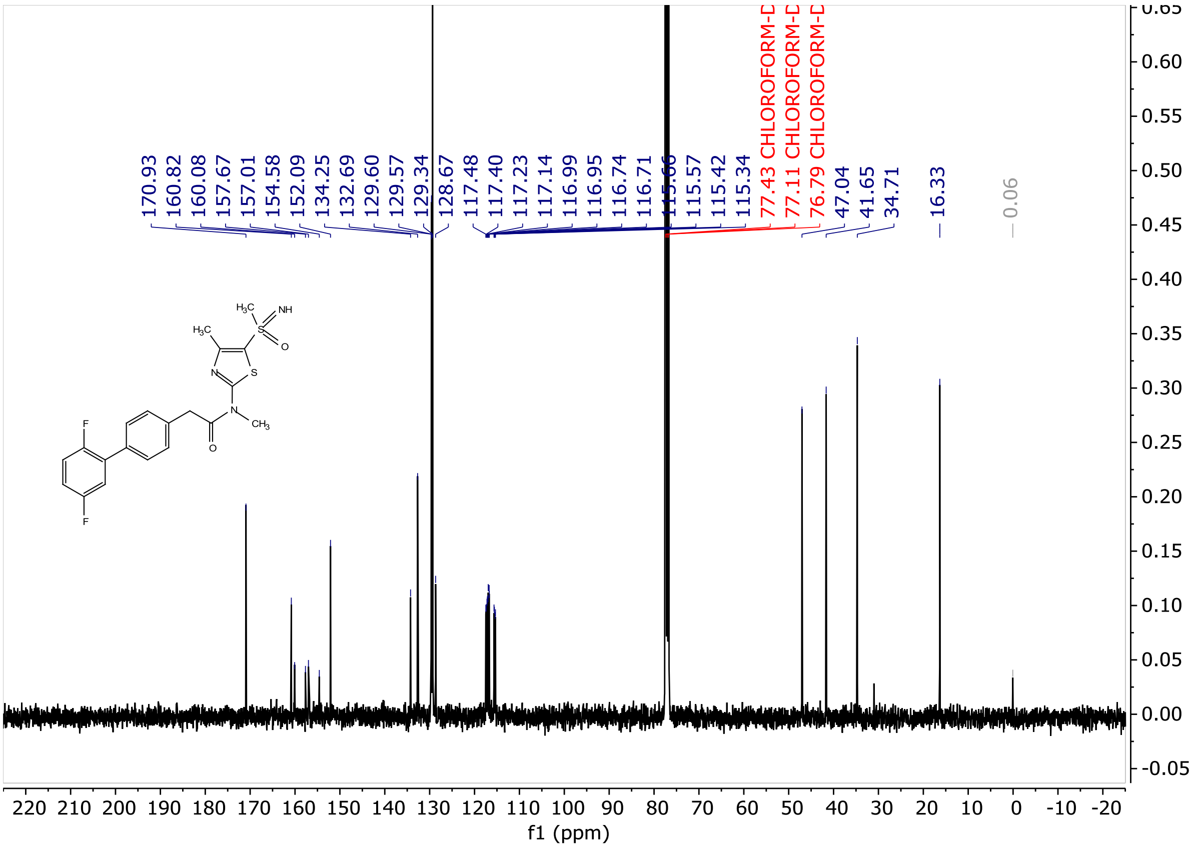

Figure S52: ^{19}F NMR spectrum of **IM-204**.

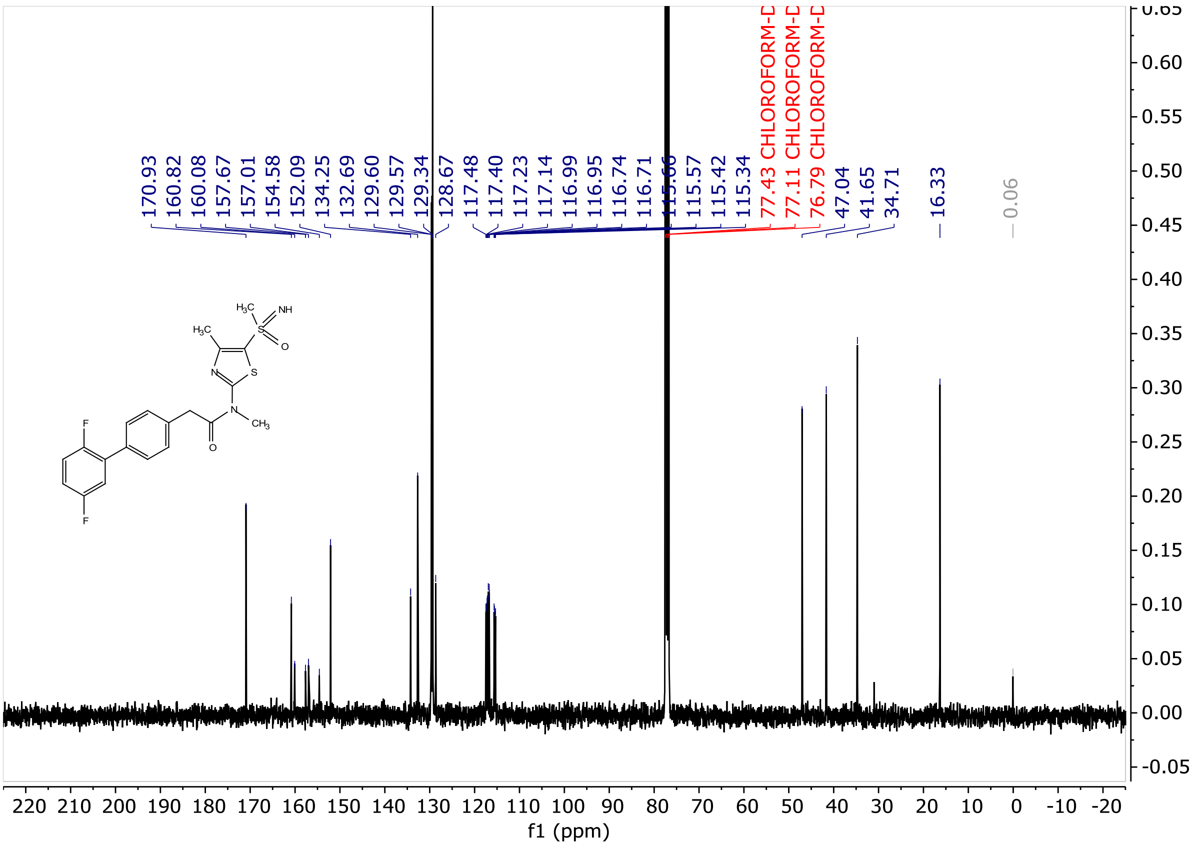

Figure S53: ^{13}C NMR spectrum of **IM-204**.

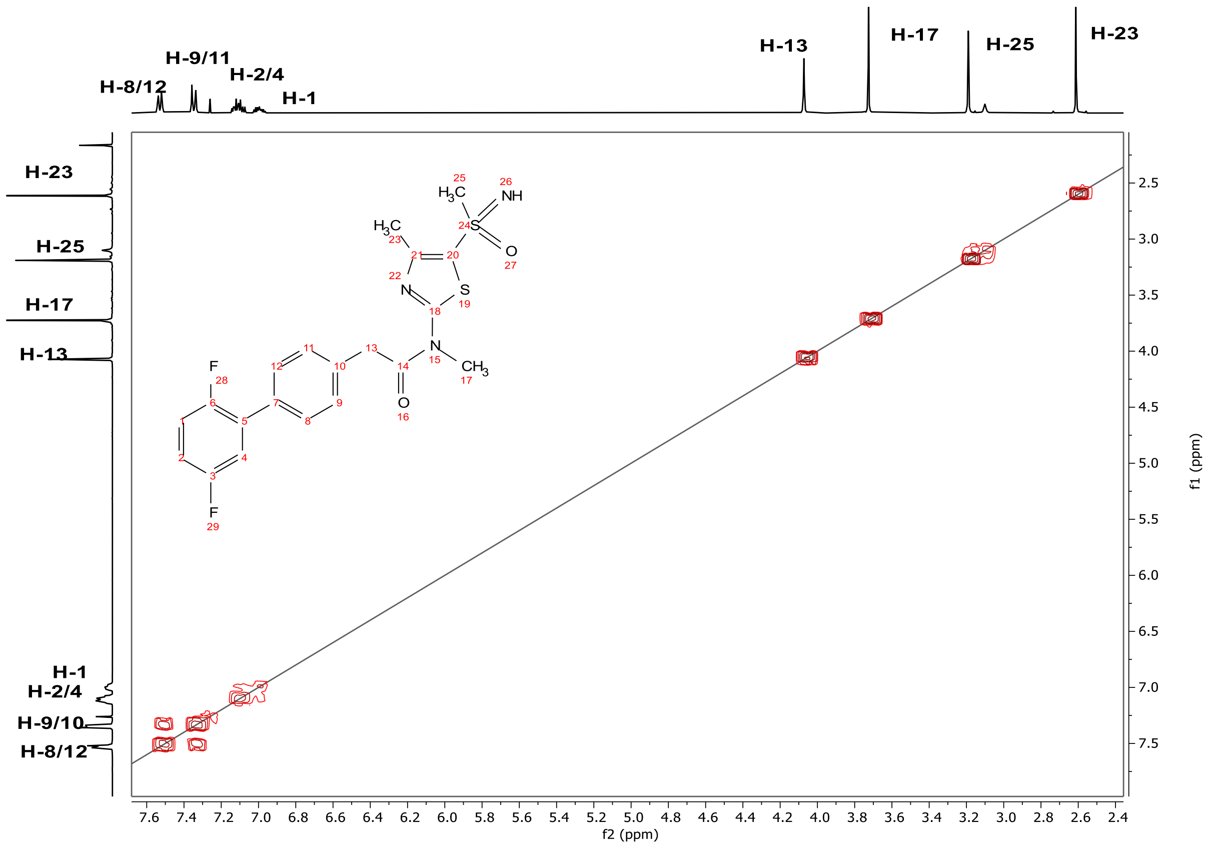

Figure S54: COSY spectrum of **IM-204**.

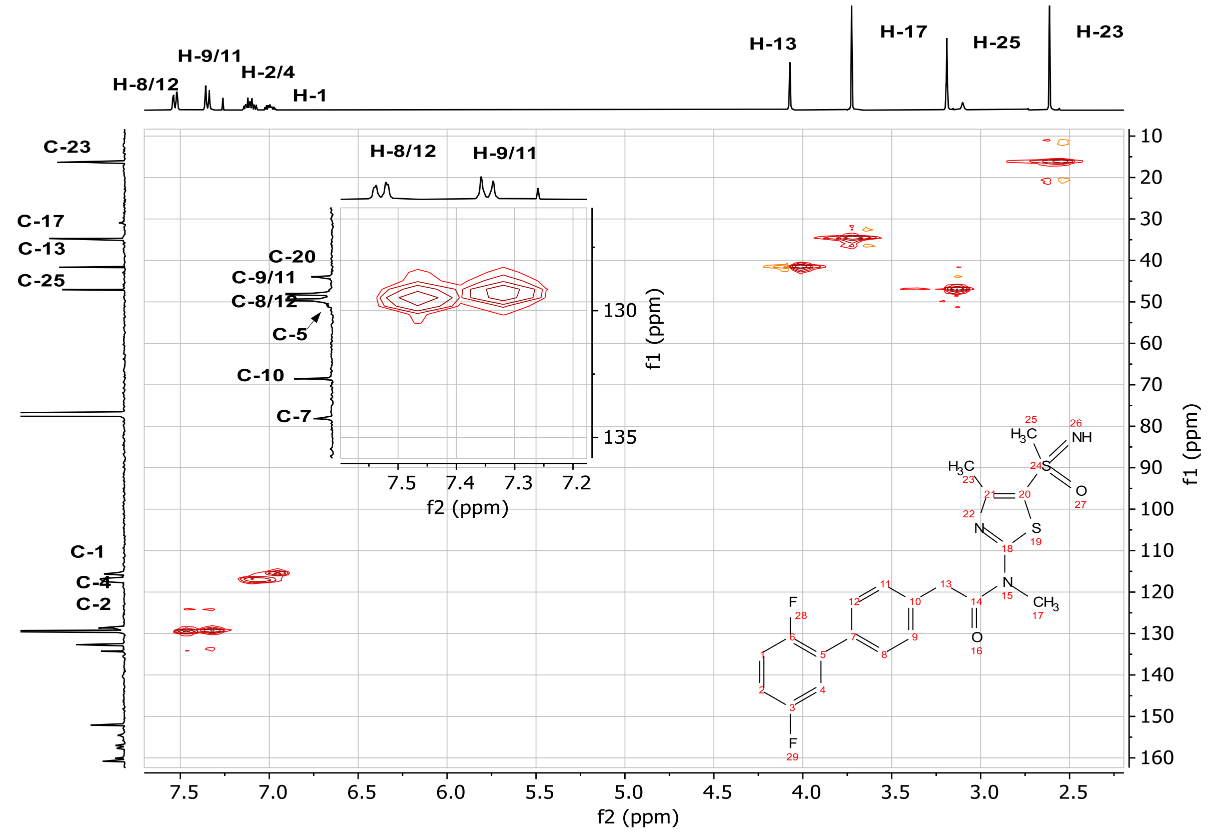

Figure S55: HSQC spectrum of **IM-204**.

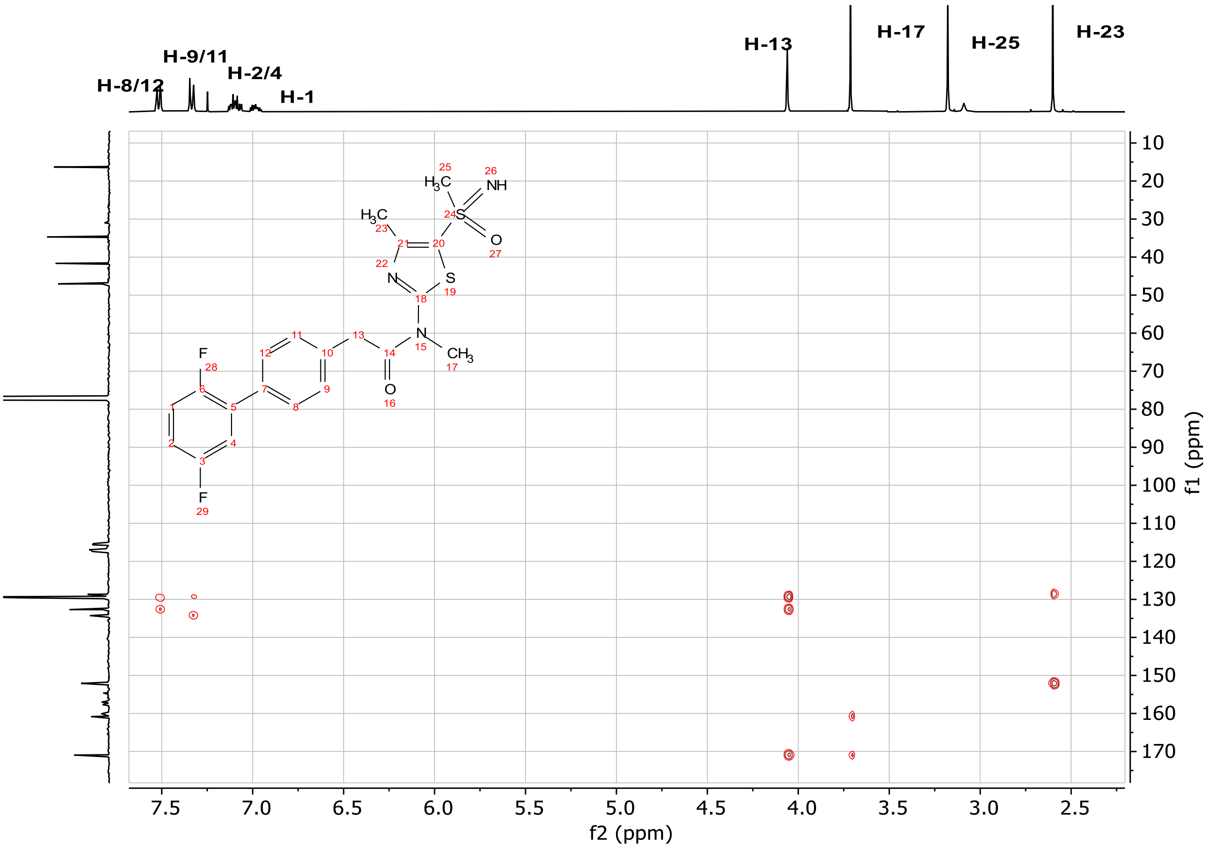

Figure S56: HMBC spectrum of **IM-204**.

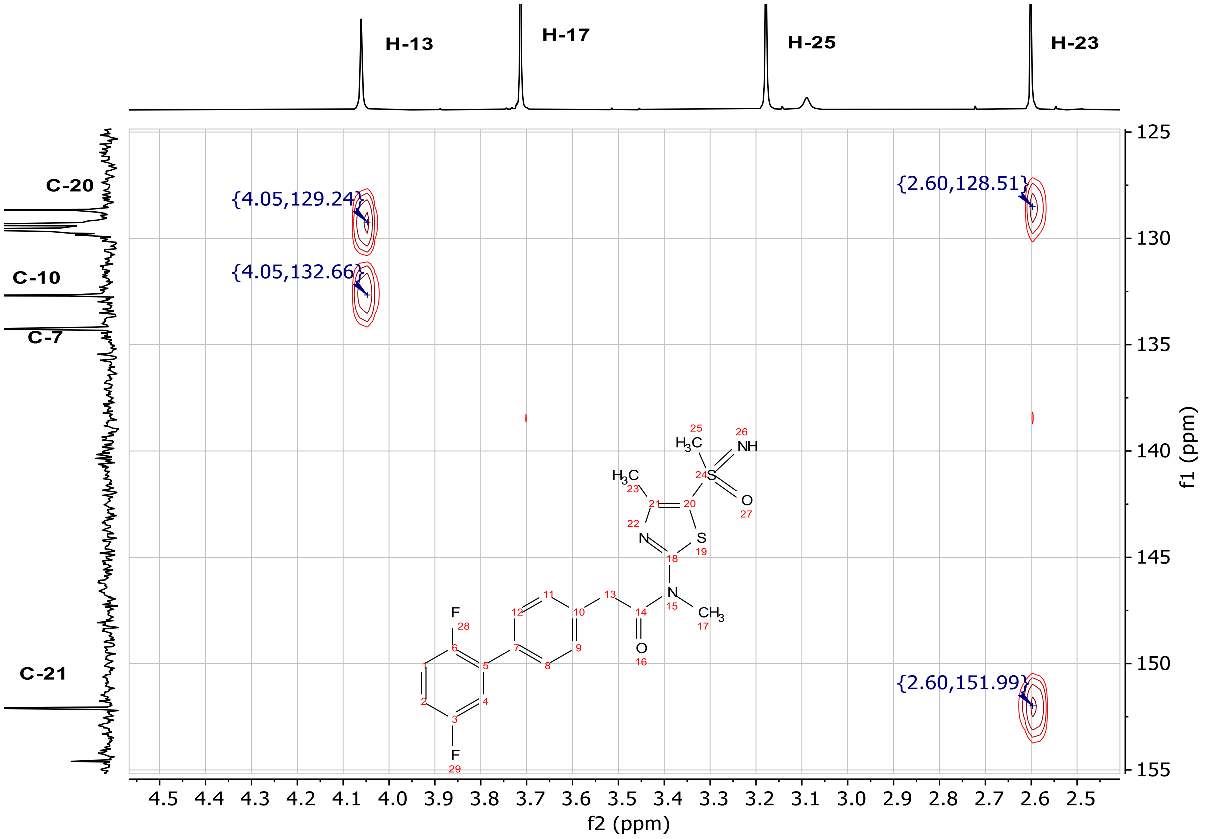

Figure S57: HMBC spectrum amplification (2-5 ppm ^{1}H x 125-155 ppm ^{13}C) of **IM-204**.

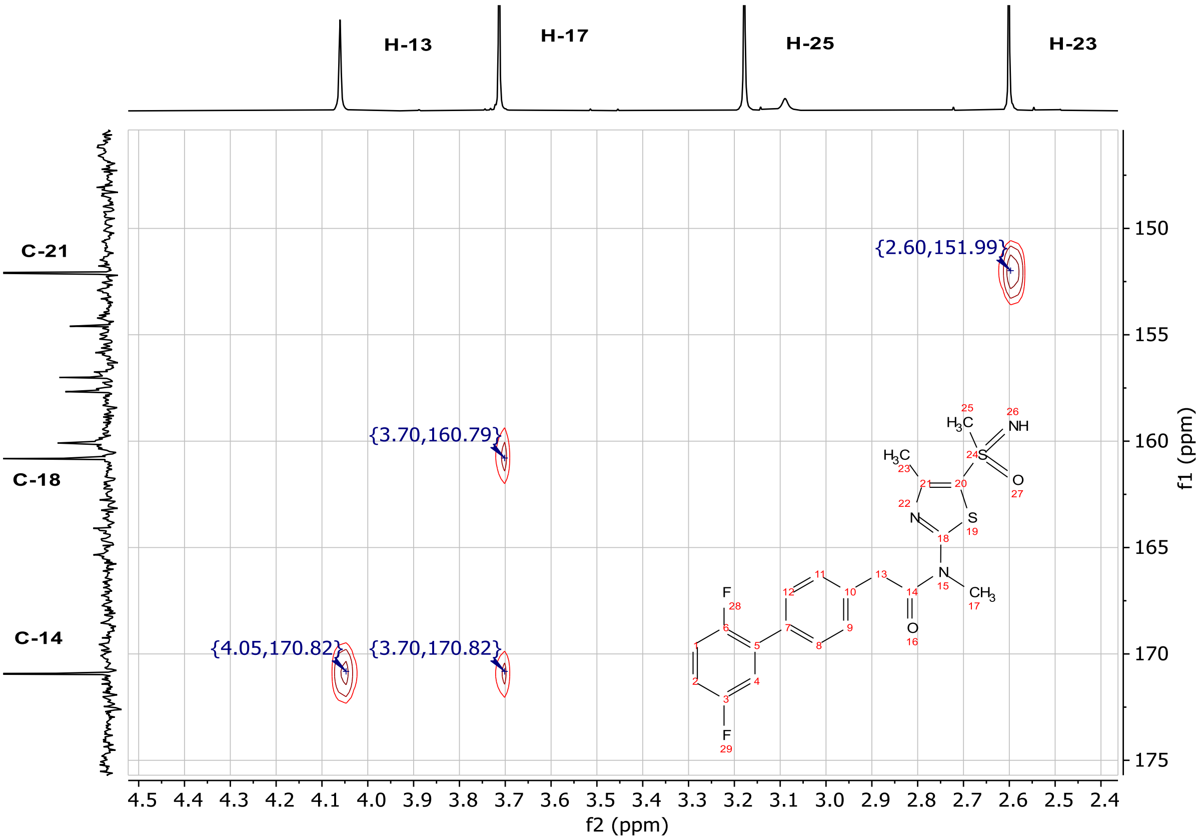

Figure S58: HMBC spectrum amplification (2-5 ppm ^{1}H x 145-175 ppm ^{13}C) of **IM-204**.

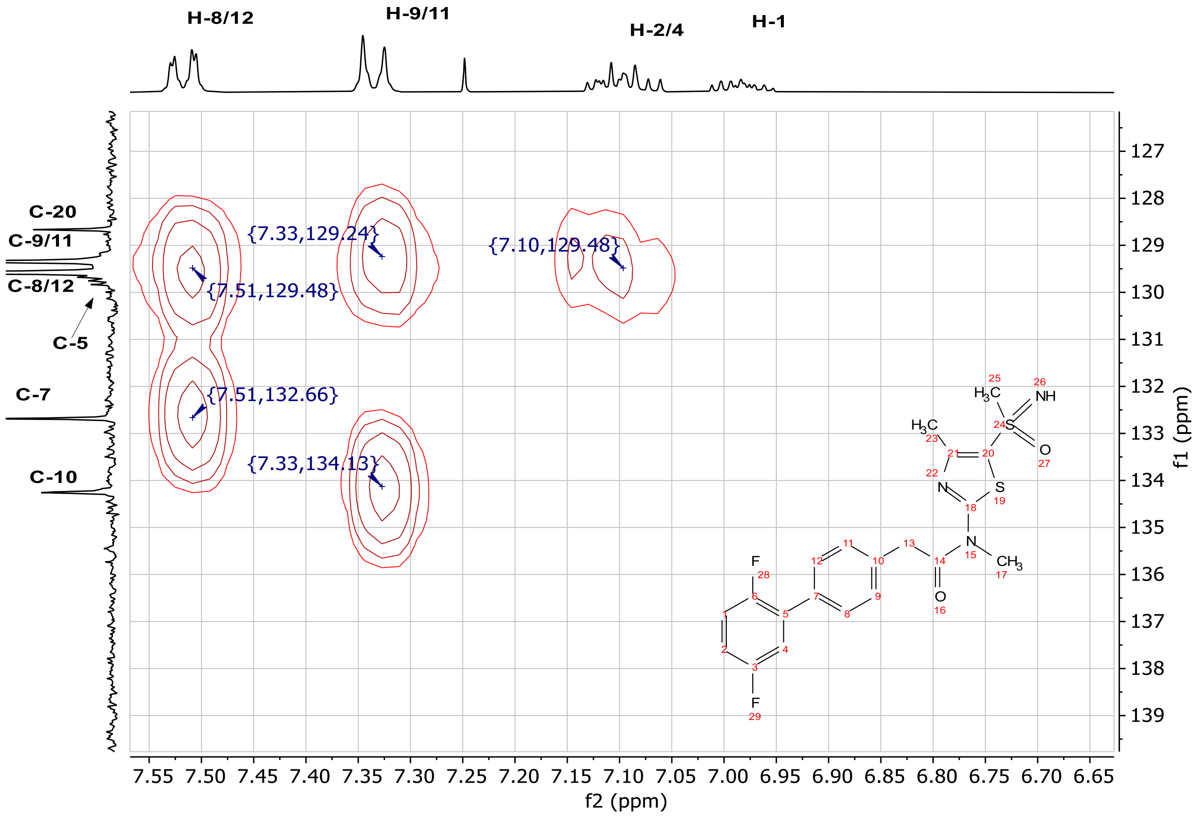

Figure S59: HMBC spectrum amplification (6-8 ppm ^{1}H x 125-140 ppm ^{13}C) of **IM-204**.

Table S2. Hetero- (homo-) nuclear J coupling, 1D and 2D ^{1}H and ^{13}C spectral interpretation of the IM-204 in $CDCl_3$.

	^{1}H	H-23	H-25	H-17	H-13	H-1	H-2/4	H-9/11	H-8/12
	δ H/^{13}C	2.61	3.19	3.73	4.07	6.99	7.11	7.35	7.53
^{13}C	^{1}H-^{1}H ^{3}J]	(s, 3H)	(s, 3H)	(s, 3H)	(s, 2H)	(m, 1H)	(m, 2H)	(d, 2H)	(dd, 2H)
C-23	16.33 s	1							
C-17	34.71 s			1					
C-13	41.65 s				1			3	
C-25	47.04 s		1						
C-1	115.50 dd					1			
C-4	116.85 dd						1		
C-2	117.31 dd						1		
C-20	128.67 s	3							
C-9/11	129.34 s				3			1	2
C-8/12	129.58 s							2	1
C-5	129.72 dd						2		
C-10	132.69 s				2				3
C-7	134.25 s							3	
C-21	152.09 s	2							
C-6	156.15 d								
C-3	158.60 d						2		
C-18	160.82 s			3					
C-14	170.93 s			3	2				

[a]HMBC (3 = ^{3}J; 2 = ^{2}J); HSQC (1 = ^{1}J) [b]See figure below for atomic numbering scheme. [c]quaternary carbon

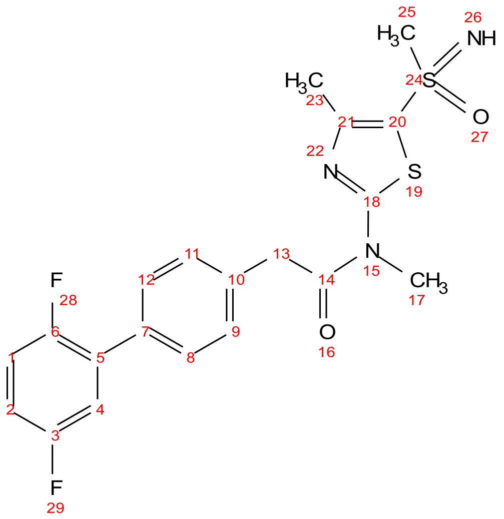

Figure S60. Atomic numbering of IM-204 for interpretation of the NMR spectra.

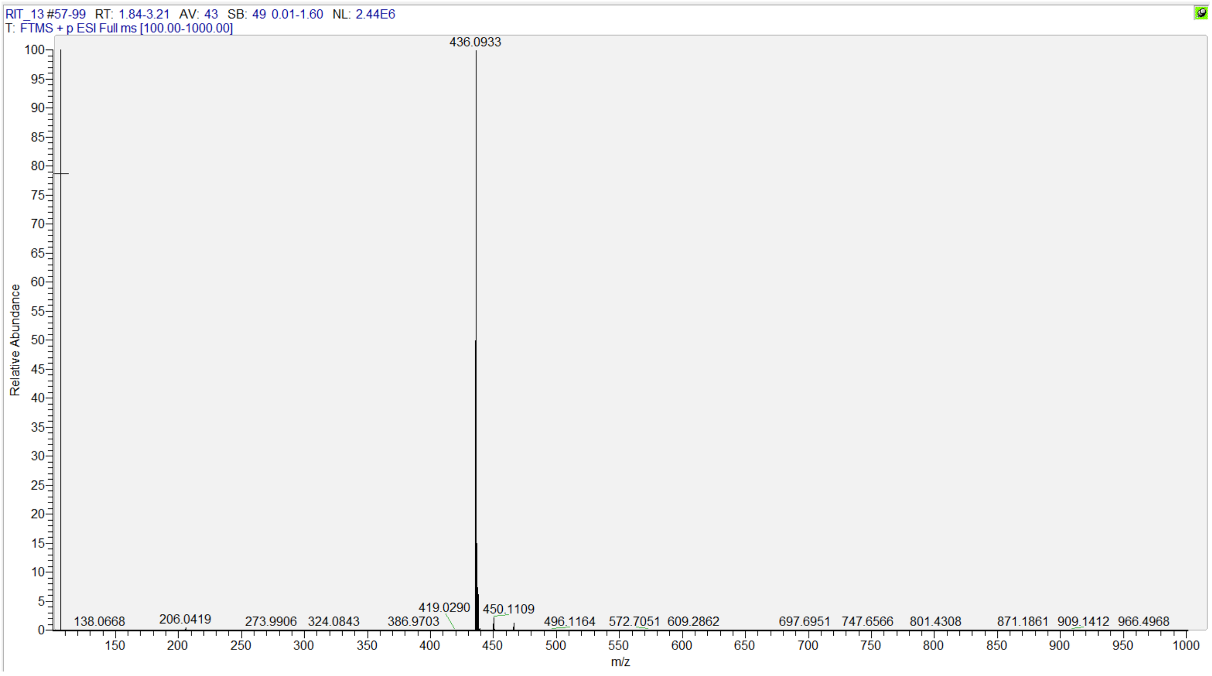

Figure S61: HRMS spectrum of IM-204. Calc: 435.0887 [M]; 436.0960 [M+H]. Found: 436.0933 [M+H].

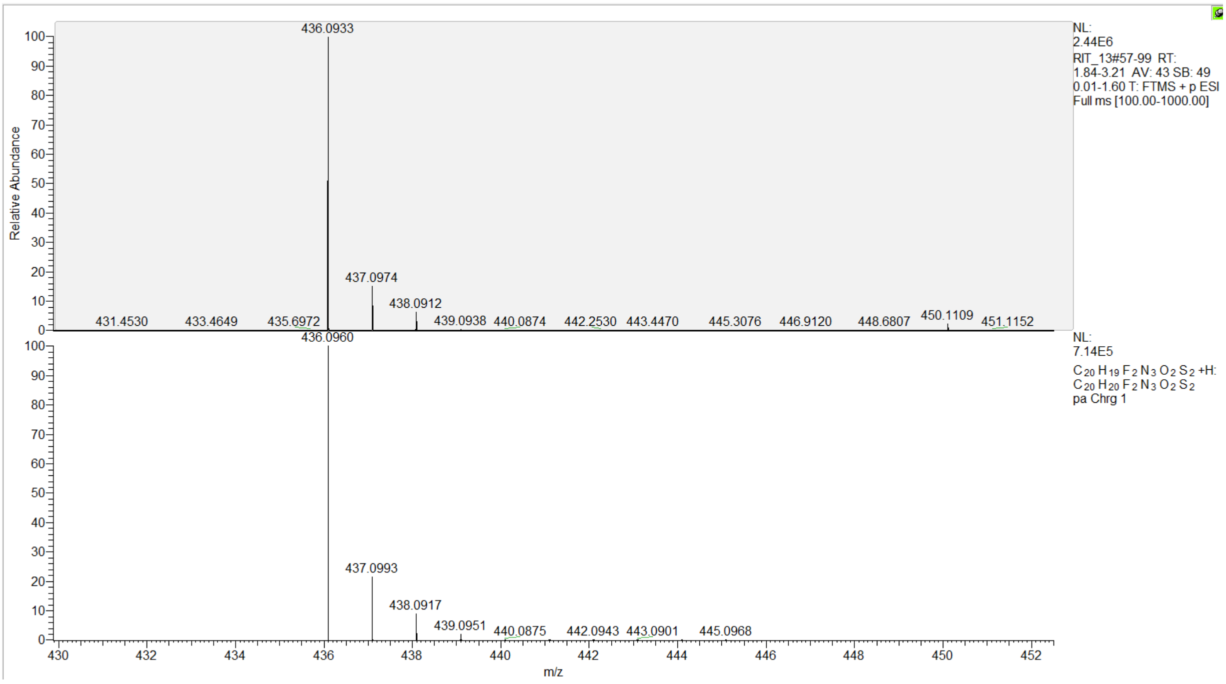

Figure S62: *Top:* Amplification of the HRMS spectrum of IM-204. *Bottom:* Software predicted isotope pattern for IM-204.

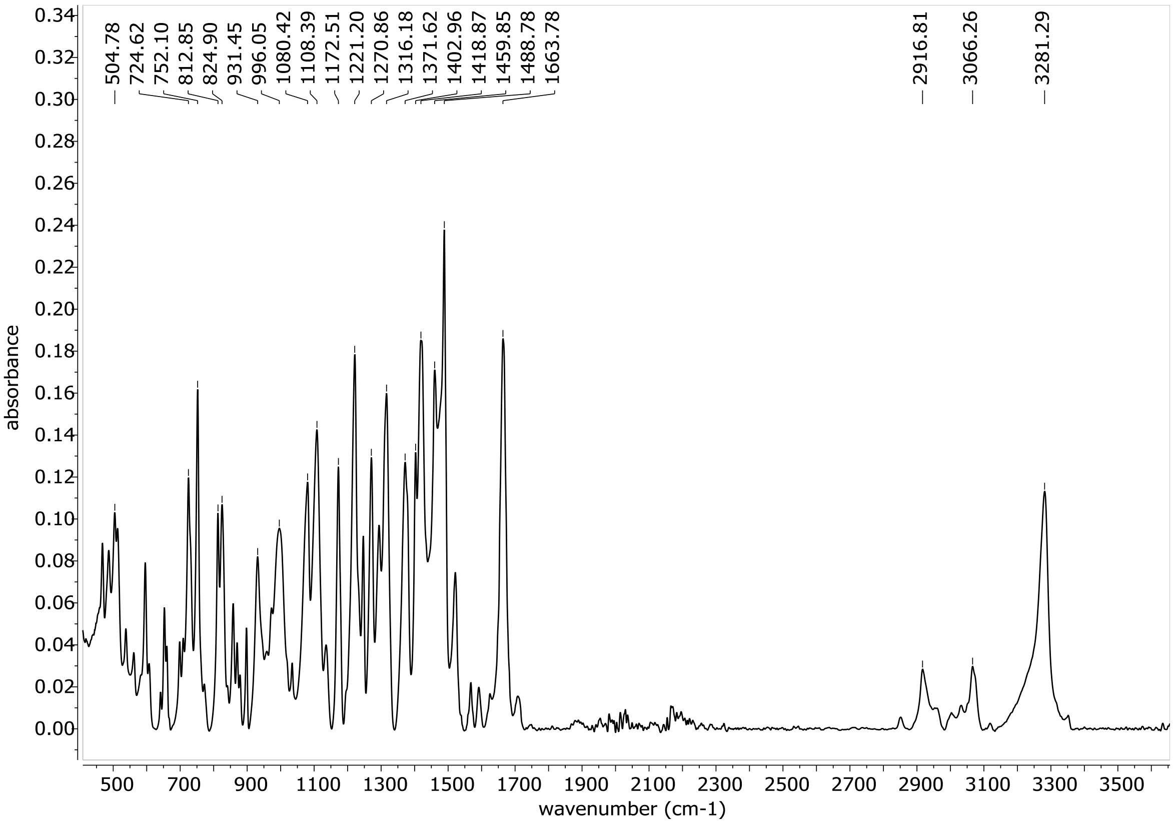

Figure S63: ATR-FTIR spectrum of IM-204.
